# Not that *cloze*: Sentence constraint is influenced by language and language dominance

**DOI:** 10.3758/s13428-026-03140-8

**Published:** 2026-10-14

**Authors:** Ana Bautista, Clara D. Martin

**Affiliations:** 1 BCBL, Basque Center on Cognition, Brain & Language, Paseo Mikeletegi 69, 20009 Donostia-San Sebastián, Spain; 2 University of the Basque Country (UPV-EHU), Bilbao, Spain; 3 Ikerbasque, Basque foundation for science, Bilbao, Spain

**Keywords:** Prediction, Cloze test, Sentence constraint, Cross-linguistic differences, Bilingualism

## Abstract

In the fields of language processing and prediction, cloze tests are used to establish sentence constraint norms. The validity of cloze test results depends heavily on how closely the pre-test population matches the main study population, and yet there is limited evidence on how sociolinguistic variables may influence sentence constraint. Here, we investigate how language and language dominance influence sentence constraint at the lexical level by conducting a cloze test across different language groups and comparing response entropy. In Analysis 1, sentence constraint was similarly high for Spanish- and French-dominant speakers tested on the same translated sentences. However, their item-by-item alignment was moderate. In Analysis 2, non-dominant French speakers showed lower sentence constraint than dominant speakers overall. Dominant and non-dominant French speakers showed only moderate alignment, whereas the strongest alignment was observed between the two groups of non-dominant French speakers with different dominant languages. Our findings suggest that sentence expectations are shaped by language and language dominance. These findings are discussed considering theoretical proposals on the cloze test procedure, and offer informed practices for designing future language prediction studies across diverse speaker populations.

**Supplementary Information:**

The online version contains supplementary material available at https://doi.org/10.3758/s13428-026-03140-8.

## Abstract in French

Dans les domaines du traitement du langage et de la prédiction linguistique, les tests de “cloze probability” sont utilisés pour établir des normes de contrainte des phrases. La validité des résultats obtenus à ces tests dépend largement du degré de correspondance entre la population évaluée lors du ”cloze test” et celle de l’étude principale. Pourtant, les données concernant l’influence des variables sociolinguistiques sur la contrainte des phrases restent limitées. Dans cette étude, nous examinons l’influence de la langue et de la dominance linguistique sur la contrainte des phrases au niveau lexical en administrant un ”cloze test” à différents groupes linguistiques et en comparant l’entropie des réponses. Dans l’Analyse 1, la contrainte des phrases était similairement élevée chez les locuteurs dominants en espagnol que chez les locuteurs dominants en français testés sur les mêmes phrases traduites dans chaque langue. Cependant, la correspondance item par item entre les deux groupes n’était que modérée. Dans l’Analyse 2, les locuteurs non dominants en français présentaient une contrainte des phrases globalement plus faible que les locuteurs dominants. La correspondance entre locuteurs dominants et non dominants en français n’était également que modérée, tandis que la plus forte correspondance était observée entre les deux groupes de locuteurs non dominants en français ayant des langues dominantes différentes. Nos résultats suggèrent que les attentes liées aux phrases sont façonnées par la langue et la dominance linguistique. Ces résultats sont discutés à la lumière des propositions théoriques relatives à la procédure du ”cloze test” et fournissent des recommandations éclairées pour la conception de futures études sur la prédiction linguistique auprès de populations de locuteurs diversifiées.

## Abstract in Spanish

En los campos del procesamiento del lenguaje y la predicción lingüística, las pruebas “cloze test” se utilizan para establecer valores normativos de restricción oracional. La validez de los resultados de estas pruebas depende en gran medida del grado de correspondencia entre la población evaluada en el “cloze test” y la población del estudio principal. Sin embargo, existe poca evidencia sobre cómo las variables sociolingüísticas pueden influir en la restricción oracional. En este trabajo, investigamos cómo la lengua y la dominancia lingüística influyen en la restricción oracional a nivel léxico administrando un “cloze test” a diferentes grupos lingüísticos y comparando la entropía de sus respuestas. En el Análisis 1, la restricción oracional fue igualmente alta en hablantes dominantes de castellano y de francés evaluados con las mismas oraciones traducidas en cada lengua. Sin embargo, la correspondencia ítem por ítem entre ambos grupos fue moderada. En el Análisis 2, los hablantes no dominantes de francés mostraron, en general, una menor restricción oracional que los hablantes dominantes. La correspondencia entre hablantes dominantes y no dominantes de francés también fue moderada, mientras que la mayor correspondencia se observó entre los dos grupos de hablantes no dominantes de francés que tenían diferentes lenguas dominantes. Nuestros resultados sugieren que las expectativas sobre la continuación de una oración están determinadas por la lengua y la dominancia lingüística. Estos hallazgos se discuten a la luz de las propuestas teóricas sobre el procedimiento del “cloze test” y ofrecen recomendaciones basadas en la evidencia para el diseño de futuros estudios sobre predicción lingüística en distintas poblaciones de hablantes.

## Introduction

Studies investigating the effects of language prediction as a comprehension process require sentence norms to verify that the materials are constraining enough to elicit prediction. To establish these norms, researchers have typically used the cloze task (Lahar et al., 2004; Taylor, 1953; Whitley et al., 2024). In this task, participants read sentence contexts and are asked to complete them with either the most predictable continuation or the first word(s) that come to mind. For instance, participants might be prompted to finish a sentence that allows for multiple plausible and acceptable continuations, such as “The boy went outside to play with a …”. By analyzing the answer distribution across participants, researchers can estimate both sentence constraint and the predictability of particular continuations in a given sentence context. A sentence is considered highly constraining if most participants propose the same word as a continuation, and less constraining if responses vary.

Traditionally, sentence constraint scores are extracted from cloze tasks based on the percentage of participants providing a particular response to an incomplete sentence, ranging from 0 to 100. This measure, commonly known as *cloze probability,* quantifies sentence constraint as the percentage of participants who provide the most frequent continuation (see Table 1). Some studies propose 37% as the maximum cloze probability for considering a sentence as low constraining, and 67% as the minimum for considering a sentence as highly constraining (Block & Baldwin, 2010). However, an inherent limitation of this approach is that it overlooks the full range of alternative responses provided by participants. In fact, an answer distribution of 60-10-10-10-10 (in which each number represents the percentage of participants giving a unique answer) or an answer distribution of 60-40 would lead to the same cloze probability score, although the variability in responses differs considerably (see Table 1).

**Table 1:** Cloze probability and response entropy scores for four hypothetical answer distributions in a cloze task

Sentence context	Answer distribution	Cloze probability	Response entropy
The boy went outside to play with a…	ball: 100%	100%	0
	ball: 60%	60%	0.97
	friend: 40%		
	ball: 60%	60%	1.77
	friend: 10%		
	kite: 10%		
	car: 10%		
	toy: 10%		
	ball: 20%	20%	2.32
	friend: 20%		
	kite: 20%		
	car: 20%		
	toy: 20%		

Another increasingly popular measure is *response entropy*. This measure of response uncertainty includes the distribution of probabilities of all possible answers provided after a sentence context, −Σ*p*(*x*) × log_2_*p*(*x*), where *p(x)* is the probability associated to each unique response. As explained in Lowder et al. (2018) and Whitley et al. (2024), entropy values are low when the participants’ answers coincide and high when multiple answers are provided with similar probabilities. A minimum response entropy value of 0 would occur when all participants provide the same continuation, indicating the lowest uncertainty about what follows the sentence context. Conversely, the maximum response entropy value, which is bounded by the total number of participants who complete the cloze task, occurs when each response provided is unique, reflecting the highest uncertainty about the upcoming word. Entropy provides a complementary measure of sentence constraint that captures the full distribution of response probabilities beyond the most frequent one (see Table 1). Studies have also suggested that entropy is a better predictor of sentence processing than cloze probability (Frank, 2013; Linzen & Jaeger, 2016; Lowder et al., 2018; but see also Levy et al., 2013).

Although the specific sentence constraint measure obtained from the cloze task is important, an even more crucial consideration is the participant population. Distinct participants should be tested in the norming and experimental studies to avoid memory effects or other confounding variables. However, to ensure that pre-test cloze task results are generalizable to the population tested in the main experiment, it is essential to collect pre-test data from a group that differs from, but is closely comparable to, the main study group on any variable that may influence norm ratings.

Studies testing prediction in non-native speakers of a language generally match the populations used in the cloze task to those tested in the main experiment. For instance, Martin et al. (2013) compared language prediction in Spanish–English late bilinguals and English native speakers in the main experiment and pre-tested their English materials (English sentences) on both populations. Ito et al. (2018) compared Japanese–English bilinguals and English native speakers, and also obtained cloze probability scores from both groups. Foucart et al. (2014) tested Spanish monolinguals, Spanish–Catalan early bilinguals and French–Spanish late bilinguals when reading Spanish sentences, and also pre-tested their materials with native and non-native Spanish speakers.

Initially, studies testing prediction in different age groups also matched the populations used in the cloze task to those tested in the main experiment. For instance, Federmeier et al. (2002) compared prediction abilities in younger and older adults and pre-tested their materials with comparable groups of younger and older adults using the cloze task. However, cloze task scores on their materials showed no age-related differences. Further, studies explicitly testing whether cloze task responses vary across age groups showed no age-related differences between young, middle-aged, and older adults (Lahar et al., 2004). Therefore, posterior studies were able to make informed decisions about which pre-test population to use. An example of this is Wlotko et al. (2012), who tested the prediction abilities of older adults compared to young adults but used cloze test answers from younger university students only.

Common intuition in studies on prediction in non-native speakers considers that cloze task answers are influenced by the participant’s proficiency in the language of testing. However, unlike in studies of age-related differences on prediction, this remains empirically untested. Understanding which aspects of language experience may influence cloze task answers is important for making informed decisions about the population on which to pre-test the materials. This is particularly relevant for research focusing on prediction in multilingual populations or those with diverse levels of proficiency. Comparing cloze task responses from groups that differ in language dominance would help determine whether proficiency truly affects cloze test responses, as prior research on age effects in language prediction has done.

Moreover, the traditional comparison between native and non-native groups is no longer the only approach to investigating bilingual language processing, nor the most informative. Recent trends in bilingual research encourage moving beyond monolingual normativity and adopting more robust designs (Kutlu & Hayes-Harb, 2023; Rothman et al., 2023). This involves comparing the processing of the second language (L2) of a bilingual speaker against their own first language (L1), or comparing language processing across different bilingual populations to better control for potential confounding variables, as in mirrored language groups (e.g., L1 Spanish – L2 English versus L1 English – L2 Spanish) or in multiple language pairings (e.g., L1 Spanish – L2 French versus L1 English – L2 French). In light of these proposed designs, it is important to examine not only whether language dominance affects cloze test responses, but also whether other sociolinguistic factors such as speakers’ linguistic background play a role.

As an alternative to running new cloze tests on the experimental materials used in a specific experiment, researchers can draw on existing datasets of normed sentences available across various languages and populations (e.g., Angulo-Chavira et al., 2024; Block & Baldwin, 2010; Bloom & Fischler, 1980; de Varda et al., 2024; Brunel et al., 2025; Frade et al., 2024; Peelle et al., 2020; Rossi et al., 2020; Whitley et al., 2024). In fact, given the growing interest in language prediction and the increasing number of studies and databases published with open materials, relying on previously normed sentences can often be the most practical and less time-consuming approach. However, without direct evidence on which factors shape cloze task responses, determining which existing normed materials are appropriate for a given experimental population remains a challenge. Moreover, as research increasingly includes a wider range of languages, many studies may make use of materials in other languages or translated versions. This, in turn, raises an important question: Do sentence materials preserve their original degree of constraint when translated to a different language?

To address this knowledge gap, we investigate whether sociolinguistic factors like language and language dominance modulate cloze test responses, and further consider how closely the norming population should match the main experiment population. Thus, the present study compared sentence constraint measures between different populations to test the effect of language (Analysis 1) and language dominance (Analysis 2). In Analysis 1, we examined whether French- and Spanish-dominant speakers have similar sentence constraint scores when completing identical sentences in their respective language. In Analysis 2, we examined whether language dominance influences sentence constraint by comparing French-dominant speakers with Spanish–French and English–French bilinguals completing sentences in French. In addition, we also share a public database of cloze task answers from four different populations of speakers to a set of 330 sentences, translated to English, French and Spanish. All items and their original answer distributions are provided in a simplified form in Appendix A, and the full version can be found in the project’s OSF repository (osf.io/k3ptv/). We believe these findings, along with the publicly available database, can offer meaningful methodological insights and resources for future studies on bilingual language prediction.

## Method

### Participants

Data were collected from four participant groups (see Table 2), recruited via Prolific, an online participant recruitment platform.

**Table 2:** Demographic and language proficiency characteristics of the participant groups

Variable	French-dominant group (*n* = 60)	Spanish-dominant group (*n* = 60)	Spanish–French bilingual group (*n* = 60)	English–French bilingual group (*n* = 60)	*p* value Analysis 1	*p* value Analysis 2
Gender (F/M)	19/41	24/36	44/16	37/21	.446	<.001
Age (years)	31.6 (7.5)	34.5 (8.6)	33.5 (9.9)	29.6 (5.8)	.051	.029
LexTALE (dominant language, %)	88.24 (7.18)	94.50 (5.15)	93.06 (5.33)	90.11 (10.86)	<.001	.005
LexTALE (non-dominant language, %)			62.04 (11.48)	63.14 (10.93)		.596

Age and LexTALE score are reported as mean (SD). Gender is reported as counts. Two participants in the English–French bilingual group identified as non-binary. Analysis 1 includes comparisons between the French- and Spanish-dominant groups. Analysis 2 includes comparisons between the French-dominant, Spanish–French, and English–French bilingual groups.

The first group consisted of 60 French-dominant participants recruited based on being born in France and reporting French as their first language. The second group consisted of 60 Spanish-dominant participants recruited based on being born in Spain and reporting Spanish as their first language.

Two additional bilingual groups were recruited. The third group consisted of 60 Spanish-dominant participants who reported being fluent, but not dominant, in French (hereafter Spanish–French bilinguals). These participants were recruited because they were born in Spain, had Spanish as their first language, and were fluent in French. The fourth group consisted of 60 English-dominant participants who reported being fluent, but not dominant, in French (hereafter English–French bilinguals). These participants were recruited because they were born in the United Kingdom, had English as their first language, and were fluent in French.

We excluded participants whose responses suggested insufficient engagement with the task (e.g., inconsistent or ungrammatical responses) and replaced them to maintain the target sample sizes. This resulted in the replacement of one participant in the French-dominant group, seven participants in the Spanish–French bilingual group, and eight participants in the English–French bilingual group.

Language proficiency was assessed using LexTALE tests in the relevant languages (Brysbaert, 2013; Izura et al., 2014; Lemhöfer & Broersma, 2012). Descriptive statistics for demographic and proficiency variables are reported in Table 2, together with *p* values for the group comparisons relevant to the planned analyses.

The groups included in Analysis 1 (French- and Spanish-dominant groups) differed significantly in LexTALE scores in their respective dominant languages (French < Spanish; *p* <.001). This pattern is consistent with the original LexTALE norming studies, where native speakers of Spanish obtained higher mean scores on the test (*M* = 94.92%; Izura et al., 2014) than native speakers of French (*M* = 88.21%; Brysbaert, 2013). This suggests that observed differences likely reflect systematic differences in LexTALE score distributions across language versions, rather than clear differences in participants’ underlying proficiency or dominance.

The groups included in Analysis 2 (French-dominant, Spanish–French, and English–French bilingual groups) differed in gender distribution: pairwise comparisons revealed significant differences between the French-dominant and Spanish–French bilingual group (*p* <.001), and between the French-dominant and English–French bilingual group (*p* =.006). Age differed significantly only between the Spanish–French and English–French bilingual groups (*p* =.045). LexTALE scores in the groups’ dominant languages were lower in the French-dominant group than in the Spanish–French group (*p* =.003), again consistent with potential differences in LexTALE score distributions across languages. Crucially, the two bilingual groups did not significantly differ in their French LexTALE scores (*p* =.596), indicating comparable proficiency in the language relevant to the experimental task.

### Materials

We prepared 330 sentence contexts designed to be highly constraining toward a final noun phrase consisting of a noun preceded by an article, which could be definite, indefinite, or possessive. Sentence materials were adapted from previous psycholinguistic studies and norming datasets (e.g., Altarriba et al., 1996; Block & Baldwin, 2010; Brunel et al., 2025; DeLong et al., 2005; Federmeier & Kutas, 1999; Foucart et al., 2014; Martin et al., 2013; Wicha et al., 2003), with additional items newly created by the authors (native speakers of the respective languages) to meet the specific constraints of the study. During construction, sentences were designed to bias toward a specific lexical continuation in both languages, although their actual level of constraint was later empirically assessed through the cloze test. For example, “On farms, to get milk, it is necessary to milk…” strongly constrained the completion “a cow”. Each sentence context had a French and Spanish translation equivalent, with the target ending noun phrase also being a translation equivalent across the two languages.

Translation equivalents of the sentence contexts were longer in French (*M* = 13.78 words, *SD* = 4.05) than in Spanish (*M* = 12.50 words, *SD* = 3.62) (*t*(329) = – 12.13, *p* <.001, *d* = – 0.67), as it typically happens for sentence materials of this kind (InterContact, 2022). The intended target noun was similar across languages in length, measured in number of characters (*M*_*French*_ = 6.28, *SD*_*French*_ = 2.11, *M*_*Spanish*_ = 6.23, *SD*_*Spanish*_ = 1.90, *t*(329) = – 0.53, *p* =.600, *d* = –0.03), and in lexical frequency, obtained as frequency-per-million from CLEARPOND (Marian et al., 2012) (*M*_*French*_ = 53.94, *SD*_*French*_ = 79.93, *M*_*Spanish*_ = 58.91, *SD*_*Spanish*_ = 128.65, *t*(306) = 1.01, *p* =.312, *d* = 0.06). Sentence contexts in each language were randomly divided into three lists of 110 items each. All items and their answer distributions are summarized in Appendix A, with the full version accessible in the OSF repository (osf.io/k3ptv/).

### Procedure

**Fig. 1:**
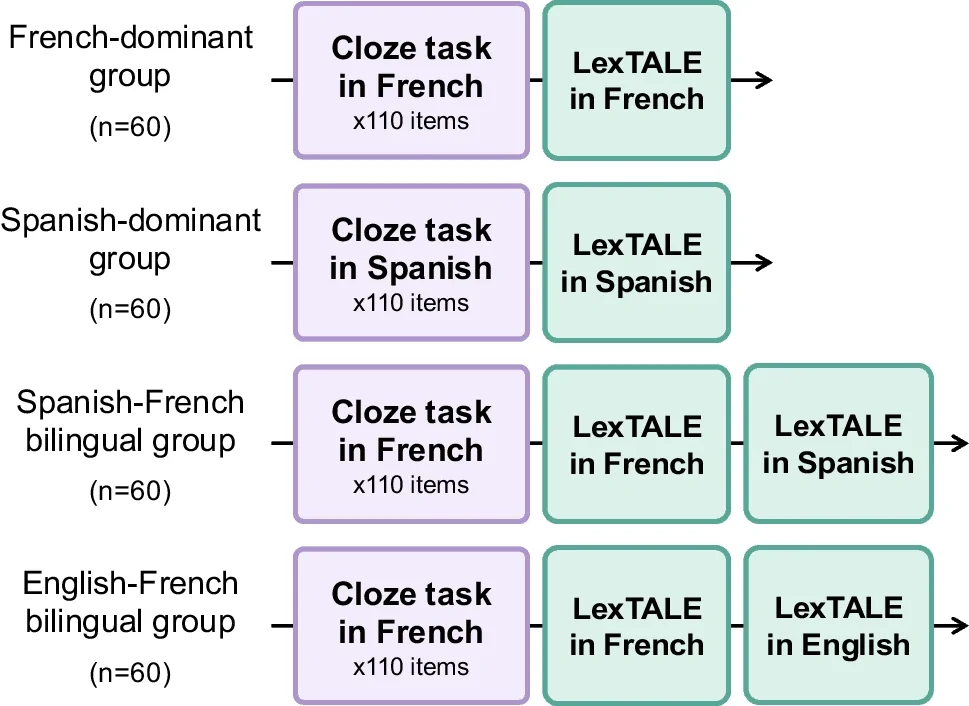
Overview of the experimental procedure across participant groups

Participants were recruited through Prolific to complete an online study created and hosted in Gorilla (www.gorilla.sc; Anwyl-Irvine et al., 2020). The study procedure has been made publicly available (https://app.gorilla.sc/openmaterials/820395). A schematic overview of the experimental procedure and language assignment across participant groups is shown in Fig. 1.

Participants completed a cloze task for one list of stimuli, consisting of 110 sentence contexts. The stimuli list was administered in French for French-dominant participants, in Spanish for Spanish-dominant participants, and in French for both bilingual groups (Spanish–French and English–French). They were instructed to complete each sentence with the first continuation that came to mind. This way, we obtained 20 answers for each sentence context in each language group, consistent with standard cloze norming procedures in psycholinguistics, which typically rely on approximately 10–20 responses per item to estimate sentence constraint measures (e.g., Federmeier et al., 2002; Ito et al., 2018; Martin et al., 2013).

The duration of the cloze task was 23 min on average for the French-dominant group, 21 min for the Spanish-dominant group, 55 min for the Spanish–French bilingual group, and 58 min for the English–French bilingual group. Participants were able to take a break every 30 sentences.

After completing the cloze task, participants completed the LexTALE test(s) in the relevant language(s) (see Fig. 1). All instructions and communication with participants were conducted in the language of the experiment.

### Data pre-processing

Answers were first processed to remove special characters, accents, capitalization, and punctuation. They were then processed using the spaCy library in Python (Honnibal & Montani, 2017) to extract the first noun mentioned by the participants to assess lexical sentence constraint.

The extracted nouns were then corrected for misspellings. Differences in plurality and suffixes were treated as the same word – for example, “fenêtres” (‘windows’) in French was adapted to “fenêtre” (‘window’), and “figurita” (‘little figure’) in Spanish to “figura” (‘figure’). Abbreviations were expanded, such as “tv” to “televisión” (‘television’), and hyphenated forms were standardized, like “cerf volant” to “cerf-volant” (‘kite’). These adaptations were made using the Collins Dictionary for French (Collins, n.d.) and the RAE dictionary for Spanish (Real Academia Española, 2024), to ensure a consistent use of nouns across responses. If participants did not provide a noun or provided a noun that was not a valid word in the language of the experiment, the answer was considered not valid. Responses such as variations of “I don’t know” were also marked invalid. The percentage of invalid responses was 1.23% for the French-dominant group, 2.03% for the Spanish-dominant group, 4.33% for the Spanish–French bilingual group, and 4.61% for the English–French bilingual group. Note that these responses were included in the computation of cloze probability and entropy, such that the resulting distributions reflect the full range of observed responses. All processed answers are available in the OSF repository (osf.io/k3ptv/).

## Analysis 1: Effect of language

### Objective

In Analysis 1, we tested whether sentence constraint is influenced by language. To this end, we compared sentence constraint between French-dominant participants completing French sentence contexts and Spanish-dominant participants completing Spanish sentence contexts.

### Results

All analyses were based on response entropy as a measure of sentence constraint and replicated using the more popular measure of cloze probability. Here, we report full results based on response entropy only. The corresponding cloze probability plots are provided in Appendix B, and both sets of complete analyses are available in the OSF repository (osf.io/k3ptv/).

**Fig. 2:**
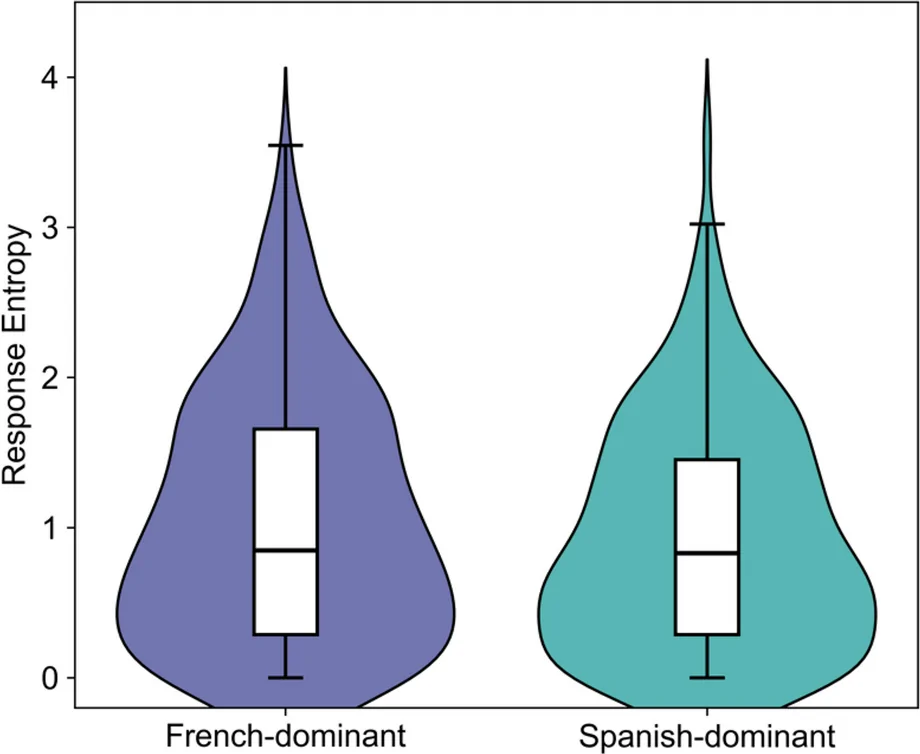
Response entropy distributions for French- and Spanish-dominant speaker groups

Response entropy was 1.05 on average in French (*SD* = 0.82, range = 0–3.55) and 0.93 in Spanish (*SD* = 0.75, range = 0–3.65; see Fig. 2). We collected 20 responses per sentence context in each language group, yielding a maximum response entropy value of 4.46, corresponding to a hypothetical case in which all participants provide unique answers. We ran a Mann–Whitney *U* test to compare averages across groups because the data were not normally distributed. The difference in average response entropy across groups was not significant (*U* = 50126.0, *p* =.076, *d* = – 0.16).

**Fig. 3:**
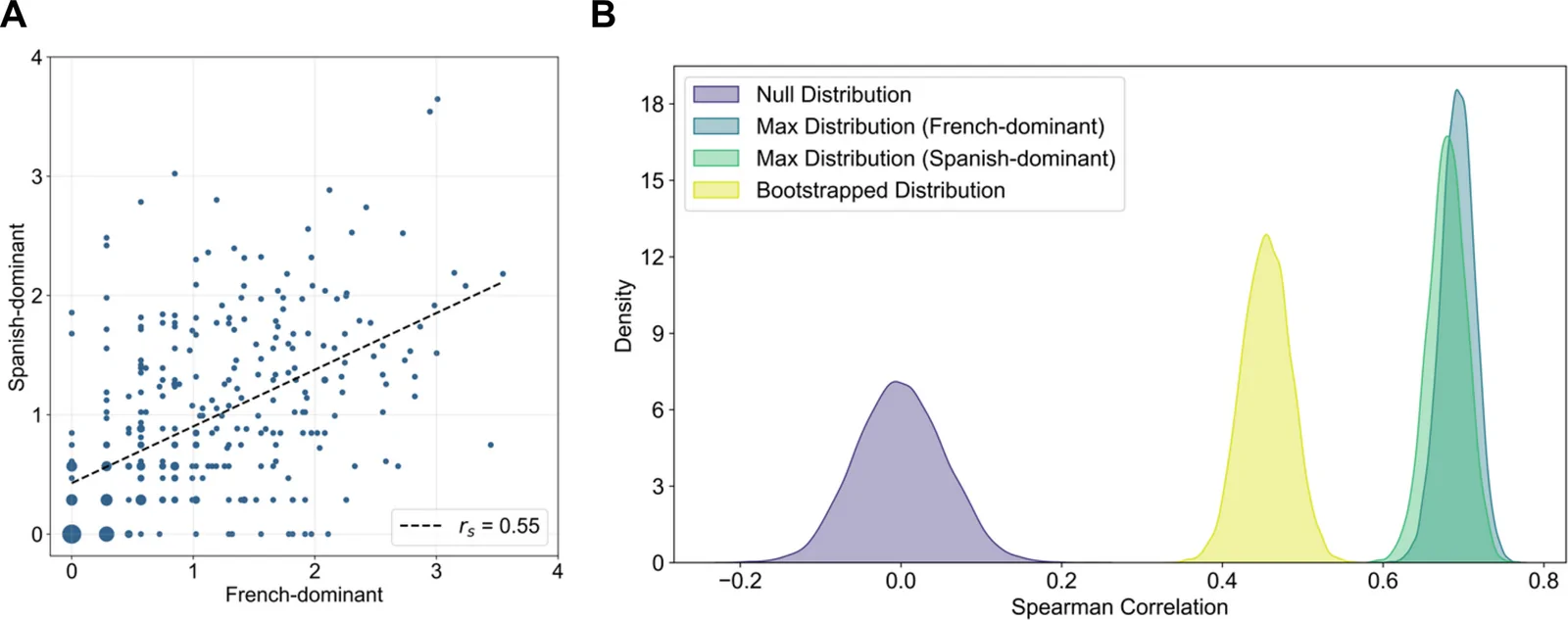
Response entropy alignment between French- and Spanish-dominant speaker groups

We additionally computed the Spearman correlation to estimate how aligned the groups were in terms of response entropy for each item. The observed correlation was *r*_*S*_ =.55, represented in a scatter plot in Fig. 3A. Within-group consistency was estimated by randomly splitting each group into two halves and correlating response entropy scores across splits, yielding correlations of *r*_*S*_ =.67 for the French-dominant group and *r*_*S*_ =.67 for the Spanish-dominant group.

To further interpret the observed correlation, we used advanced analyses to develop correlation benchmarks that were tailored to our data. First, we randomly sampled half of the 60 participants of each group, processed their answers, and used bootstrapping (10,000 iterations) to generate a distribution of observed correlations between the groups’ response entropy scores. To simulate null alignment between the groups, we performed a permutation test on the same sampled participants where we shuffled the data pairs 10,000 times and obtained the corresponding correlation each time^1^. This provided a distribution of correlations between two sets of response entropy scores that are expected to have null alignment. Deviation of the bootstrapped distribution from this null distribution was taken as evidence of non-random alignment between the groups. To simulate maximum alignment, we randomly split all 60 participants into two subgroups of 30 each, calculated response entropy scores for each subgroup, and then obtained the correlation between the two sets of scores. This procedure was repeated 10,000 times per group, yielding a distribution of correlations that reflects the group’s internal consistency. We used the proximity of the bootstrapped distribution to these maximum-alignment benchmarks to contextualize the observed correlation relative to within-group consistency, since we assume that any difference between the response entropy scores of two distinct groups should be at least as large as the difference observed within a single group. In other words, we hypothesized that the answers of a group of French-dominant speakers should differ more from those of Spanish-dominant speakers than from another group of French-dominant speakers. We based statistical comparisons between distributions on mean differences and their 95% confidence intervals; intervals not including zero indicated reliable differences between distributions.

Figure 3B represents the density distributions of all bootstrapped, null, and maximum correlation distributions of response entropy scores. The observed correlation distribution was reliably higher than the null correlation distribution, with a mean difference of – 0.45 (95% CI [– 0.456, – 0.453]), indicating strong deviation from chance-level alignment between groups. However, the observed correlation distribution remained lower than the maximum alignment benchmarks for both the French-dominant group (mean difference = – 0.24, 95% CI [– 0.238, – 0.237]) and the Spanish-dominant group (mean difference = – 0.23, 95% CI [– 0.225, – 0.224]).

### Interim discussion

In Analysis 1, we compared lexical sentence constraint as measured by response entropy between French- and Spanish-dominant speakers completing the same set of sentence contexts in their respective language. While entropy was low on average and comparable across both groups, the item-by-item alignment was moderate: although the correlation was far from null, it fell short of what we would expect if the two groups were similar and alignment was maximal.

In what follows, we present Analysis 2 before integrating the findings with those of Analysis 1 in the General discussion.

## Analysis 2: Effect of language dominance

### Objective

In Analysis 2, we tested whether language dominance influenced sentence constraint. To this end, we compared sentence constraint between French-dominant participants and two bilingual groups (Spanish–French and English–French) completing French sentence contexts. We further explored whether sentence constraint differed between the two groups of non-dominant French speakers with different dominant languages.

### Results

As in Analysis 1, all analyses were based on response entropy and replicated using cloze probability as a measure of sentence constraint. The corresponding cloze probability plots are provided in Appendix B, and both sets of complete analyses are available in the OSF repository (osf.io/k3ptv/).

**Fig. 4:**
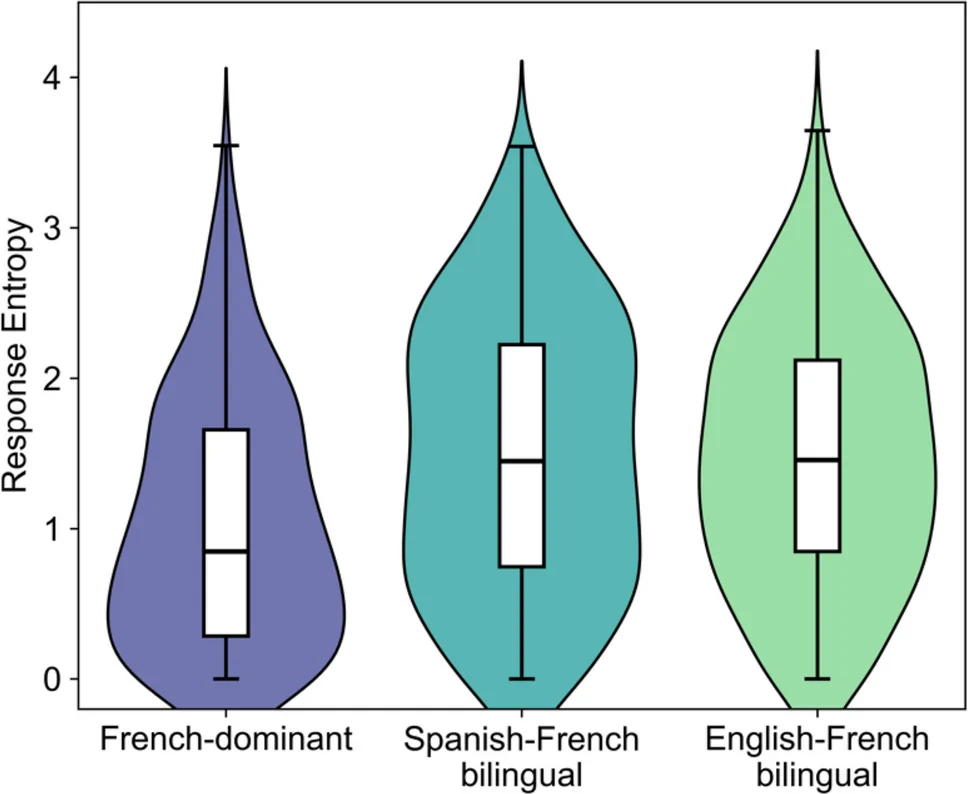
Response entropy distributions for French-dominant, Spanish–French and English–French bilingual groups

Response entropy was on average 1.05 in the French-dominant group (*SD* = 0.82, range = 0–3.55), 1.50 in the Spanish–French bilingual group (*SD* = 0.91, range = 0–3.54), and 1.47 in the English–French bilingual group (*SD* = 0.85, range = 0–3.65; see Fig. 4). We ran a Kruskal–Wallis *H* test to compare the averages across groups since the data was not normally distributed. There was a significant difference across the groups in response entropy (*H* = 53.75, *p* <.001). Post hoc comparisons using the Mann–Whitney *U* test revealed that there was a significant difference between the dominant and bilingual groups, i.e., between the French-dominant and Spanish–French bilingual groups (*U* = 38976.5, *p* <.001) and between the French-dominant and English–French bilingual groups (*U* = 38875.0, *p* <.001). However, Spanish–French and English–French bilingual groups did not show a significant difference (*U* = 55207.0, *p* =.757). Within-group consistency was *r*_*S*_ =.67 for the French-dominant group, *r*_*S*_ =.71 for the Spanish–French dominant group, and *r*_*S*_ =.69 for the English–French dominant group.

**Fig. 5:**
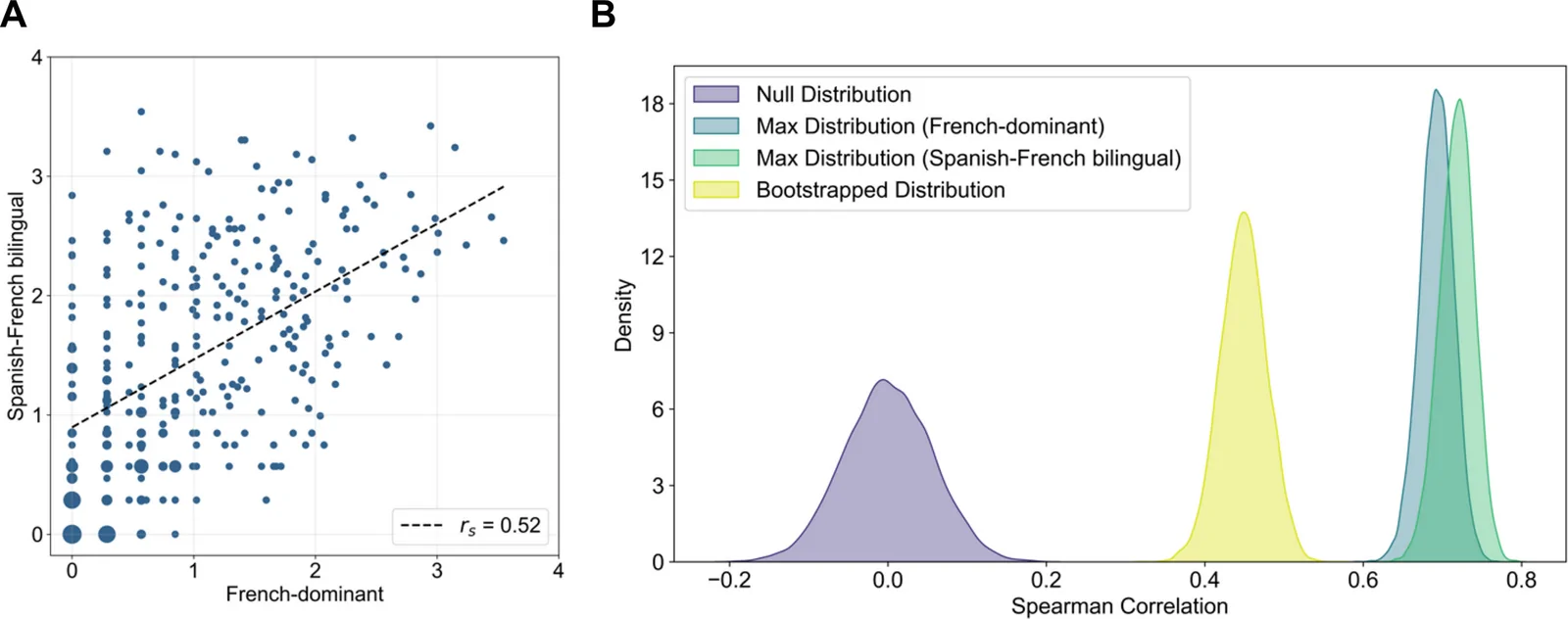
Response entropy alignment between French-dominant and Spanish–French bilingual speaker groups

Figure 5 compares the French-dominant and Spanish–French bilingual groups. The observed Spearman correlation was *r*_*S*_ =.52 (Fig. 5A). When comparing the correlation distributions (Fig. 5B), the observed correlation was reliably higher than the null correlation distribution, with a mean difference of – 0.45 (95% CI [– 0.450, – 0.448]). The observed correlation distribution was substantially lower than the maximum alignment benchmarks for both the French-dominant group (mean difference = – 0.24, 95% CI [– 0.244, – 0.243]) and the Spanish–French bilingual group (mean difference = – 0.27, 95% CI [– 0.270, – 0.268]).

**Fig. 6:**
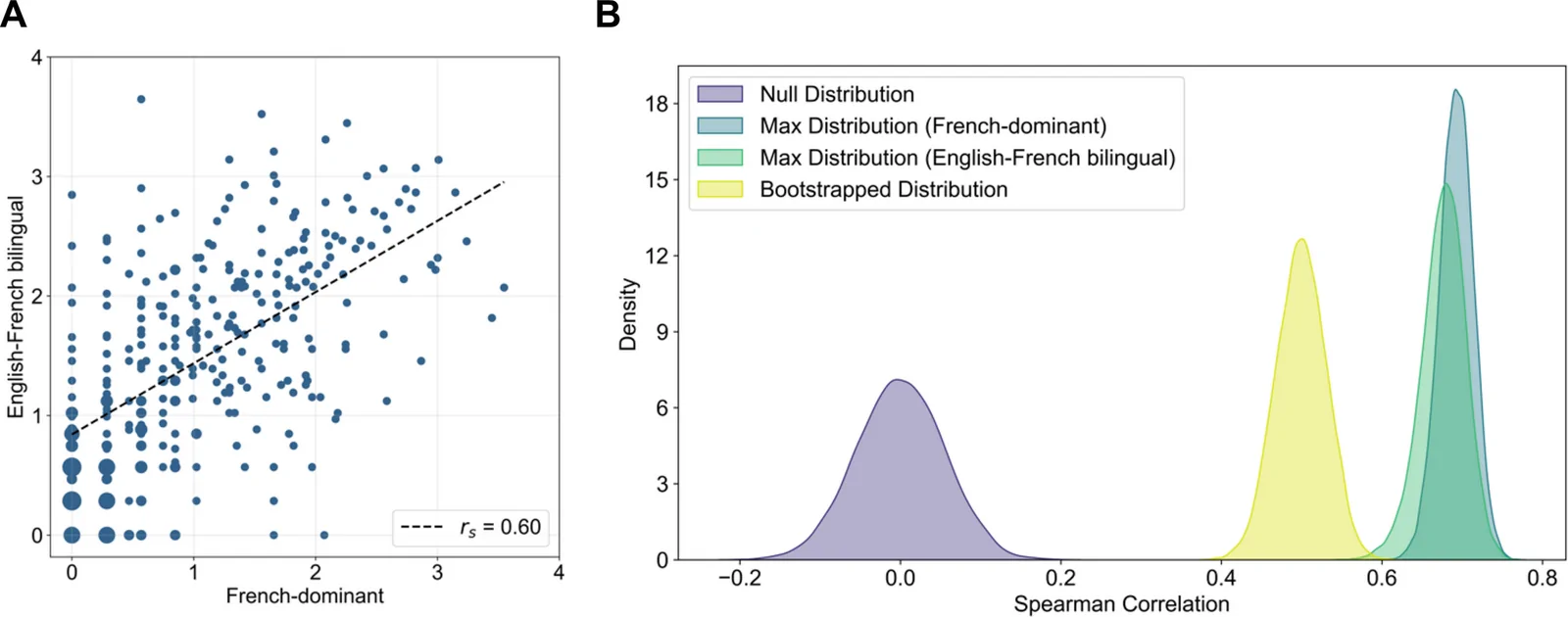
Response entropy alignment between French-dominant and English–French bilingual speaker groups

Figure 6 compares the French-dominant and English–French bilingual groups. The observed Spearman correlation was *r*_*S*_ =.60 (Fig. 6A). When comparing the correlation distributions (Fig. 6B), the observed correlation was reliably higher than the null correlation distribution, with a mean difference of – 0.50 (95% CI [– 0.501, – 0.498]). The observed correlation distribution was substantially lower than the maximum alignment benchmarks for both the French-dominant group (mean difference = – 0.19, 95% CI [– 0.194, – 0.193]) and the English–French bilingual group (mean difference = – 0.18, 95% CI [– 0.178, – 0.176]).

**Fig. 7:**
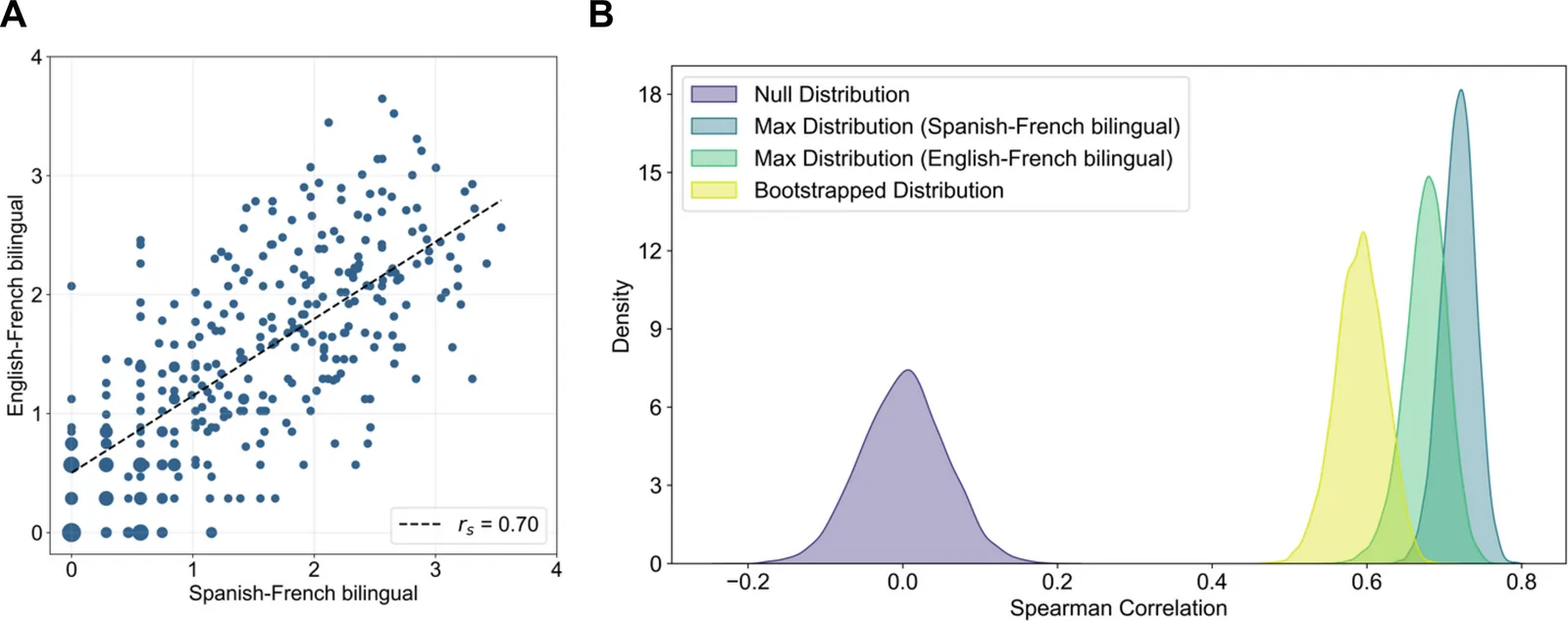
Response entropy alignment between Spanish–French and English–French bilingual speaker groups

In turn, Fig. 7 compares the Spanish–French and English–French bilingual groups. The observed Spearman correlation was *r*_*S*_ =.70 (Fig. 7A). When comparing the correlation distributions (Fig. 7B), the observed correlation was reliably higher than the null correlation distribution, with a mean difference of – 0.59 (95% CI [– 0.591, – 0.589]). The observed correlation distribution was lower than the maximum alignment benchmarks for both the Spanish–French bilingual group (mean difference = – 0.13, 95% CI [– 0.128, – 0.126]) and the English–French bilingual group (mean difference = – 0.08, 95% CI [– 0.086, – 0.084]). Nevertheless, this comparison showed the smallest deviation from the maximum alignment benchmark across all analyses.

### Interim discussion

In Analysis 2, we compared lexical sentence constraint as response entropy across different groups of dominant and non-dominant speakers of French. On average, response entropy was higher for non-dominant speakers (i.e., Spanish–French and English–French bilingual groups), indicating that sentences were less constraining for them as compared to French-dominant speakers. Item-by-item alignment between dominant and non-dominant speakers was relatively moderate considering the maximum agreement benchmarks. However, the two non-dominant groups did not significantly differ in average response entropy, and their item-by-item alignment was closest to the levels expected if the groups belonged to the same population (i.e., maximum alignment).

## General discussion

In this paper, we investigated whether lexical sentence constraint, measured by a cloze task, is influenced by language and by language dominance. In Analysis 1, we tested the influence of language by comparing sentence constraint in French- and Spanish-dominant speakers completing translations of the same sentences. In Analysis 2, we tested the influence of language dominance as well as the effect of the most dominant language when completing sentences in the non-dominant language, by comparing sentence constraint across French-dominant, Spanish–French, and English–French bilingual speakers completing French sentences. We also made all materials publicly available, providing a large, cross-linguistic database of cloze test norms across populations that may serve as a valuable resource for researchers investigating language prediction.

The results of Analysis 1 suggest that language influences the specific expectations elicited by a sentence context. In fact, item-by-item alignment between the two language groups was only moderate, even though levels of sentence constraint across languages were comparable on average. In other words, each sentence did not consistently elicit the same level of lexical constraint across the two language groups. Importantly, this moderate alignment is observed between French and Spanish, two closely related Romance languages that share substantial lexical, morphological, and cultural overlap. This suggests that the effect of language on sentence-level predictions may be even larger for more distant language pairs that differ more considerably in their linguistic and cultural characteristics (e.g., Romance and East Asian languages). Furthermore, we cannot attribute all variability to the languages involved, as our group comparison inevitably includes other factors such as cultural and social differences, despite the relative closeness of the two languages at stage. This is highlighted in sentences such as ‘The elderly woman bought potatoes and eggs because she wanted to prepare…’ (Appendix A, item 316), which was highly constraining for Spanish-dominant speakers towards “tortilla” (‘omelette’; 90% of responses), but less constraining for French-dominant speakers (40%). This group difference in answer probabilities likely reflects culturally specific associations rather than purely linguistic ones, as the sentence taps into culturally salient knowledge in Spain (where dishes like ‘tortilla,’ made of potatoes and eggs, are common and iconic) but less so in France. Therefore, we believe that the cultural iconicity and social relevance may also impact cloze test answers beyond language per se. Along this line, we can speculate that sentence constraint would differ between French-dominant speakers from France, Quebec, and Senegal, for instance, despite presenting them with the exact same sentences in the same language. This finding has important methodological implications: translating cloze materials from one language to another introduces variability in participants’ responses, even if the sentences are strict and exact translations. Accordingly, it seems that the most reliable approach remains pre-testing the sentences with the population in the target language and the specific linguistic community to be included in the main experiment. Moreover, when adapting sentence contexts to be used in another language, researchers should ensure that translations preserve the same level of specificity and cultural relevance.

The results of Analysis 2 first indicate that language dominance influences sentence constraint, since it averaged higher for dominant speakers compared to two groups of non-dominant speakers, while item-by-item alignment across dominant and non-dominant groups remained moderate. The observed differences likely reflect a combination of language dominance and proficiency effects in the target language. In the frame of experimental psycholinguistics, sentence contexts being less constraining in a less-dominant language aligns with the general intuition that language dominance and proficiency will impact cloze test responses and therefore sentence constraint. This assumption was empirically confirmed in our study, where we observed higher response entropy in Spanish–French and English–French bilingual groups when they completed sentences in their non-dominant language (French). Notably, our work builds upon the general increase in uncertainty in non-dominant groups by demonstrating misalignment between the groups’ ratings on sentence constraint. On the practical side, this implies that using cloze norms derived from dominant speakers with non-dominant populations will inevitably introduce some error, at least with respect to lexical sentence constraint. In this case, the best option remains to pre-test the materials with a population of similar language dominance level, as done in research on second language prediction (e.g., Foucart et al., 2014; Ito et al., 2018; Martin et al., 2013).

The results of Analysis 2 also suggest that lexical sentence constraint in a non-dominant language is not very strongly modulated by the most dominant language of the speakers. Two groups of non-dominant speakers of French, with similar proficiency in French yet different dominant languages (Spanish and English), showed high alignment in their sentence completions, approaching levels expected under maximum alignment. This outcome suggests that the level of language dominance in the language of the sentences plays a key role in shaping cloze test responses, and that norms obtained from one non-dominant speaker group may be applicable to another non-dominant speaker group with similar proficiency. However, these conclusions are based on a specific comparison between Spanish–French and English–French bilingual speakers when completing sentences in French. Despite differences in their most dominant language, both groups arguably share cultural attributes and linguistic and social background. Therefore, the relatively high alignment in their answers may not be fully attributable to the dominance level of the language in the experiment. In fact, cloze norms measured in Spanish–French bilingual speakers might be adequate for English–French bilinguals, but not necessarily for Mandarin Chinese–French bilingual speakers, for instance, given the greater cultural cross-linguistic distance. Further research should explore whether alignment in cloze test responses extends to other language combinations.

Caution should be taken when extracting conclusions from these results, especially those that influence the methodology to be used in future norming procedures of language prediction studies. The results presented here only refer to lexical prediction within a very specific set of sentences that were constraint towards a noun phrase, tested with four specific language groups. The generalizability of these results is tied to these specific conditions and populations and does not guarantee that differences and/or similarities observed across group pairs will persist when investigating another linguistic unit, using different sentences, or testing different language groups. All sentences used in our study were intended to be highly constraining toward noun phrase completions, which may have biased participants’ expectations and limited the range of possible responses. We believe that exploring a broader range of sentence types with varying degrees of constraint and different grammatical targets would offer a more comprehensive understanding of sentence predictability. We encourage future studies to further investigate the differences highlighted in our experiments as language and language dominance to fully ascertain their role in shaping cloze test answers.

An additional limitation concerns the demographic differences between groups, particularly gender distribution and age. Gender distribution differed significantly across groups, but no established theoretical or empirical evidence links gender to systematic differences in cloze task answers or sentence constraint for the type of neutral sentence materials used in the present study. Additionally, although group differences in age reached statistical significance in some comparisons, they were relatively small in magnitude (e.g., approximately four years on average between groups) and are unlikely to account for the observed effects. Previous research on sentence processing and cloze task answers suggests that sentence constraint is generally stable across adulthood within the age ranges tested here (Lahar et al., 2004). Nevertheless, we cannot fully rule out that demographic factors contributed in part to group differences, and future studies with more tightly matched samples would help further strengthen these conclusions.

Despite this limitation, these results offer valuable practical implications, particularly for recent trends in research on bilingual group comparisons (Kutlu & Hayes-Harb, 2023; Rothman et al., 2023). For instance, when comparing prediction abilities between L1 and L2 speakers of a language, cloze norming with each specific group may still be necessary. Additionally, when comparing prediction abilities of bilingual speakers in their L1 and L2, cloze norming in each specific language might also be important since assuming cross-linguistic similarities in sentence constraint would be incorrect, as our findings suggest. However, when comparing the prediction abilities of two groups of non-dominant speakers featuring different dominant languages, one common cloze test may be sufficient without introducing substantial error. These findings are highly relevant for the efficiency of language prediction research with diverse language groups.

Our results were replicated using both traditional and recent measures of sentence constraint: cloze probability and response entropy. Cloze probability has long been the standard measure of sentence constraint, while response entropy captures variability across the full distribution of responses rather than focusing on the most frequent continuation. In our data, both measures led to the same conclusions and showed similar patterns of alignment across groups, suggesting that the choice between them may not substantially impact results in practice. The correlation between the variables in each group was also extremely high, *r*_*s*_ > 0.96 (see Appendix C). Nevertheless, although response entropy captures variation beyond the most frequent response, it can at times be overly sensitive. Because the entropy score increases with the number of unique responses, certain distributions can yield counterintuitive results. For example, a 50-50 response distribution would produce a lower entropy score (entropy = 0.05) than a 70-10-10-10 distribution (entropy = 1.36), even though the former arguably reflects greater uncertainty. This example suggests that the currently used formula for response entropy has limitations when applied to cloze test answers and their interpretability. It is possible that a modified version of response entropy that adjusts the score based on the number of participants or normalizes it accordingly could reflect sentence constraint and predictive uncertainty more accurately. For now, given the present results, we conclude that both cloze probability and response entropy remain informative and useful tools for sentence norming procedures.

Another finding with strong practical implications is that consistency in response entropy or cloze probability scores is not perfect in any group, indicating a large degree of internal variability. On average, the maximum Spearman correlation found between two halves of the same group was *r*_*S*_ =.70. This suggests that cloze test results can vary even within the same population, and caution is needed when extrapolating pre-test results to the experimental population. In other words, while pre-testing provides valuable information about the materials, the exact sentence constraint scores from a cloze pre-test are unlikely to perfectly match those of the main test sample. This inherent variability highlights the probabilistic nature of sentence prediction and the importance of interpreting cloze norms as estimates rather than fixed standards.

Some proposals have attempted to explain how subjects perform cloze tasks, which becomes particularly relevant considering our findings that responses are shaped by both language and language dominance. For instance, Smith & Levy (2011) assume that people have subjective probability distributions and that their cloze answers are simply the result of a one-time sampling from that distribution. In that framework, the results of the present investigation may be tapping into cross-linguistic differences (Analysis 1), and dominance-related differences (Analysis 2) in the size and spread of such probability distributions across participants. In contrast, Staub et al. (2015) propose that different competitors in the probability distributions are activated, and that people produce the first word that reaches a certain threshold in the level of activation. Since we have found that sentence constraint is lower in non-dominant than in dominant language groups (Analysis 2), it may be that more candidates are activated when providing the continuation of a sentence in a non-dominant language, or that the threshold is simply lower in the non-dominant language. Future research should explore those alternative frameworks to clarify the cognitive mechanisms underlying the observed effects of language and language dominance on cloze task responses.

Finally, the results from our two experiments invite reflection on the use of surprisal estimates generated by large language models (LLMs) as measures of sentence constraint. Recent studies show that surprisal estimates from LLMs can successfully predict well-established effects of language prediction, such as ERP N400 amplitudes and eye movement patterns during reading (de Varda & Marelli, 2022; de Varda et al., 2024; Frank et al., 2015; Krieger et al., 2024; Lopes Rego et al., 2024; Michaelov et al., 2023; Michaelov et al., 2024). However, most of this evidence comes from English or other Germanic languages, which constitute the main language data on which LLMs are trained (de Varda & Marelli, 2022; Navigli et al., 2023). Moreover, LLMs have been found to have strong biases towards Western cultural norms and normative forms of English (Fleisig et al., 2024; Naous et al., 2023; Wang et al., 2024).

Our findings indicate that sentence constraint derived from cloze task responses is influenced by language and language dominance. Because LLMs are unlikely to capture the specific language learning trajectories and sociolinguistic profiles of non-English-dominant speakers, they may misrepresent their specific word choices in sentence continuation tasks. One practical implication is that researchers should carefully match the choice of a language model to the linguistic profile of their target population. For instance, when studying bilingual populations, bilingual language models trained on comparable language pairs (as in Arnett et al., 2025) may provide more appropriate estimates than large multilingual models. Similarly, for monolingual populations in non-English languages, monolingual models trained on the specific language may yield more representative surprisal estimates (see Chang et al., 2026). More systematic comparisons between LLM-derived surprisal estimates and human-derived cloze norms across diverse sociolinguistic groups are needed to determine under which conditions such models provide reliable estimates, and to what extent they can improve the efficiency of cloze norming procedures.

## Supplementary Information

Below is the link to the electronic supplementary material.

Supplementary file1 (DOCX 1197 KB)

## Data Availability

All data generated and analyzed during this study is accessible through the Open Science Framework (OSF) at the following repository link: https://osf.io/k3ptv/.

## Footnotes

1 This approach is similar to obtaining a *p* value for the observed correlation. However, we performed permutations instead because the data was not normally distributed, making standard *p* values inappropriate in this context. It also complements the graph and facilitates a complete comparison of the correlation distributions.

## Code availability

The code used to process and analyze the data in this study is accessible through the Open Science Framework (OSF) at the following repository link: https://osf.io/k3ptv/.

## References

[CR1] Altarriba, J., Kroll, J. F., Sholl, A., & Rayner, K. (1996). The influence of lexical and conceptual constraints on reading mixed-language sentences: Evidence from eye fixations and naming times. *Memory & Cognition,**24*(4), 477–492. 10.3758/BF03200936 8757496

[CR2] Angulo-Chavira, A. Q., Castellón-Flores, A. M., Ciria, A., & Arias-Trejo, N. (2024). Sentence-final completion norms for 2925 Mexican Spanish sentence contexts. *Behavior Research Methods,**56*, 2486–2498. 10.3758/s13428-023-02160-y PMC10991019 37407787

[CR3] Anwyl-Irvine, A. L., Massonnié, J., Flitton, A., Kirkham, N., & Evershed, J. K. (2020). Gorilla in our midst: An online behavioral experiment builder. *Behavior Research Methods,**52*(1), 388–407. 10.3758/s13428-019-01237-x PMC7005094 31016684

[CR4] Arnett, C., Chang, T. A., Michaelov, J. A., & Bergen, B. (2025). On the acquisition of shared grammatical representations in bilingual language models. In W. Che, J. Nabende, E. Shutova, & M. T. Pilehvar (Eds.), *Proceedings of the 63rd Annual Meeting of the Association for Computational Linguistics (Volume 1: Long Papers)* (pp. 20707–20726). Association for Computational Linguistics. 10.18653/v1/2025.acl-long.1010

[CR5] Block, C. K., & Baldwin, C. L. (2010). Cloze probability and completion norms for 498 sentences: Behavioral and neural validation using event-related potentials. *Behavior Research Methods,**42*(3), 665–670. 10.3758/BRM.42.3.665 20805588

[CR6] Bloom, P. A., & Fischler, I. (1980). Completion norms for 329 sentence contexts. *Memory & Cognition,**8*, 631–642. 10.3758/BF03213783 7219183

[CR7] Brunel, J., Dujardin, E., Delord, S., & Mathey, S. (2025). Emotional valence, cloze probability, and entropy: Completion norms for 403 French sentences. *Behavior Research Methods*. 10.3758/s13428-025-02604-7 39890703

[CR8] Brysbaert, M. (2013). LexTALE_FR: A fast, free, and efficient test to measure language proficiency in French. *Psychologica Belgica,**53*(1), 23–37. 10.5334/pb-53-1-23

[CR9] Chang, T. A., Arnett, C., Tu, Z., & Bergen, B. K. (2026). Goldfish: Monolingual language models for 350 languages. In S. Piperidis, N. Bel, H. van den Heuvel, N. Ide, S. Krek, & A. Toral (Eds.), *Proceedings of the Fifteenth Language Resources and Evaluation Conference* (pp. 3750-3781). ELRA Language Resources Association. 10.63317/5ceec3hhv4d5

[CR10] Collins. (n. d.). *Collins English–French Dictionary.* Collins Dictionary. Retrieved December 24, 2025, from https://www.collinsdictionary.com/us/dictionary/english-french

[CR11] de Varda, A. G., & Marelli, M. (2022). The effects of surprisal across languages: Results from native and non-native reading. *Findings of the Association for Computational Linguistics: AACL-IJCNLP* (pp. 138–144). Association for Computational Linguistics. 10.18653/v1/2022.findings-aacl.13

[CR12] de Varda, A. G., Marelli, M., & Amenta, S. (2024). Cloze probability, predictability ratings, and computational estimates for 205 English sentences, aligned with existing EEG and reading time data. *Behavior Research Methods,**56*, 5190–5213. 10.3758/s13428-023-02261-8 PMC11289024 37880511

[CR13] DeLong, K. A., Urbach, T. P., & Kutas, M. (2005). Probabilistic word pre-activation during language comprehension inferred from electrical brain activity. *Nature Neuroscience,**8*(8), 1117–1121. 10.1038/nn1504 16007080

[CR14] Federmeier, K. D., & Kutas, M. (1999). A rose by any other name: Long-term memory structure and sentence processing. *Journal of Memory and Language,**41*, 469–495. 10.1006/jmla.1999.2660

[CR15] Federmeier, K. D., McLennan, D. B., De Ochoa, E., & Kutas, M. (2002). The impact of semantic memory organization and sentence context information on spoken language processing by younger and older adults: An ERP study. *Psychophysiology,**39*(2), 133–146. 10.1017/S0048577202001373 12212662

[CR16] Fleisig, E., Smith, G., Bossi, M., Rustagi, I., Yin, X., & Klein, D. (2024*). Linguistic Bias in ChatGPT: Language Models Reinforce Dialect Discrimination.* ArXiv. 10.48550/arXiv.2406.08818

[CR17] Foucart, A., Martin, C. D., Moreno, E. M., & Costa, A. (2014). Can bilinguals see it coming? Word anticipation in L2 sentence reading. *Journal of Experimental Psychology. Learning, Memory, and Cognition,**40*(5), 1461–1469. 10.1037/a0036756 24797441

[CR18] Frade, S., Santi, A., & Raposo, A. (2024). Filling the gap: Cloze probability and sentence constraint norms for 807 European Portuguese sentences. *Behavior Research Methods,**56*, 4409–4018. 10.3758/s13428-023-02196-0 PMC11133143 37553535

[CR19] Frank, S. L. (2013). Uncertainty reduction as a measure of cognitive load in sentence comprehension. *Topics in Cognitive Science,**5*(3), 475–494. 10.1111/tops.12025 23681508

[CR20] Frank, S. L., Otten, L. J., Galli, G., & Vigliocco, G. (2015). The ERP response to the amount of information conveyed by words in sentences. *Brain and Language,**140*, 1–11. 10.1016/j.bandl.2014.10.006 25461915

[CR21] Honnibal, M., & Montani, I. (2017). *spaCy 2: Natural language understanding with Bloom embeddings, convolutional neural networks and incremental parsing.* Software Retrieved December 24, 2025, from https://spacy.io

[CR22] InterContact (2022). *Why does content expand when translated? *Retrieved December 24, 2026, from https://www.inter-contact.de/en/blog/text-length-languages

[CR23] Ito, A., Pickering, M. J., & Corley, M. (2018). Investigating the time-course of phonological prediction in native and non-native speakers of English: A visual world eye-tracking study. *Journal of Memory and Language,**98*, 1–11. 10.1016/j.jml.2017.09.002

[CR24] Izura, C., Cuetos, F., & Brysbaert, M. (2014). Lextale-Esp: A test to rapidly and efficiently assess the Spanish vocabulary size. *Psicológica,**35*(1), 49–66.

[CR25] Krieger, B., Brouwer, H., Aurnhammer, C., & Crocker, M. W. (2024). On the limits of LLM surprisal as functional explanation of ERPs. *Proceedings of the Annual Meeting of the Cognitive Science Society,**46*, 488–495. Retrieved December 24, 2025, from https://escholarship.org/uc/item/2m53k85t

[CR26] Kutlu, E., & Hayes-Harb, R. (2023). Towards a just and equitable applied psycholinguistics. *Applied Psycholinguistics,**44*(3), 293–300. 10.1017/S0142716423000280

[CR27] Lahar, C. J., Tun, P. A., & Wingfield, A. (2004). Sentence-final word completion norms for young, middle-aged, and older adults. *The Journals of Gerontology. Series B, Psychological Sciences and Social Sciences,**59*(1), P7–P10. 10.1093/geronb/59.1.p7 14722333

[CR28] Lemhöfer, K., & Broersma, M. (2012). Introducing LexTALE: A quick and valid lexical test for advanced learners of English. *Behavior Research Methods,**44*, 325–343. 10.3758/s13428-011-0146-0 PMC3356522 21898159

[CR29] Levy, R., Fedorenko, E., & Gibson, E. (2013). The syntactic complexity of Russian relative clauses. *Journal of Memory and Language,**69*, 461–495. 10.1016/j.jml.2012.10.005 PMC3975271 24711687

[CR30] Linzen, T., & Jaeger, T. F. (2016). Uncertainty and expectation in sentence processing: Evidence from subcategorization distributions. *Cognitive Science,**40*(6), 1382–1411. 10.1111/cogs.12274 26286681

[CR31] Lopes Rego, A. T., Snell, J., & Meeter, M. (2024). Language models outperform cloze predictability in a cognitive model of reading. *PLoS Computational Biology,**20*(9), e1012117. 10.1371/journal.pcbi.1012117 PMC11458034 39321153

[CR32] Lowder, M. W., Choi, W., Ferreira, F., & Henderson, J. M. (2018). Lexical predictability during natural reading: Effects of surprisal and entropy reduction. *Cognitive Science,**42*(S4), 1166–1183. 10.1111/cogs.12597 PMC5988918 29442360

[CR33] Marian, V., Bartolotti, J., Chabal, S., & Shook, A. (2012). CLEARPOND: Cross-linguistic easy-access resource for phonological and orthographic neighborhood densities. *PLoS ONE,**7*(8), e43230. 10.1371/journal.pone.0043230 PMC3423352 22916227

[CR34] Martin, C. D., Thierry, G., Kuipers, J., Boutonnet, B., Foucart, A., & Costa, A. (2013). Bilinguals reading in their second language do not predict upcoming words as native readers do. *Journal of Memory and Language,**69*(4), 574–588. 10.1016/J.JML.2013.08.001

[CR35] Michaelov, J. A., Bardolph, M. D., Van Petten, C. K., Bergen, B. K., & Coulson, S. (2024). Strong prediction: Language model surprisal explains multiple N400 effects. *Neurobiology of Language,**5*(1), 107–135. 10.1162/nol_a_00105 PMC11025652 38645623

[CR36] Michaelov, J. A., Coulson, S., & Bergen, B. K. (2023). So cloze yet so far: N400 amplitude is better predicted by distributional information than human predictability judgements. *IEEE Transactions on Cognitive and Developmental Systems,**15*(3), 1033–1042. 10.1109/TCDS.2022.3176783

[CR37] Naous, T., Ryan, M. J., Ritter, A., & Xu, W. (2023). *Having Beer after Prayer?**Measuring Cultural Bias in Large Language Models.* ArXiv. 10.48550/arXiv.2305.14456

[CR38] Navigli, R., Conia, S., & Ross, B. (2023). Biases in large language models: Origins, inventory, and discussion. *Journal of Data and Information Quality,**15*(2), 1–21. 10.1145/3597307

[CR39] Peelle, J. E., Miller, R. L., Rogers, C. S., Spehar, B., Sommers, M. S., & Van Engen, K. J. (2020). Completion norms for 3085 English sentence contexts. *Behavior Research Methods,**52*, 1795–1799. 10.3758/s13428-020-01351-1 PMC7406521 31993960

[CR40] Real Academia Española. (2024). *Diccionario de la lengua española* (23rd ed., version 23.8). Retrieved December 24, 2025, from https://dle.rae.es/

[CR41] Rossi, N. F., Fernandes, C., Moreira, C. S., Giacheti, C. M., Sichieri, B. B., Pinheiro, A. P., & Sampaio, A. (2020). Sentence contexts and cloze probabilities for Brazilian Portuguese children and adolescents. *PLoS One,**15*(7), e0236388. 10.1371/journal.pone.0236388 PMC7392299 32730342

[CR42] Rothman, J., Bayram, F., DeLuca, V., Di Pisa, G., Duñabeitia, J. A., Gharibi, K., & Wulff, S. (2023). Monolingual comparative normativity in bilingualism research is out of “control”: Arguments and alternatives. *Applied Psycholinguistics,**44*(3), 316–329. 10.1017/S0142716422000315

[CR43] Smith, N., & Levy, R. (2011). Cloze but no cigar: The complex relationship between cloze, corpus, and subjective probabilities in language processing. *Proceedings of the Annual Meeting of the Cognitive Science Society,**33*, 1637–1642. Retrieved December 24, 2025, from https://escholarship.org/uc/item/69s3541f

[CR44] Staub, A., Grant, M., Astheimer, L., & Cohen, A. (2015). The influence of cloze probability and item constraint on cloze task response time. *Journal of Memory and Language,**85*, 1–17. 10.1016/j.jml.2015.02.004

[CR45] Taylor, W. L. (1953). “Cloze procedure”: A new tool for measuring readability. *Journalism Bulletin,**30*, 415–433.

[CR46] Wang, W., Jiao, W., Huang, J., Dai, R., Huang, J., Tu, Z., & Lyu, M. (2024). Not All Countries Celebrate Thanksgiving: On the Cultural Dominance in Large Language Models. *Proceedings of the 62nd Annual Meeting of the Association for Computational Linguistics (Volume 1: Long Papers)* (pp. 6349–6384). Association for Computational Linguistics. 10.18653/v1/2024.acl-long.345

[CR47] Whitley, A., Naylor, G., & Hadley, L. V. (2024). Used to be a dime, now it’s a dollar: Revised speech perception in noise keyword predictability revisited 40 years on. *Journal of Speech, Language, and Hearing Research,**67*(4), 1229–1242. 10.1044/2024_JSLHR-23-00615 PMC11005954 38563688

[CR48] Wicha, N. Y. Y., Moreno, E. M., & Kutas, M. (2003). Expecting gender: An event-related brain potential study on the role of grammatical gender in comprehending a line drawing within a written sentence in Spanish. *Cortex,**39*(3), 483–508. 10.1016/S0010-9452(08)70260-0 PMC3392191 12870823

[CR49] Wlotko, E. W., Federmeier, K. D., & Kutas, M. (2012). To predict or not to predict: Age-related differences in the use of sentential context. *Psychology and Aging,**27*(4), 975–988. 10.1037/a0029206 PMC3685629 22775363

