# Supplementary material for: Not that cloze: Sentence constraint is influenced by language and language dominance
Source: Behav Res Methods. 2026 Oct 14;58(11):317. doi: 10.3758/s13428-026-03140-8 (PMC13669023; doi:10.3758/s13428-026-03140-8)
Supplement: Supplementary file 1 [file 13428_2026_3140_MOESM1_ESM.docx]

**Appendix A**

**Sentences and Cloze Task Answer Distributions for Four Language Groups**

| Item | Sentence in English | Sentence in French | Sentence in Spanish | Answer distribution for French-dominant speakers to French sentence | Answer distribution for Spanish-dominant speakers to Spanish sentence | Answer distribution for Spanish-dominant speakers to French sentence | Answer distribution for English-dominant speakers to French sentence |
| --- | --- | --- | --- | --- | --- | --- | --- |
| 1 | On farms, to get milk, it is necessary to milk… | Dans les fermes, pour obtenir du lait, il faut traire… | En las granjas, para conseguir leche, es necesario ordeñar… | vache: 100% | vaca: 100% | vache: 95%, mamelle: 5% | vache: 95%, lait: 5% |
| 2 | She spent the whole night studying because the next day she had… | Elle a passé toute la nuit à étudier, car le lendemain elle avait… | Ella pasó toda la noche estudiando porque al día siguiente tenía… | examen: 85%, contrôle: 5%, devoir: 5%, école: 5% | examen: 95%, oposición: 5% | examen: 95%, NV: 5% | examen: 90%, essai: 5%, travail: 5% |
| 3 | Nuria/Léa didn’t want her friends to leave ashes on the table when they were smoking, so she brought them… | Léa ne voulait pas que ses amis laissent des cendres sur la table quand ils fumaient, elle leur a donc apporté… | Nuria no quería que sus amigos dejaran ceniza en la mesa cuando fumaban, así que les trajo… | cendrier: 100% | cenicero: 100% | cendrier: 65%, NV: 10%, maison: 5%, other(s): 20% | cendrier: 85%, assiette: 5%, cigarette: 5%, plateau: 5% |
| 4 | The boy was very tired after running for hours and winning… | Le garçon était très fatigué après avoir couru pendant des heures et gagné… | El chico estaba muy cansado después de correr durante horas y ganar… | course: 40%, marathon: 40%, match: 10%, other(s): 10% | maratón: 50%, carrera: 35%, casa: 5%, other(s): 10% | course: 50%, marathon: 25%, jeu: 5%, other(s): 20% | course: 40%, marathon: 20%, médaille: 15%, other(s): 25% |
| 5 | Ever since she tried Japanese food and learned to eat using chopsticks, she keeps saying she loves… | Depuis qu'elle a goûté à la cuisine japonaise et a appris à manger avec des baguettes, elle ne cesse de répéter qu'elle adore… | Desde que probó la comida de Japón y aprendió a comer con palillos, ella no deja de repetir que le encanta… | sushi: 45%, NV: 15%, cuisine: 10%, other(s): 30% | sushi: 60%, NV: 20%, asia: 5%, other(s): 15% | sushi: 70%, NV: 5%, baguette: 5%, other(s): 20% | cuisine: 35%, sushi: 25%, nouille: 15%, other(s): 25% |
| 6 | Her water broke while they were traveling and, since they didn’t know the area, they had to look for… | Elle a perdu les eaux alors qu'ils étaient en voyage, et comme ils ne connaissaient pas la région, ils ont dû chercher… | Rompió aguas mientras estaban de viaje, y como no conocían la zona, tuvieron que buscar… | hôpital: 75%, centre: 5%, femme: 5%, other(s): 15% | hospital: 85%, taxi: 10%, centro: 5% | hôpital: 25%, carte: 15%, NV: 10%, other(s): 50% | hôpital: 75%, carte: 10%, maternité: 5%, other(s): 10% |
| 7 | For breakfast, she always makes some toast and uses a knife to spread butter and… | Pour le petit-déjeuner, elle se prépare toujours des tartines et utilise un couteau pour étaler le beurre et… | Para desayunar, ella siempre se hace unas tostadas y usa un cuchillo para untar la mantequilla y… | confiture: 85%, miel: 5%, nutella: 5%, pâté: 5% | mermelada: 90%, miel: 5%, pan: 5% | confiture: 70%, marmelade: 10%, pain: 10%, other(s): 10% | confiture: 75%, pain: 10%, cuillère: 5%, other(s): 10% |
| 8 | She always dresses the salad herself, because she doesn’t like it when it tastes sour with… | Elle assaisonne toujours la salade elle-même, car elle n'aime pas qu'elle soit aigre avec… | Siempre se aliña ella misma la ensalada, porque no le gusta cuando queda agria con… | vinaigre: 55%, sauce: 20%, vinaigrette: 10%, other(s): 15% | vinagre: 80%, aliño: 10%, escarola: 5%, sal: 5% | vinaigre: 70%, citron: 15%, douche: 5%, other(s): 10% | vinaigre: 55%, NV: 10%, sel: 10%, other(s): 25% |
| 9 | She really likes roller coasters, and she dreams of going to… | Il aime beaucoup les montagnes russes et rêve d'aller dans… | Le gustan mucho las montañas rusas, y sueña con ir a… | parc: 70%, huit: 10%, NV: 5%, other(s): 15% | parque: 60%, portaventura: 10%, disneyland: 10%, other(s): 20% | parc: 45%, NV: 15%, alpes: 5%, other(s): 35% | NV: 20%, parc: 20%, alpes: 5%, other(s): 55% |
| 10 | To complete the exam, she was only allowed to bring an eraser and… | Pour passer l'examen, elle n'avait droit qu'à une gomme et… | Para completar el examen, a ella solo le dejaban llevar una goma y… | crayon: 90%, stylo: 10% | lápiz: 80%, lapicero: 15%, bolígrafo: 5% | crayon: 60%, stylo: 35%, calculatrice: 5% | crayon: 70%, stylo: 30% |
| 11 | Since her sister was acting in the play they wanted to see, she got them tickets for… | Comme sa sœur jouait dans la pièce de théâtre qu'ils voulaient voir, elle leur a obtenu des entrées pour… | Como su hermana actuaba en la obra de teatro que querían ir a ver, les consiguió entradas para… | NV: 30%, pièce: 20%, famille: 10%, other(s): 40% | NV: 25%, fila: 10%, función: 10%, other(s): 55% | NV: 20%, voir: 20%, première: 10%, other(s): 50% | NV: 50%, spectaclé: 20%, film: 5%, other(s): 25% |
| 12 | In origami, you can make amazing animal figures just with… | En origami, on peut réaliser des figures d'animaux étonnantes simplement avec… | En el origami, se pueden conseguir increíbles figuras de animales simplemente con… | papier: 65%, feuille: 25%, morceau: 5%, pliure: 5% | papel: 75%, NV: 5%, folio: 5%, other(s): 15% | papier: 85%, feuille: 10%, ombre: 5% | papier: 80%, plis: 10%, feuille: 5%, pliage: 5% |
| 13 | Her hands were dry, so she went to the store to buy herself… | Ses mains étaient très sèches, donc elle est allée au magasin acheter… | Ella tenía las manos muy secas, así que fue a la tienda a comprarse… | crème: 100% | crema: 95%, nivea: 5% | crème: 95%, NV: 5% | crème: 90%, lotion: 5%, pommade: 5% |
| 14 | The woman was tired from working since Monday, so she couldn’t wait for… | La femme était si fatiguée de travailler depuis le lundi, qu'elle ne pouvait plus attendre qu'arrive… | La mujer estaba cansada de trabajar desde el lunes, así que no podía esperar a que llegase… | week-end: 90%, vendredi: 10% | fin de semana: 75%, viernes: 15%, descanso: 5%, hora: 5% | week-end: 70%, vendredi: 15%, NV: 5%, other(s): 10% | week-end: 70%, lit: 10%, vendredi: 10%, other(s): 10% |
| 15 | She has visited almost all the major regions of Spain, but never Madrid, even though it is… | Elle avait visité presque toutes les régions importantes d'Espagne, mais jamais Madrid, qui est pourtant… | Ella había visitado casi todas las regiones importantes de España, pero nunca Madrid, y eso que es… | capitale: 85%, ville: 10%, NV: 5% | capital: 90%, NV: 5%, zona: 5% | capitale: 85%, NV: 5%, attente: 5%, visite: 5% | capitale: 65%, NV: 30%, région: 5% |
| 16 | She had already done the shopping, but she wanted fresh bread, so she stopped by… | Elle avait déjà fait les courses, mais elle voulait du pain frais, elle est donc passée par… | Ya había hecho la compra pero quería pan recién hecho, así que ella se pasó por… | boulangerie: 100% | panadería: 90%, supermercado: 5%, tienda: 5% | boulangerie: 90%, chambre: 5%, marché: 5% | boulangerie: 95%, allée: 5% |
| 17 | Lara/Lena had a bit of a headache during the party, so she asked to lower… | Lena avait un peu mal à la tête pendant la fête et a donc demandé à ce que l'on réduise… | A Lara le dolía un poco la cabeza durante la fiesta, así que pidió reducir… | volume: 40%, son: 35%, musique: 15%, other(s): 10% | volumen: 80%, música: 10%, ritmo: 5%, ruido: 5% | musique: 40%, volume: 35%, bruit: 20%, douleur: 5% | bruit: 25%, musique: 25%, volume: 25%, other(s): 25% |
| 18 | She lived near a cathedral, so during rush hour she always heard… | Elle vivait près d'une cathédrale et donc, à chaque heure pile, elle entendait toujours… | Ella vivía cerca de una catedral, así que, en hora punta, siempre escuchaba… | cloche: 85%, carillon: 5%, clocher: 5%, messe: 5% | campana: 70%, misa: 10%, campanada: 5%, other(s): 15% | cloche: 90%, NV: 5%, matinée: 5% | cloche: 85%, carillon: 5%, messe: 5%, orgue: 5% |
| 19 | She went to the store to return the dress she had bought, but they didn’t let her because she forgot to bring… | Elle est allée au magasin pour rendre la robe qu'elle avait achetée, mais on ne l'a pas laissée faire parce qu'elle avait oublié d'apporter… | Fue a la tienda a devolver el vestido que se había comprado, pero no le dejaron porque se había olvidado llevar… | ticket: 75%, robe: 10%, argent: 5%, other(s): 10% | ticket: 85%, recibo: 10%, cartera: 5% | ticket: 65%, billet: 10%, argent: 5%, other(s): 20% | reçu: 60%, ticket: 20%, NV: 5%, other(s): 15% |
| 20 | To avoid buying too much at the supermarket, she tries to write everything she needs on… | Pour ne pas acheter trop des choses au supermarché, elle essaye d'écrire tout ce dont elle a besoin sur… | Para no comprar demasiado en el supermercado, ella procura apuntar todo lo que necesita en… | liste: 85%, feuille: 10%, bout: 5% | lista: 60%, papel: 20%, hoja: 5%, other(s): 15% | liste: 45%, papier: 25%, cahier: 10%, other(s): 20% | liste: 75%, feuille: 10%, bras: 5%, other(s): 10% |
| 21 | Her phone battery was running low, so she asked if someone had… | Comme son téléphone portable n'avait plus de batterie, elle a demandé si quelqu'un avait… | Ella se estaba quedando sin batería en el móvil, así que preguntó si alguien tenía… | chargeur: 85%, batterie: 5%, câble: 5%, téléphone: 5% | cargador: 95%, batería: 5% | chargeur: 80%, NV: 10%, câble: 5%, téléphone: 5% | chargeur: 85%, téléphone: 15% |
| 22 | José/Jules read the news about the strike in… | Jules a lu la nouvelle de la grève dans… | José leyó la noticia sobre la huelga en… | journal: 85%, papier: 5%, presse: 5%, transport: 5% | periodico: 40%, fábrica: 10%, campo: 5%, other(s): 45% | journal: 60%, transport: 10%, école: 5%, other(s): 25% | journal: 75%, ville: 10%, quotidien: 5%, other(s): 10% |
| 23 | At Christmas, the tree is decorated and on top you put… | À Noël, on décore le sapin et au sommet on y met… | En Navidad, se decora el árbol y arriba se pone… | étoîle: 95%, ange: 5% | estrella: 100% | étoîle: 75%, boule: 10%, NV: 5%, other(s): 10% | étoîle: 75%, ange: 20%, babiole: 5% |
| 24 | Before going in to see the doctor, the nurse asked the patient to sit on… | Avant d'entrer dans le cabinet du médecin, l'infirmière a demandé au patient de s'assoir dans… | Antes de pasar a la consulta del doctor, la enfermera pidió al paciente que se sentase en… | salle: 90%, NV: 5%, fauteuil: 5% | sala: 60%, silla: 25%, camilla: 15% | chaise: 40%, salle: 30%, siège: 10%, other(s): 20% | salle: 50%, chaise: 35%, bureau: 5%, other(s): 10% |
| 25 | The girl can’t see anything from far away, she needs to go to the eye doctor to check… | La fille ne voit rien de loin, elle doit aller chez l'ophtalmologiste pour se faire contrôler… | La chica no ve nada de lejos, tiene que ir al oftalmólogo para revisarse… | vue: 70%, yeux: 20%, rétine: 5%, vision: 5% | vista: 90%, NV: 5%, ojo: 5% | vue: 65%, myopie: 15%, vision: 15%, capacité: 5% | yeux: 30%, lunette: 25%, vue: 15%, other(s): 30% |
| 26 | Yesterday the student went to the library to return… | Hier, l'étudiante est allée à la bibliothèque pour rendre… | Ayer la estudiante fue a la biblioteca para devolver… | livre: 95%, devoir: 5% | libro: 95%, par: 5% | livre: 90%, bouquin: 5%, nuit: 5% | livre: 90%, devoir: 5%, NV: 5% |
| 27 | In big cities, to get from one place to another, the fastest means of transportation is… | Dans les grandes villes, le moyen le plus rapide de se rendre d'un endroit à l'autre est… | En las grandes ciudades, para llegar de un lado a otro, el medio de transporte más rápido es… | métro: 55%, train: 15%, autobus: 10%, other(s): 20% | metro: 75%, autobús: 10%, coche: 5%, other(s): 10% | métro: 65%, voiture: 20%, autobus: 5%, other(s): 10% | métro: 40%, voiture: 15%, train: 10%, other(s): 35% |
| 28 | When she was quite close to the animal at the zoo, she took out her camera to take… | Lorsqu'elle a été assez proche de l'animal au zoo, elle a sorti son appareil pour faire… | Cuando estaba bastante cerca del animal en el zoo, ella sacó su cámara para hacer… | photo: 95%, selfie: 5% | foto: 90%, grabación: 5%, selfie: 5% | photo: 100% | photo: 90%, regard: 5%, vidéo: 5% |
| 29 | She had just left the house and couldn’t remember if she had locked… | Elle venait de quitter la maison et ne se souvenait plus si elle avait fermé… | Ella justo acababa de salir de casa y no recordaba si había cerrado… | porte: 85%, clé: 5%, gaz: 5%, volet: 5% | puerta: 70%, llave: 20%, grifo: 5%, ventana: 5% | porte: 100% | porte: 80%, fenêtre: 20% |
| 30 | When a cruise ship is sinking, the last one to leave is… | Lorsqu'un bateau de croisière coule, seulement en dernier lieu sort… | Cuando un crucero se está hundiendo, solo en último lugar sale… | capitaine: 70%, commandant: 5%, coque: 5%, other(s): 20% | capitán: 95%, tripulación: 5% | capitaine: 45%, NV: 20%, eau: 10%, other(s): 25% | canot: 30%, capitaine: 15%, bateau: 10%, other(s): 45% |
| 31 | It was January, it was very cold, and Tomás/Thomas had gotten lost in the mountains, but he believed he could find his way back, he just had to follow his footprints in… | C'était en janvier, il faisait très froid et il était perdu dans la montagne, mais Thomas pensait pouvoir retrouver son chemin, il lui suffisait de suivre ses traces dans… | Era enero, hacía mucho frío, y Tomás se había perdido en la montaña, pero creía que podía encontrar el camino de vuelta, solo tenía que seguir sus huellas en… | neige: 95%, sol: 5% | nieve: 65%, camino: 10%, suelo: 10%, other(s): 15% | neige: 60%, chemin: 10%, sol: 10%, other(s): 20% | neige: 80%, sol: 10%, NV: 5%, direction: 5% |
| 32 | What she hates most about getting up early is the moment when … goes off | Ce qu'elle déteste le plus dans le fait de se lever tôt, c'est le moment où sonne… | Lo que ella odia más de madrugar es el momento en que suena… | réveil: 90%, alarme: 5%, téléphone: 5% | despertador: 75%, alarma: 25% | réveil: 50%, alarme: 45%, NV: 5% | alarme: 35%, réveil: 25%, NV: 20%, other(s): 20% |
| 33 | She likes her tea very sweet, so she asked them to pass her… | Elle préfère que le thé soit très doux, elle a donc demandé qu'on lui passe… | Ella prefiere que el té esté muy dulce, por eso pidió que le pasaran… | sucre: 60%, miel: 15%, lait: 10%, other(s): 15% | azúcar: 75%, azucarero: 5%, bote: 5%, other(s): 15% | sucre: 80%, NV: 5%, eau: 5%, other(s): 10% | sucre: 45%, lait: 25%, NV: 20%, other(s): 10% |
| 34 | She put the birdcage up on a pièce of furniture, fearing that … would try to catch it | Il a placé la cage de l'oiseau en haut d'un meuble, craignant qu'il se fasse attraper par… | Subió la jaula del pájaro a un mueble temiendo que intentaría cazarlo… | chat: 80%, propriétaire: 10%, enfant: 5%, renard: 5% | gato: 95%, pájaro: 5% | chat: 85%, chien: 10%, semis: 5% | chat: 55%, chien: 20%, vent: 15%, other(s): 10% |
| 35 | Before moving into the apartment, she had to meet with the landlord to sign… | Avant d'entrer dans l'appartement, il a eu rendez-vous avec le propriétaire pour signer… | Antes de entrar al piso tuvo que quedar con el propietario para firmar… | bail: 75%, contrat: 20%, état: 5% | contrato: 95%, papel: 5% | contrat: 90%, bail: 5%, écriture: 5% | contrat: 65%, bail: 30%, document: 5% |
| 36 | He was too small to ski, so they gave him a sled to play in… | Il était très jeune et ne pouvait pas skier, ils lui ont donc offert une luge pour qu'il puisse jouer dans… | Era muy pequeño y no podía esquiar, así que le regalaron un trineo para que jugase en… | neige: 100% | nieve: 90%, parque: 5%, pista: 5% | neige: 95%, zone: 5% | neige: 95%, ski: 5% |
| 37 | She’s afraid of the dark, she never wants to stay home alone when … comes | Elle a peur du noir, elle ne veut jamais rester seule à la maison quand vient… | Le da miedo la oscuridad, nunca quiere quedarse sola en casa cuando llega… | nuit: 95%, soir: 5% | noche: 100% | nuit: 70%, père: 10%, NV: 5%, other(s): 15% | nuit: 95%, lumière: 5% |
| 38 | She really likes string instruments and rock music, so she plays… | Il aime les instruments à cordes et la musique rock, c'est pourquoi il pratique… | Le gustan mucho los instrumentos de cuerda y la música rock, así que toca… | guitare: 80%, basse: 10%, metal: 5%, musique: 5% | guitarra: 85%, bajo: 10%, grupo: 5% | guitare: 90%, groupe: 5%, soir: 5% | guitare: 90%, conservatoire: 5%, jour: 5% |
| 39 | She never knows where to carry her phone and wallet, she needs to buy herself… | Elle ne sait jamais où mettre son téléphone portable et son portefeuille, elle doit acheter… | Nunca sabe dónde llevar su móvil y su cartera, tiene que comprarse… | sac: 80%, banane: 10%, dispositif: 5%, trackeur: 5% | bolso: 95%, mochila: 5% | sac: 85%, NV: 5%, boîte: 5%, mallette: 5% | sac: 85%, NV: 5%, dispositif: 5%, porte-clé: 5% |
| 40 | To propose, he didn’t want to buy just any ring, he wanted… | Pour la demander en mariage, il ne voulait pas acheter une bague quelconque, mais… | Para pedirle matrimonio no quería comprarle un anillo cualquiera, sino… | bague: 40%, bijou: 20%, alliance: 15%, other(s): 25% | NV: 50%, diamante: 35%, alianza: 5%, other(s): 10% | NV: 40%, alliance: 10%, bague: 10%, other(s): 40% | bague: 70%, diamant: 10%, NV: 5%, other(s): 15% |
| 41 | The ice-skating time was almost over, they were going to close… | Il ne restait plus beaucoup de temps pour faire du patin à glace, ils allaient fermer… | Se estaba terminando el tiempo para patinar sobre hielo, iban a cerrar… | patinoire: 85%, NV: 5%, marché: 5%, porte: 5% | pista: 75%, NV: 10%, centro: 5%, other(s): 10% | patinoire: 45%, piste: 15%, restaurant: 10%, other(s): 30% | patinoire: 45%, NV: 10%, porte: 10%, other(s): 35% |
| 42 | It was so cold that she couldn’t wait to get home and wrap herself in… | Il faisait si froid qu'elle avait hâte de rentrer à la maison et de s'emmitoufler dans… | Hacía tanto frío que ella estaba impaciente por llegar a casa y arroparse con… | lit: 30%, couverture: 25%, couette: 20%, other(s): 25% | manta: 90%, bata: 5%, marido: 5% | lit: 35%, couverture: 25%, drap: 10%, other(s): 30% | couverture: 45%, lit: 20%, couette: 10%, other(s): 25% |
| 43 | She can’t stand the noise of the cars in the street, when she works, she always has to close… | Elle ne supporte pas le bruit des voitures dans la rue, lorsqu'elle travaille, elle doit toujours fermer… | No soporta el ruido de los coches de la calle, cuando trabaja ella siempre tiene que cerrar… | fenêtre: 100% | ventana: 100% | fenêtre: 90%, porte: 10% | fenêtre: 90%, NV: 10% |
| 44 | At the birthday party, Jorge/Théo was very surprised when his friends gave him... | Le jour de son anniversaire, Théo a été très surpris lorsque ses amis lui ont offert… | En el cumpleaños, Jorge se sorprendió mucho cuando sus amigos le dieron… | cadeau: 30%, console: 10%, billet: 5%, other(s): 55% | regalo: 85%, entrada: 10%, tarta: 5% | cadeau: 45%, gâteau: 15%, auto: 5%, other(s): 35% | cadeau: 65%, voyage: 10%, bouteille: 5%, other(s): 20% |
| 45 | When she drinks very hot coffee, she always burns... | Lorsqu'elle boit du café très chaud, elle se brûle toujours… | Cuando ella bebe café muy caliente siempre se quema… | langue: 80%, lèvre: 10%, bouche: 5%, gorge: 5% | lengua: 85%, labio: 10%, garganta: 5% | langue: 50%, NV: 15%, bouche: 15%, other(s): 20% | langue: 55%, lèvre: 20%, bouche: 10%, other(s): 15% |
| 46 | Neil Armstrong was the first man to step on... | Neil Armstrong a été le premier homme à poser le pied sur… | Neil Armstrong fue el primer hombre en pisar… | lune: 100% | luna: 100% | lune: 100% | lune: 100% |
| 47 | A few years ago, in Paris, there was a fire in... | Il y a quelques années, à Paris, un incendie s'est déclaré dans… | Hace algunos años, en París, hubo un incendio en… | cathédrale: 40%, notre dame: 20%, immeuble: 15%, other(s): 25% | notre dame: 60%, catedral: 25%, discoteca: 10%, centro: 5% | notre dame: 35%, cathédrale: 25%, bâtiment: 5%, other(s): 35% | cathédrale: 30%, notre dame: 20%, immeuble: 15%, other(s): 35% |
| 48 | Yesterday after eating some walnuts, her lips swelled, she thinks she had... | Hier, après avoir mangé des noix, ses lèvres ont gonflé, il pense qu'il a fait… | Ayer después de comer nueces se le hincharon los labios, cree que tuvo… | réaction: 50%, allergie: 45%, erreur: 5% | reacción: 75%, alergia: 20%, fiebre: 5% | allergie: 50%, réaction: 25%, NV: 5%, other(s): 20% | allergie: 45%, réaction: 45%, erreur: 5%, malade: 5% |
| 49 | He parked badly in the parking lot, so when he came back to his car a few hours later, he found... | Il s'est mal garé sur le parking, si bien que lorsqu'il est retourné à la voiture quelques heures plus tard, il a trouvé… | Aparcó mal en el parking, así que cuando volvió al coche unas horas después se encontró… | amende: 45%, contravention: 20%, pv: 15%, other(s): 20% | multa: 95%, nota: 5% | amende: 55%, NV: 10%, contravention: 10%, other(s): 25% | billet: 15%, contravention: 15%, amende: 10%, other(s): 60% |
| 50 | For safety reasons, it is mandatory for a motorcycle driver to wear... | Pour des raisons de sécurité, le conducteur de moto doit obligatoirement porter… | Por seguridad, es obligatorio que el conductor de una moto lleve… | casque: 100% | casco: 100% | casque: 100% | casque: 85%, chapeau: 5%, manteau: 5%, permis: 5% |
| 51 | The car tank was almost empty, so he had to stop at... | Le réservoir de la voiture était presque vide, elle a donc du s'arrêter dans… | El tanque del coche estaba casi vacío, así que tuvo que parar en… | station-service: 50%, station-essence: 30%, station: 10%, other(s): 10% | gasolinera: 100% | station-service: 65%, route: 10%, station: 10%, other(s): 15% | station-service: 50%, garage: 15%, station-essence: 15%, other(s): 20% |
| 52 | She already knows how to multiply by 2, 3, and 4, so for tomorrow she has to learn... | Elle sait déjà multiplier par 2, 3 et 4, donc pour demain, elle doit apprendre… | Ya sabe multiplicar por 2, 3 y 4, así que para mañana, ella se tiene que aprender… | cinq: 45%, table: 40%, NV: 10%, multiplication: 5% | tabla: 70%, NV: 30% | cinq: 95%, table: 5% | cinq: 75%, NV: 15%, division: 5%, six: 5% |
| 53 | To avoid forgetting birthdays, she writes them all down in... | Pour n'oublier aucune date d'anniversaire, elle les note toutes sur… | Para no olvidarse de las fechas de cumpleaños, las apunta todas en… | agenda: 35%, calendrier: 30%, téléphone: 15%, other(s): 20% | agenda: 60%, calendario: 35%, móvil: 5% | agenda: 30%, calendrier: 30%, cahier: 10%, other(s): 30% | calendrier: 55%, agenda: 15%, cahier: 5%, other(s): 25% |
| 54 | She had always wanted to be a mother, and now that she's older she wants to have... | Elle a toujours voulu être mère, et maintenant qu'elle est plus âgée, elle veut avoir… | Siempre había querido ser madre, y ahora que es más mayor quiere tener… | enfant: 85%, animal: 5%, bébé: 5%, chien: 5% | hijo: 65%, nieto: 25%, perro: 10% | enfant: 50%, fil: 15%, chien: 10%, other(s): 25% | enfant: 70%, NV: 10%, bébé: 10%, other(s): 10% |
| 55 | He and his friends know how to play instruments, so they decided to start... | Lui et ses amis savent jouer d'un instrument, ils ont donc décidé de créer… | Él y sus amigos saben tocar instrumentos, así que decidieron formar… | groupe: 95%, album: 5% | banda: 60%, grupo: 35%, orquesta: 5% | groupe: 55%, bande: 20%, orchestre: 10%, other(s): 15% | groupe: 60%, bande: 20%, musique: 10%, other(s): 10% |
| 56 | Lucas made a mistake once, but he still deserves... | Lucas s'est trompé une fois mais il mérite encore… | Lucas se equivocó una vez pero todavía se merece… | chance: 85%, NV: 15% | oportunidad: 75%, castigo: 5%, confianza: 5%, other(s): 15% | NV: 40%, chance: 15%, opportunité: 10%, other(s): 35% | chance: 55%, NV: 10%, pardon: 10%, other(s): 25% |
| 57 | She couldn’t stop sneezing, so she thought she must have... | Elle n'arrêtait pas d'éternuer, elle a donc pensé qu'elle avait… | Ella no paraba de estornudar, así que pensó que debía tener… | rhume: 80%, allergie: 10%, NV: 5%, froid: 5% | resfriado: 35%, alergia: 20%, catarro: 20%, other(s): 25% | rhume: 40%, grippe: 15%, malade: 15%, other(s): 30% | rhume: 70%, NV: 5%, allergie: 5%, other(s): 20% |
| 58 | The girl couldn’t sleep because she kept hearing a buzzing around her ear, she thought it must be... | La fille n'arrivait pas à dormir parce qu'elle entendait un bourdonnement près de son oreille, elle a pensé que c'était… | La chica no podía dormir porque no paraba de oír un zumbido alrededor de su oreja, pensó que debía ser… | moustique: 60%, abeille: 15%, insecte: 10%, other(s): 15% | mosquito: 80%, mosca: 10%, abeja: 5%, abejorro: 5% | moustique: 70%, NV: 5%, araignée: 5%, other(s): 20% | moustique: 35%, mouche: 20%, abeille: 15%, other(s): 30% |
| 59 | She finished the exam and gave it to the teacher to grade, now all she can do is wait for... | Il a terminé l'examen et l'a remis au professeur pour qu'il le corrige, il ne lui reste plus qu'à attendre… | Acabó el examen y se lo dio al profesor para que lo corrigiese, ahora solo le queda esperar… | résultat: 60%, note: 25%, correction: 15% | nota: 70%, calificación: 10%, resultado: 10%, other(s): 10% | note: 40%, résultat: 25%, professeur: 10%, other(s): 25% | résultat: 85%, note: 10%, NV: 5% |
| 60 | The building has many floors so, in addition to stairs, it also has... | Le bâtiment comporte beaucoup d'étages donc, en plus des escaliers, il y a aussi… | El edificio tiene muchas plantas así que, además de escaleras, tiene también… | ascenseur: 100% | ascensor: 100% | ascenseur: 95%, garde-corps: 5% | ascenseur: 100% |
| 61 | Rubén’s/Ethan’s office was so big that it even had, for his meetings, several chairs and... | Le bureau de Ethan était si grand qu'il y avait même, pour ses réunions, plusieurs chaises et… | La oficina de Rubén era tan grande que incluso tenía, para sus reuniones, varias sillas y… | table: 60%, bureau: 10%, fauteuil: 10%, other(s): 20% | mesa: 100% | table: 75%, canapé: 10%, écran: 5%, other(s): 10% | table: 75%, bureau: 15%, invité: 5%, tableau: 5% |
| 62 | The place the girl was looking for was one block away, to get there, she just had to cross... | L'endroit que la fille cherchait se trouvait à un pâté de maison, pour y arriver elle n'avait qu'à traverser… | El sitio que buscaba la chica estaba a una manzana, para llegar solo tenía que cruzar… | rue: 80%, route: 10%, pont: 5%, quartier: 5% | calle: 85%, avenida: 5%, carretera: 5%, esquina: 5% | rue: 70%, chemin: 5%, fleve: 5%, other(s): 20% | rue: 65%, route: 15%, jardin: 10%, other(s): 10% |
| 63 | Ángel’s house had been robbed twice, so to increase security he installed... | La maison de Bastien a été cambriolée déjà deux fois, donc pour renforcer la sécurité, il a installé… | Habían robado dos veces en la casa de Ángel, así que para reforzar la seguridad instaló… | alarme: 50%, caméra: 40%, système: 10% | alarma: 55%, camara: 40%, sistema: 5% | alarme: 50%, caméra: 45%, clôture: 5% | caméra: 45%, alarme: 40%, cctv: 5%, other(s): 10% |
| 64 | She wants to stay after the concert to ask the singer to sign... | Elle veut rester après le concert pour demander au chanteur de lui signer… | Ella se quiere quedar después del concierto para pedirle al cantante que le firme… | autographe: 85%, album: 5%, sein: 5%, t-shirt: 5% | autógrafo: 55%, disco: 25%, camiseta: 10%, other(s): 10% | autographe: 50%, album: 10%, cd: 10%, other(s): 30% | autographe: 55%, papier: 10%, photo: 10%, other(s): 25% |
| 65 | He loves football, so for his birthday his girlfriend gave him tickets for... | Il adore le football, et pour son anniversaire, son amie lui a offert des billets pour… | Le encanta el fútbol, así que en su cumpleaños su novia le regaló entradas para… | match: 85%, coupe: 10%, finale: 5% | partido: 90%, final: 5%, real madrid: 5% | match: 85%, espagne: 5%, finale: 5%, partie: 5% | match: 65%, jeu: 15%, équipe: 10%, other(s): 10% |
| 66 | She needs to take a lot of vitamin C, so every morning she makes herself... | Elle a besoin de beaucoup de vitamine C, alors chaque matin elle se prépare… | Necesita tomar mucha vitamina C, así que cada mañana ella se prepara… | jus: 90%, déjeuner: 5%, vitamine: 5% | zumo: 90%, batido: 10% | jus: 90%, NV: 5%, verre: 5% | jus: 60%, orange: 15%, bol: 5%, other(s): 20% |
| 67 | The widow goes to the cemetery every few months and places flowers on... | Régulièrement, la veuve se rend au cimetière et dépose des fleurs sur… | La viuda cada pocos meses va al cementerio y coloca flores en… | tombe: 50%, mari: 40%, défunt: 10% | tumba: 80%, lápida: 15%, nicho: 5% | tombe: 60%, mari: 10%, tombeau: 10%, other(s): 20% | tombe: 45%, mari: 30%, cercueil: 5%, other(s): 20% |
| 68 | Martín/Martin started practicing fencing just to learn how to use... | Martin a commencé l'escrime pour apprendre à utiliser… | Martín empezó a practicar esgrima solo para aprender a utilizar… | épée: 55%, sabre: 20%, fleuret: 10%, other(s): 15% | espada: 60%, florete: 35%, estoque: 5% | épée: 65%, NV: 10%, crayon: 5%, other(s): 20% | épée: 70%, sabre: 20%, NV: 5%, fleuret: 5% |
| 69 | When visiting her grandmother, Little Red Riding Hood was surprised by... | En allant visiter sa grand-mère, le petit Chaperon Rouge s'est fait surprendre par… | Al visitar a su abuela, Caperucita Roja fue sorprendida por… | loup: 100% | lobo: 100% | loup: 90%, ami: 5%, tantes: 5% | loup: 55%, NV: 5%, âge: 5%, other(s): 35% |
| 70 | The air conditioning was broken and it was so hot that Isabel had to open... | La climatisation était en panne et il faisait tellement chaud que Isabelle a du ouvrir… | El aire acondicionado estaba roto y hacía tanto calor que Isabel tuvo que abrir… | fenêtre: 95%, corsage: 5% | ventana: 95%, frigorífico: 5% | fenêtre: 100% | fenêtre: 100% |
| 71 | To dig the hole in the garden, he needs... | Pour creuser le trou dans le jardin, il lui faut… | Para cavar el hoyo en el jardín, necesita… | pelle: 95%, outil: 5% | pala: 95%, NV: 5% | pelle: 80%, arbre: 5%, chemin: 5%, other(s): 10% | pelle: 85%, bêche: 5%, creuser: 5%, jardinage: 5% |
| 72 | The pop band has already written many songs, they could record... | Le groupe de musique pop a déjà écrit de nombreuses chansons, il pourrait enregistrer… | El grupo de música pop ha escrito ya muchas canciones, podrían grabar… | album: 85%, disque: 10%, NV: 5% | disco: 80%, álbum: 15%, videoclip: 5% | disque: 35%, album: 25%, NV: 5%, other(s): 35% | album: 45%, NV: 35%, discographie: 5%, other(s): 15% |
| 73 | María/Manon didn’t want to go to Juan’s party, so to avoid it she made up... | Manon ne voulait pas aller à la fête de Jean, pour éviter d'y aller, elle s'est inventé… | María no quería ir a la fiesta de Juan, así que para evitarlo se inventó… | excuse: 70%, maladie: 10%, histoire: 5%, other(s): 15% | excusa: 90%, NV: 5%, indisponibilidad: 5% | excuse: 80%, maladie: 10%, NV: 5%, histoire: 5% | excuse: 60%, maladie: 20%, histoire: 5%, other(s): 15% |
| 74 | Every morning, the farmer collects the fresh eggs from... | Chaque matin, la fermière ramasse les œufs frais dans… | Cada mañana, la granjera coge los huevos frescos en… | poulailler: 95%, panier: 5% | gallinero: 40%, corral: 20%, granja: 15%, other(s): 25% | poule: 20%, ferme: 15%, panier: 15%, other(s): 50% | poulailler: 40%, poule: 20%, poulet: 15%, other(s): 25% |
| 75 | A unicycle is like a bicycle, but it has only... | Un monocycle ressemble à une bicyclette, mais il a seulement… | Un monociclo es como una bicicleta, pero tiene solo… | roue: 100% | rueda: 100% | roue: 90%, cyclé: 5%, moteur: 5% | roue: 85%, pneu: 10%, moteur: 5% |
| 76 | When she’s home alone, she likes to ask who it is before opening... | Quand elle est seule à la maison, elle préfère demander qui c'est avant d'ouvrir… | Cuando está sola en casa, le gusta preguntar quién es antes de abrir… | porte: 100% | puerta: 95%, seguridad: 5% | porte: 100% | porte: 85%, NV: 10%, personne: 5% |
| 77 | The place where Jaime lives is very small, but his daughter will have more space to play in the garden when they buy... | Le logement où Paul vit est très petit, mais sa fille aura plus d'espace pour jouer dans le jardin quand ils achèteront… | La vivienda donde vive Jaime es muy pequeña, pero su hija tendrá más espacio para jugar en el jardín cuando compren… | maison: 90%, piscine: 5%, villa: 5% | casa: 65%, chalet: 10%, adosado: 5%, other(s): 20% | maison: 85%, terrain: 10%, chien: 5% | maison: 85%, pelouse: 5%, ranch: 5%, table: 5% |
| 78 | She wanted to find a better job, so she decided to leave the countryside and live in... | Elle voulait chercher un meilleur emploi et a donc décidé de quitter la campagne pour aller s'installer dans… | Quería buscar un trabajo mejor, así que ella decidió dejar el campo para vivir en… | ville: 70%, appartement: 5%, capitale: 5%, other(s): 20% | ciudad: 90%, capital: 5%, madrid: 5% | ville: 90%, centre: 5%, cité: 5% | ville: 100% |
| 79 | This woman has her hair covered, wears a long black robe, and a big cross on her neck, she must be... | Cette femme a les cheveux couverts, une longue robe noire et une grande croix autour du coup, ce doit être… | Esta mujer tiene el cabello tapado, una túnica larga negra y una cruz grande en el cuello, debe de ser… | nonne: 55%, sœur: 20%, religieuse: 10%, other(s): 15% | monja: 95%, NV: 5% | religieuse: 35%, nonne: 30%, NV: 20%, other(s): 15% | nonne: 30%, religieuse: 30%, sœur: 15%, other(s): 25% |
| 80 | When they stay at the hotel, they always swim in... | Lorsqu'ils séjournent à l'hôtel, ils vont toujours nager dans… | Cuando se quedan en el hotel, siempre nadan en… | piscine: 90%, mer: 10% | piscina: 100% | piscine: 95%, spa: 5% | piscine: 100% |
| 81 | Inés/Inès drew four equal lines to create... | Inès a dessiné quatre lignes égales pour créer… | Inés dibujó cuatro líneas iguales para crear… | carré: 70%, barrière: 5%, colonne: 5%, other(s): 20% | cuadrado: 85%, carretera: 5%, casa: 5%, dibujo: 5% | carré: 70%, dessin: 10%, cadre: 5%, other(s): 15% | carré: 90%, rectangle: 5%, table: 5% |
| 82 | At the end of the présentation, the student raised her hand because she had... | A la fin de la présentation, l'étudiante a levé la main car elle avait… | Al final de la presentación, la alumna levantó la mano porque tenía… | question: 95%, crampe: 5% | duda: 60%, pregunta: 40% | question: 65%, doute: 15%, NV: 5%, other(s): 15% | question: 95%, ami: 5% |
| 83 | Children are always taught to look both ways before crossing... | On apprend toujours aux enfants à regarder des deux côtés avant de traverser… | Siempre se enseña a los niños a mirar a los dos lados antes de cruzar… | route: 60%, rue: 30%, passage: 10% | calle: 45%, carretera: 35%, paso: 10%, other(s): 10% | rue: 70%, route: 25%, passage: 5% | route: 50%, rue: 45%, escalier: 5% |
| 84 | Since she wanted to write her secrets, she went to the bookstore to buy... | Comme elle voulait écrire ses secrets, elle est allée à la papeterie acheter… | Como quería escribir sus secretos, fue a la librería a comprarse… | journal: 40%, carnet: 35%, cahier: 10%, other(s): 15% | diario: 80%, cuaderno: 20% | journal: 75%, cahier: 20%, NV: 5% | journal: 75%, stylo: 15%, carnet: 5%, papier: 5% |
| 85 | Tulips of all colors have grown in... | Des tulipes de toutes les couleurs ont poussé dans… | Han crecido tulipanes de todos los colores en… | jardin: 80%, champ: 15%, parterre: 5% | jardín: 70%, campo: 20%, holanda: 5%, patio: 5% | jardin: 50%, chambre: 5%, champ: 5%, other(s): 40% | jardin: 70%, champ: 15%, herbe: 5%, other(s): 10% |
| 86 | She was very thirsty so when she got to the bar she ordered... | Elle avait très soif, donc quand elle est rentrée dans le bar, elle a demandé… | Ella tenía mucha sed así que cuando llegó al bar pidió… | verre: 70%, eau: 15%, NV: 5%, other(s): 10% | vaso: 40%, agua: 35%, cerveza: 15%, refresco: 10% | eau: 40%, verre: 25%, bière: 15%, other(s): 20% | eau: 30%, verre: 30%, boisson: 25%, other(s): 15% |
| 87 | The teacher asked all her students to take out a sheet of paper and... | La professeure a demandé à tous ses élèves de sortir une feuille et… | La profesora pidió a todos sus alumnos sacar un papel y… | stylo: 60%, NV: 15%, crayon: 15%, other(s): 10% | bolígrafo: 55%, lápiz: 25%, NV: 10%, other(s): 10% | stylo: 30%, NV: 25%, crayon: 25%, other(s): 20% | NV: 40%, stylo: 40%, crayon: 5%, other(s): 15% |
| 88 | Since it was Valentine’s Day, Sergio/Tom bought a heart-shaped mold to make... | Comme c'était la Saint-Valentin, Tom a acheté un moule en forme de cœur pour faire… | Como era San Valentín, Sergio compró un molde con forma de corazón para hacer… | gâteau: 65%, plaisir: 15%, brownie: 5%, other(s): 15% | tarta: 50%, pastel: 30%, bizcocho: 15%, bombón: 5% | gâteau: 35%, cadeau: 25%, tarte: 20%, other(s): 20% | gâteau: 55%, cadeau: 20%, NV: 5%, other(s): 20% |
| 89 | The woman started to smell something burning because she had forgotten the chicken in... | La femme a commencé à sentir le brûlé parce qu'elle avait oublié le poulet dans… | La mujer empezó a oler a quemado porque se había olvidado el pollo en… | four: 95%, poêle: 5% | horno: 80%, fuego: 10%, fogón: 5%, sartén: 5% | four: 75%, feu: 15%, casserole: 5%, frigo: 5% | four: 100% |
| 90 | On Halloween, kids carve eyes and a mouth in... | Pour Halloween, les enfants sculptent des yeux et une bouche dans… | En Halloween los niños tallan unos ojos y una boca en… | citrouille: 100% | calabaza: 95%, papel: 5% | citrouille: 75%, courge: 15%, NV: 5%, porte: 5% | citrouille: 80%, NV: 15%, masque: 5% |
| 91 | The cake didn’t rise in the oven because they had forgotten to add... | Le gâteau n'avait pas gonflé dans le four parce qu'ils avaient oublié d'ajouter… | El bizcocho no había crecido en el horno porque se les había olvidado añadir… | levure: 85%, farine: 10%, beurre: 5% | levadura: 90%, harina: 10% | levure: 80%, farine: 5%, ingrédient: 5%, other(s): 10% | levure: 75%, NV: 5%, bicarbonate: 5%, other(s): 15% |
| 92 | When baking chocolate cookies, Carla always follows... | Lorsqu'elle cuisine des biscuits au chocolat, Carla suit toujours… | Al cocinar galletas de chocolate, Carla siempre sigue… | recette: 95%, étape: 5% | receta: 95%, indicación: 5% | recette: 70%, blog: 5%, chocolat: 5%, other(s): 20% | recette: 80%, NV: 5%, chef: 5%, other(s): 10% |
| 93 | She cleaned up the milk she had spilled on the counter with... | Elle a essuyé le lait qu'elle avait renversé sur le comptoir avec… | Limpió la leche que había derramado en la encimera con… | éponge: 30%, chiffon: 25%, torchon: 20%, other(s): 25% | trapo: 35%, bayeta: 30%, paño: 30%, servilleta: 5% | chiffon: 25%, serviette: 15%, tissu: 15%, other(s): 45% | serviette: 35%, tissu: 30%, chiffon: 10%, other(s): 25% |
| 94 | When she looked in the mirror, she realized her white pants had... | En se regardant dans le miroir, elle s'est rendue compte que son pantalon blanc avait… | Al mirarse en el espejo ella se dio cuenta de que sus pantalones blancos tenían… | tâche: 100% | mancha: 100% | tâche: 70%, NV: 20%, chemise: 5%, réduit: 5% | tâche: 80%, NV: 10%, chemise: 5%, marque: 5% |
| 95 | It was so cold that before leaving the house she put on gloves, a hat and... | Il faisait si froid qu'avant de sortir de la maison, elle a mis des gants, un bonnet et… | Hacía tanto frío que antes de salir de casa ella se puso los guantes, el gorro y… | écharpe: 90%, doudoune: 5%, manteau: 5% | bufanda: 85%, abrigo: 10%, chaquetón: 5% | écharpe: 50%, manteau: 30%, foulard: 15%, plateau: 5% | écharpe: 45%, manteau: 35%, chapeau: 10%, other(s): 10% |
| 96 | The evil witch made Snow White bite... | La méchante sorcière a fait que Blanche-Neige a croqué… | La malvada bruja hizo que Blancanieves mordiese… | pomme: 100% | manzana: 100% | pomme: 85%, miroir: 5%, mort: 5%, vie: 5% | pomme: 90%, chat: 5%, chocolat: 5% |
| 97 | In some countries, they screw furniture to the wall in case there’s... | Dans certains pays, on visse les meubles au mur au cas où se produise… | En algunos países, atornillan los muebles a la pared por si ocurre… | tremblement: 75%, séisme: 15%, inondation: 5%, secousse: 5% | terremoto: 90%, accidente: 5%, volcado: 5% | séisme: 35%, tremblement: 25%, NV: 15%, other(s): 25% | tremblement: 60%, accident: 10%, séisme: 10%, other(s): 20% |
| 98 | The winners of the football championship received... | Les vainqueurs du championnat de football ont reçu… | Los ganadores del campeonato de fútbol recibieron… | coupe: 45%, trophée: 35%, médaille: 15%, poids: 5% | trofeo: 65%, medalla: 25%, copa: 5%, premio: 5% | trophée: 55%, prix: 15%, coupe: 10%, other(s): 20% | trophée: 55%, coupe: 25%, médaille: 15%, prix: 5% |
| 99 | On top of the wedding cake, there was... | Tout en haut du gâteau de mariage, il y avait… | Arriba de la tarta de la boda, había… | figure: 40%, cerise: 10%, couple: 10%, other(s): 40% | figura: 70%, muñeco: 15%, pareja: 10%, novio: 5% | figure: 60%, ornement: 10%, poupée: 10%, other(s): 20% | figure: 30%, couple: 15%, marié: 15%, other(s): 40% |
| 100 | The girl was eagerly waiting for the clown to finish inflating... | La petite fille attendait impatiement que le clown finisse de gonfler… | La niña esperaba ansiosamente a que el payaso terminase de inflar… | ballon: 95%, structure: 5% | globo: 100% | ballon: 95%, globe: 5% | ballon: 95%, NV: 5% |
| 101 | For her shirt to close up to the neck, she has to sew on... | Pour que sa chemise ferme bien jusqu'au cou, il doit lui coudre… | Para que su camisa cierre hasta el cuello, le tiene que coser… | bouton: 85%, col: 5%, fermeture: 5%, tissu: 5% | botón: 90%, cremallera: 5%, solapa: 5% | bouton: 70%, NV: 10%, fermeture: 5%, other(s): 15% | bouton: 55%, NV: 30%, ensemble: 5%, other(s): 10% |
| 102 | To get the lasagna nicely browned and crispy, at the end you have to add... | Pour que les lasagnes sortent bien dorées et grillées du four, il ne faut pas oublier à la fin de mettre… | Para que la lasaña salga bien dorada y tostada del horno, al final hay que poner… | fromage: 55%, grill: 25%, huîle: 10%, other(s): 10% | queso: 50%, grill: 30%, aceite: 5%, other(s): 15% | fromage: 50%, grill: 25%, œuf: 15%, other(s): 10% | fromage: 70%, grill: 10%, beurre: 5%, other(s): 15% |
| 103 | To cut the skin at the beginning of the operation, the surgeon asked the nurse to pass him... | Pour couper la peau au début de l'opération, le chirurgien a demandé à l'infirmière qu'elle lui passe… | Para cortar la piel al principio de la operación, el cirujano le pidió a la enfermera que le pasara… | scalpel: 80%, bistouri: 10%, bethadine: 5%, ciseaux: 5% | bisturí: 85%, betadine: 5%, desinfectante: 5%, tijeras: 5% | scalpel: 55%, bistouri: 15%, ciseaux: 10%, other(s): 20% | scalpel: 70%, couteau: 10%, anesthésie: 5%, other(s): 15% |
| 104 | After bathing, she always dries off with... | Après le bain, elle se sèche toujours avec… | Después de bañarse, ella siempre se seca con… | serviette: 85%, langue: 5%, peignoir: 5%, sèche-cheveux: 5% | toalla: 85%, albornoz: 10%, secador: 5% | serviette: 90%, NV: 5%, peignoir: 5% | serviette: 100% |
| 105 | The pine is a tree and the rose is... | Le pin est un arbre et la rose est… | El pino es un árbol y la rosa es… | fleur: 100% | flor: 100% | fleur: 90%, plante: 10% | fleur: 100% |
| 106 | After withdrawing money at the bank, the woman opened her purse to put it in... | Après avoir retiré de l'argent à la banque, la femme a ouvert son sac pour le mettre dans… | Después de sacar dinero en el banco, la mujer abrió el bolso para guardarlo en… | portefeuille: 70%, porte-monnaie: 10%, compartiment: 5%, other(s): 15% | cartera: 45%, monedero: 45%, bolsillo: 5%, bolso: 5% | portefeuille: 65%, sac: 10%, argent: 5%, other(s): 20% | portefeuille: 40%, sac: 30%, porte-monnaie: 20%, other(s): 10% |
| 107 | To air out the room while making the bed, she opens... | Pour aérer la pièce pendant qu'elle fait son lit, elle ouvre… | Para ventilar la habitación mientras hace la cama, ella abre… | fenêtre: 100% | ventana: 95%, puerta: 5% | fenêtre: 100% | fenêtre: 95%, porte: 5% |
| 108 | She was very thirsty, but luckily she came across... in the middle of the street | Il avait très soif, mais par chance, au milieu de la rue il est tombé sur… | Tenía mucha sed, pero por suerte se topó en mitad de la calle con… | fontaine: 60%, bouteille: 10%, distributeur: 10%, other(s): 20% | fuente: 85%, amigo: 5%, máquina: 5%, tienda: 5% | fontaine: 35%, NV: 10%, eau: 10%, other(s): 45% | fontaine: 70%, café: 10%, NV: 5%, other(s): 15% |
| 109 | He knew she was married because she wore a ring on... | Il était facile de savoir qu'elle était mariée puisqu'elle portait un anneau sur… | Él sabía que estaba casada porque llevaba un anillo en … | annulaire: 70%, doigt: 25%, index: 5% | dedo: 90%, mano: 10% | doigt: 75%, annulaire: 10%, main: 10%, bout: 5% | doigt: 65%, main: 20%, annulaire: 10%, mariage: 5% |
| 110 | The only way to get to the other side of the river is to cross... | Le seul moyen d'aller de l'autre côté de la rivière est de traverser… | La única manera de pasar al otro lado del río es cruzar… | pont: 85%, gué: 5%, nage: 5%, rivière: 5% | puente: 70%, NV: 20%, barca: 5%, roca: 5% | pont: 65%, bateau: 15%, rivière: 10%, other(s): 10% | pont: 60%, bateau: 20%, NV: 5%, other(s): 15% |
| 111 | To make a purchase on this website, she needs to pay with... | Pour faire des achats sur ce site web, elle doit payer avec… | Para hacer una compras en esta página web, ella necesita pagar con… | carte: 100% | tarjeta: 100% | carte: 75%, NV: 5%, argent: 5%, other(s): 15% | carte: 95%, livre: 5% |
| 112 | When you've been in a relationship for many years, it's very hard to break... | Quand on est avec son partenaire depuis de nombreuses années, il est très difficile d'interrompre… | Cuando uno lleva muchos años con pareja, es muy difícil cortar… | relation: 40%, conversation: 10%, habitude: 10%, other(s): 40% | relación: 75%, NV: 15%, contacto: 5%, rutina: 5% | relation: 50%, NV: 10%, amour: 5%, other(s): 35% | routine: 25%, relation: 20%, NV: 15%, other(s): 40% |
| 113 | She’s lactose intolerant, that’s why milk, yogurt and... make her feel sick | Elle est allergique au lactose, c'est pourquoi elle ne peut pas consommer le lait, le yaourt et… | Es alérgica a la lactosa, por eso le sientan mal la leche, el yogurt y… | fromage: 80%, crème: 10%, gâteau: 5%, produit: 5% | queso: 100% | fromage: 95%, produit: 5% | fromage: 85%, crème: 5%, glace: 5%, produit: 5% |
| 114 | Her parents have a big house and can park both cars in... | Ses parents ont une grande maison et peuvent garer les deux voitures dans… | Sus padres tienen una casa grande y pueden aparcar los dos coches en… | garage: 90%, allée: 5%, jardin: 5% | garaje: 95%, interior: 5% | garage: 100% | garage: 80%, allée: 15%, ligne: 5% |
| 115 | She loves music, that’s why her friend offered her a ticket to go to... | Elle adore la musique, pour ça son amie lui a offert un billet pour aller à… | Le encanta la música, por eso su amiga le ofreció una entrada para ir a… | concert: 80%, festival: 10%, ópera: 5%, vienne: 5% | concierto: 95%, ópera: 5% | concert: 65%, ópera: 30%, salzburg: 5% | concert: 95%, ópera: 5% |
| 116 | Paula had a new phone, and to try it out she opened WhatsApp to send... | Lisa avait un nouveau téléphone portable, et pour le tester, elle a ouvert whatsapp et envoyé… | Paula tenía un móvil nuevo, y para probarlo abrió whatsapp para enviar… | message: 100% | mensaje: 100% | message: 95%, photo: 5% | message: 80%, texte: 15%, photo: 5% |
| 117 | She doesn't know how to cook, so to celebrate their anniversary she invited her wife to dinner at... | Il ne sait pas cuisiner, alors pour fêter leur anniversaire, il a invité sa femme à dîner dans… | No sabe cocinar, entonces para celebrar su aniversario invitó a su esposa a cenar en… | restaurant: 100% | restaurante: 100% | restaurant: 95%, bistro: 5% | restaurant: 85%, NV: 5%, café: 5%, chef: 5% |
| 118 | Ramón/Gérard had filled the jacuzzi to the top to take... | Gérard avait rempli le jacuzzi à ras bord pour prendre… | Ramón había llenado hasta arriba el jacuzzi para darse… | bain: 90%, pied: 5%, température: 5% | baño: 100% | bain: 80%, NV: 10%, chauffe: 5%, soleil: 5% | bain: 90%, douche: 5%, trempage: 5% |
| 119 | She used to go to church every Sunday, but since her son died she stopped going because she has lost... | Elle allait à l'église tous les dimanches, mais depuis la mort de son fils, elle a cessé d'y aller parce qu'elle a perdu… | Iba a la iglesia cada domingo, pero desde que murió su hijo ha dejado de ir porque ha perdido… | foi: 95%, envie: 5% | fe: 100% | foi: 85%, NV: 5%, désir: 5%, motivation: 5% | foi: 75%, enfant: 5%, envie: 5%, other(s): 15% |
| 120 | The food was ready so the mother told her son to set... | Le repas était prêt, donc la mère a ordonné à son fils de mettre… | La comida estaba preparada así que la madre ordenó al hijo que pusiera… | table: 85%, couvert: 15% | mesa: 80%, plato: 10%, cubierto: 5%, microondas: 5% | table: 95%, couvert: 5% | table: 70%, plat: 10%, tableau: 10%, other(s): 10% |
| 121 | Pinocchio is a puppet and Barbie is... | Pinocchio est une marionnette et Barbie est… | Pinocho es una marioneta y Barbie es… | poupée: 100% | muñeca: 100% | poupée: 100% | poupée: 100% |
| 122 | Her grandmother loves to pet calm and affectionate animals, so they gave her... | Sa grand-mère adore caresser les animaux tranquilles et câlins, alors ils lui ont offert… | A su abuela le encanta acariciar a los animales tranquilos y cariñosos, así que le han regalado… | chat: 60%, chien: 30%, chiot: 5%, lapin: 5% | gato: 65%, perro: 30%, cachorro: 5% | chat: 50%, chien: 25%, NV: 5%, other(s): 20% | chat: 60%, lapin: 20%, chien: 15%, pièce: 5% |
| 123 | To go from New York airport to London, she chose... | Pour se rendre de l'aéroport de New-York à Londres, elle a choisi… | Para ir desde el aeropuerto de Nueva York hasta Londres, ella ha elegido… | taxi: 25%, vol: 25%, avion: 15%, other(s): 35% | avión: 40%, vuelo: 20%, british airways: 10%, other(s): 30% | avion: 40%, taxi: 20%, vol: 20%, other(s): 20% | avion: 40%, vol: 25%, taxi: 20%, other(s): 15% |
| 124 | All of Laura’s plants need water daily, except the succulent and... | Toutes les plantes de Laura doivent être arrosées tous les jours, à part la plante grasse et… | Todas las plantas de Laura necesitan agua diariamente, menos la suculenta y… | cactus: 65%, plante: 15%, ciboulette: 5%, other(s): 15% | cactus: 85%, planta: 10%, preservada: 5% | cactus: 55%, NV: 10%, couper: 5%, other(s): 30% | cactus: 55%, NV: 15%, fleurie: 5%, other(s): 25% |
| 125 | One of the most important human organs to live is... | Un des organes humains les plus importants pour vivre est… | Uno de los órganos humanos más importante para vivir es… | coeur: 90%, cerveau: 5%, foie: 5% | corazón: 90%, cerebro: 5%, páncreas: 5% | coeur: 75%, cerveau: 15%, peau: 5%, poumon: 5% | coeur: 100% |
| 126 | She couldn’t get into the office because she had forgotten... | Il ne pouvait pas entrer dans son bureau parce qu'il avait oublié… | No podía entrar en el despacho porque había olvidado… | clé: 95%, badge: 5% | llave: 95%, tarjeta: 5% | clé: 90%, NV: 5%, carte: 5% | clé: 90%, carte: 10% |
| 127 | Children build sandcastles in... | Les enfants font des châteaux de sable sur… | Los niños hacen castillos de arena en… | plage: 95%, sable: 5% | playa: 100% | plage: 85%, sable: 10%, rivière: 5% | plage: 100% |
| 128 | To look presentable, he shaves very regularly... | Pour être présentable, il se rase très régulièrement… | Para estar presentable, se afeita con mucha regularidad… | barbe: 75%, visage: 10%, blanc: 5%, other(s): 10% | barba: 75%, cara: 10%, casa: 5%, other(s): 10% | barbe: 90%, cheveu: 5%, visage: 5% | barbe: 50%, visage: 20%, NV: 5%, other(s): 25% |
| 129 | After stepping on a venomous spider, Julio/Adrien had a rash on... | Après avoir marché sur une araignée venimeuse, Adrien a eu une irritation sur… | Después de haber pisado una araña venenosa, Julio tuvo una irritación en… | pied: 60%, plante: 20%, peau: 15%, jambe: 5% | pie: 75%, planta: 20%, pierna: 5% | pied: 50%, peau: 40%, jambe: 5%, peu: 5% | pied: 50%, jambe: 15%, peau: 15%, other(s): 20% |
| 130 | To cut meat you need... | Pour couper la viande, il faut… | Para cortar la carne se necesita… | couteau: 95%, hachoir: 5% | cuchillo: 100% | couteau: 100% | couteau: 90%, attendrir: 5%, four: 5% |
| 131 | When they won the final, the team captain lifted... | Lorsqu'ils ont gagné la finale, le capitaine de l'équipe a levé… | Cuando ganaron la final, el capitán del equipo levantó… | coupe: 60%, poing: 15%, NV: 5%, other(s): 20% | copa: 65%, trofeo: 30%, sonrisa: 5% | coupe: 50%, trophée: 30%, bras: 10%, other(s): 10% | trophée: 75%, main: 10%, bras: 5%, other(s): 10% |
| 132 | The king was proud to wear … on his head | Le roi était fier de porter sur la tête… | El rey estaba orgulloso de llevar en la cabeza… | couronne: 100% | corona: 100% | couronne: 100% | couronne: 95%, NV: 5% |
| 133 | The clothes were dirty, so he put them in... | Les vêtements étaient sales, il les a donc mis dans… | La ropa estaba sucia, así que él la puso en… | machine-a-laver: 40%, machine: 15%, corbeille: 10%, other(s): 35% | lavadora: 85%, cesta: 15% | machine-a-laver: 70%, lave-linge: 15%, NV: 5%, other(s): 10% | machine-a-laver: 80%, basket: 5%, lave-vêtements: 5%, other(s): 10% |
| 134 | When he saw his father, he turned his cheek because he expected to receive... | Quand il a vu son père, il a tendu la joue parce qu'il s'attendait à recevoir… | Al ver a su padre, puso la mejilla porque esperaba recibir… | bise: 35%, gifle: 25%, baffe: 10%, other(s): 30% | beso: 80%, bofetada: 10%, guantazo: 5%, tortazo: 5% | bisou: 50%, baiser: 10%, cadeau: 10%, other(s): 30% | baiser: 30%, bisou: 30%, gifle: 10%, other(s): 30% |
| 135 | Her grandmother liked to stay informed so every day at news time she turned on... | Sa grand-mère aimait être informée, c'est pourquoi chaque jour, à l'heure du journal, elle allumait… | A su abuela le gustaba estar informada así que cada día, a la hora del informativo, encendía… | télévision: 85%, journal: 5%, poste: 5%, radio: 5% | televisión: 80%, televisor: 15%, noticia: 5% | télévision: 75%, radio: 20%, feu: 5% | télévision: 65%, lumière: 15%, radio: 10%, other(s): 10% |
| 136 | Miguel/Louis loves science fiction, whenever he sees something flying in the sky he thinks it’s... | Louis adore la science fiction, chaque fois qu'il voit un objet volant dans le ciel il pense que c'est… | A Miguel le encanta la ciencia ficción, siempre que ve un objeto en el cielo volando piensa que es… | ovni: 75%, vaisseau: 15%, soucoupe: 10% | ovni: 75%, nave: 20%, plato: 5% | ovni: 75%, alien: 10%, aventure: 5%, other(s): 10% | ovni: 35%, vaisseau: 25%, NV: 15%, other(s): 25% |
| 137 | At the Louvre Museum, the Venus de Milo is... | Au musée du Louvre, la Vénus de Milo est… | En el museo Louvre, la Venus de Milo es… | exposé: 40%, statue: 25%, NV: 15%, other(s): 20% | estatua: 30%, NV: 25%, obra: 15%, other(s): 30% | NV: 20%, sculpture: 20%, cadre: 10%, other(s): 50% | NV: 25%, exposé: 25%, statue: 15%, other(s): 35% |
| 138 | Everything went dark because the ... had gone out | Tout est devenu sombre parce qu'on avait éteint… | Todo quedó a oscuras porque se había apagado… | lumière: 85%, cigarette: 5%, eclairage: 5%, lampe: 5% | luz: 90%, vela: 10% | lumière: 75%, lampe: 15%, leux: 5%, lume: 5% | lumière: 80%, NV: 5%, animal: 5%, other(s): 10% |
| 139 | Several bank managers called him after he won a lot of money in... | Les directeurs de plusieurs banques l'ont appelé après qu'il a gagné beaucoup d'argent à… | Le llamaron los directores de varios bancos después de que le tocase mucho dinero en… | loterie: 65%, jeu: 10%, NV: 5%, other(s): 20% | lotería: 100% | loterie: 55%, lotto: 15%, NV: 10%, other(s): 20% | loterie: 50%, argent: 5%, barbecue: 5%, other(s): 40% |
| 140 | In general, tea is drunk in... | En général, le thé se boit dans… | En general, el té se bebe en… | tasse: 90%, eau: 5%, mug: 5% | taza: 85%, caliente: 10%, invierno: 5% | tasse: 90%, NV: 5%, verre: 5% | tasse: 90%, matinée: 5%, verre: 5% |
| 141 | Near the hospital, she heard a siren approaching, so she pulled over because ... was probably arriving | Près de l'hôpital, il a entendu une sirène qui s'approchait, il s'est donc mis sur le côté car arrivait certainement… | Cerca del hospital, escuchó una sirena acercándose, así que apartó el coche porque seguramente llegaba… | ambulance: 85%, pompier: 10%, police: 5% | ambulancia: 85%, NV: 15% | ambulance: 70%, NV: 25%, voiture: 5% | ambulance: 65%, NV: 35% |
| 142 | Last winter, the mountain roads were closed because it was impossible to drive on... | L'hiver passé, les routes de montagne ont été fermées parce que la circulation était rendue impossible par… | El invierno pasado, las carreteras de montaña se cerraron ya que era imposible circular por… | neige: 65%, NV: 15%, abondance: 5%, other(s): 15% | nieve: 50%, NV: 30%, hielo: 10%, other(s): 10% | neige: 90%, NV: 5%, endroit: 5% | neige: 65%, glace: 10%, bloquage: 5%, other(s): 20% |
| 143 | For more than 10 years, every winter there is a campaign to vaccinate elderly people against… | Depuis 10 ans, chaque hiver a lieu une campagne de vaccination des personnes âgées contre… | Desde más de 10 años, cada invierno se hace una campaña para vacunar a la gente mayor contra… | grippe: 95%, virus: 5% | gripe: 100% | grippe: 80%, covid: 10%, influenza: 5%, rhume: 5% | grippe: 75%, covid: 10%, influenza: 5%, other(s): 10% |
| 144 | She was tired of cooking standing up, so she sat down on… | Elle était fatiguée de cuisiner debout, donc elle s'est assise sur… | Estaba cansada de cocinar de pie, por eso se sentó en… | chaise: 70%, tabouret: 25%, table: 5% | silla: 55%, taburete: 45% | chaise: 80%, canapé: 10%, restaurant: 5%, tabouret: 5% | chaise: 70%, tabouret: 15%, boudoir: 5%, other(s): 10% |
| 145 | When he was just a child, his grandfather planted … in the forest | Lorsqu'il n'était qu'un enfant, son grand-père a planté dans la forêt… | Cuando era sólo un niño su abuelo plantó en el bosque… | arbre: 60%, sapin: 10%, NV: 5%, other(s): 25% | árbol: 65%, roble: 15%, pino: 10%, other(s): 10% | arbre: 85%, pin: 10%, plante: 5% | arbre: 100% |
| 146 | The base of most Asian dishes is… | La base de la plupart des plats asiatiques est… | La base de la mayoría de los platos asiáticos es… | riz: 75%, chou: 5%, épice: 5%, other(s): 15% | arroz: 95%, picante: 5% | riz: 75%, soja: 10%, humide: 5%, other(s): 10% | riz: 60%, nouille: 10%, curry: 5%, other(s): 25% |
| 147 | In rock music, he likes the guitar and the bass, but his favorite instrument is still… | Dans la musique rock, il aime la guitare et la basse, mais son instrument préféré reste… | En la música rock, le gusta la guitarra y el bajo, pero su instrumento favorito sigue siendo… | batterie: 85%, piano: 5%, saxophone: 5%, synthétiseur: 5% | batería: 85%, piano: 10%, teclado: 5% | batterie: 90%, piano: 5%, tambour: 5% | batterie: 45%, tambour: 35%, piano: 10%, other(s): 10% |
| 148 | At the end of the movie, the prince approaches the princess and gives her… | À la fin du film, le prince s'approche de la princesse et lui donne… | Al final de la película, el príncipe se acerca a la princesa y le da… | baiser: 75%, rose: 10%, bague: 5%, other(s): 10% | beso: 95%, gracias: 5% | bisou: 60%, baiser: 15%, bise: 10%, other(s): 15% | baiser: 45%, bisou: 25%, bague: 10%, other(s): 20% |
| 149 | He really likes whisky, so we gave him… | Il aime beaucoup le whisky, c'est pourquoi nous lui avons offert… | A él le gusta mucho el whisky, por eso le regalamos… | bouteille: 75%, whisky: 10%, jameson: 5%, other(s): 10% | botella: 90%, año: 5%, juego: 5% | bouteille: 45%, verre: 30%, NV: 10%, other(s): 15% | bouteille: 75%, verre: 10%, whisky: 10%, vacances: 5% |
| 150 | Iván/Ivan was tired, and to wake up he drank… | Ivan était fatigué, et pour se réveiller, il a bu… | Iván estaba cansado, y para despertar se tomó… | café: 65%, eau: 10%, verre: 10%, other(s): 15% | café: 85%, taza: 5%, tiempo: 5%, vaso: 5% | café: 90%, boisson: 5%, redbull: 5% | café: 70%, eau: 20%, boisson: 5%, verre: 5% |
| 151 | Her boyfriend is a musician, and for their anniversary he wrote her… | Son petit ami est musicien, et pour son anniversaire, il lui a écrit… | Su novio es músico, y por su aniversario le ha escrito… | chanson: 85%, partition: 5%, poème: 5%, sonate: 5% | canción: 90%, poema: 5%, tema: 5% | chanson: 80%, dédicace: 5%, lettre: 5%, other(s): 10% | chanson: 90%, carte: 5%, poème: 5% |
| 152 | For having stolen several things in a store, he will spend a year locked up in… | Pour avoir volé plusieurs choses dans un magasin, il passera un an enfermé dans… | Por haber robado varias cosas en una tienda, él pasará un año encerrado en… | prison: 75%, cellule: 20%, centre: 5% | cárcel: 70%, prisión: 25%, reformatorio: 5% | prison: 85%, carte: 5%, correctionnel: 5%, maison: 5% | prison: 75%, cellule: 5%, docteur: 5%, other(s): 15% |
| 153 | It is easier to practice a minority sport by joining… | Il est plus facile de pratiquer un sport minoritaire lorsqu'on s'inscrit dans… | Es más fácil practicar un deporte minoritario asociándose a… | club: 80%, athlétisme: 5%, école: 5%, other(s): 10% | club: 50%, NV: 10%, asociación: 10%, other(s): 30% | club: 35%, école: 10%, équipe: 10%, other(s): 45% | club: 40%, équipe: 25%, NV: 10%, other(s): 25% |
| 154 | If you are looking for Sonia, she is preparing the food, you will probably find her in… | Si vous cherchez Sonia, elle prépare le repas, vous la trouverez probablement dans… | Si buscan a Sonia, está preparando la comida, seguramente la encuentren en… | cuisine: 100% | cocina: 95%, casa: 5% | cuisine: 100% | cuisine: 100% |
| 155 | He was already wearing the suit, but he had to call his wife to help him tie… | Il avait déjà mis son costume, mais il a dû appeler sa femme pour qu'elle l'aide à mettre… | Ya tenía puesto el traje, pero tuvo que llamar a su mujer para que le ayudara a atarse… | cravate: 75%, noeud-papillon: 10%, perruque: 10%, pantalon: 5% | corbata: 80%, zapato: 15%, cordón: 5% | cravate: 55%, NV: 20%, chapeau: 10%, other(s): 15% | cravate: 60%, maquillage: 15%, chapeau: 10%, other(s): 15% |
| 156 | The symbol of Catholicism is… | Le symbole du catholicisme est… | El símbolo del catolicismo es… | croix: 95%, pape: 5% | cruz: 100% | croix: 95%, amour: 5% | croix: 70%, crucifix: 20%, NV: 5%, jesus: 5% |
| 157 | The stabbing murder took place in the kitchen, the presumed murder weapon is… | Le meurtre à arme blanche a eu lieu dans la cuisine, l'arme du crime est probablement… | El asesinato por arma blanca se produjo en la cocina, el arma homicida es presuntamente… | couteau: 90%, auteur: 5%, tiroir: 5% | cuchillo: 95%, NV: 5% | couteau: 90%, NV: 5%, assassinat: 5% | couteau: 95%, cuisinier: 5% |
| 158 | When he was little, before going to bed they always told him… | Quand il était petit, quand il allait au lit, on lui racontait toujours… | Cuando era pequeño, al irse a la cama siempre le contaban… | histoire: 100% | cuento: 100% | histoire: 50%, conte: 35%, nuit: 10%, NV: 5% | histoire: 75%, conte: 15%, fable: 5%, nuit: 5% |
| 159 | Billiards is a game and swimming is… | Le billard est un jeu et la natation est… | El billar es un juego y la natación es… | sport: 95%, activité: 5% | deporte: 100% | sport: 100% | sport: 95%, activité: 5% |
| 160 | Of the four elements, water is the one that best puts out… | Des quatre éléments, l'eau est celui qui éteint le mieux… | De los cuatro elementos, el agua es el que mejor apaga… | feu: 95%, organisme: 5% | fuego: 85%, incendio: 10%, sed: 5% | feu: 60%, considération: 5%, fou: 5%, other(s): 30% | feu: 85%, NV: 10%, froid: 5% |
| 161 | Before cooking the chicken, you have to preheat… | Avant de cuire le poulet, il faut préchauffer… | Antes de cocinar el pollo, hay que precalentar… | four: 95%, micro-onde: 5% | horno: 80%, aceite: 5%, olla: 5%, other(s): 10% | four: 85%, eau: 10%, feu: 5% | four: 90%, cuisiniere: 5%, feu: 5% |
| 162 | Marc hoped that if he did good things for others, after dying he would end up in… | Marc espérait que s'il faisait de bonnes choses pour les autres, après sa mort, il atteindrait… | Marc esperaba que, si hacía cosas buenas por los demás, después de morir acabaría en… | paradis: 90%, nirvana: 5%, paix: 5% | cielo: 95%, paraíso: 5% | ciel: 30%, paradis: 20%, reconnaissance: 10%, other(s): 40% | paradis: 60%, NV: 10%, ciel: 10%, other(s): 20% |
| 163 | The lawyer did not agree with the sentence that had been pronounced by… | L'avocat n'était pas d'accord avec la sentence qu'avait prononcé… | El abogado no estaba de acuerdo con la sentencia que había dictado… | juge: 90%, jury: 5%, procureur: 5% | juez: 95%, tribunal: 5% | juge: 90%, NV: 5%, proces: 5% | juge: 75%, NV: 5%, appel: 5%, other(s): 15% |
| 164 | The house is locked, to open the main door they needed… | La maison est fermée, pour ouvrir la porte d'entrée il faudra leur donner… | La casa está cerrada, para abrir la puerta principal necesitaron… | clé: 85%, double: 10%, code: 5% | llave: 75%, cerrajero: 10%, NV: 5%, other(s): 10% | clé: 95%, coup: 5% | clé: 85%, mot: 10%, appel: 5% |
| 165 | To light the cigarette he used… | Pour allumer sa cigarette, il a utilisé… | Para encender el cigarrillo él usó… | briquet: 90%, allumette: 5%, zippo: 5% | mechero: 80%, encendedor: 15%, cerilla: 5% | briquet: 55%, feu: 25%, match: 15%, allumette: 5% | briquet: 80%, allumette: 10%, feu: 5%, match: 5% |
| 166 | After the accident, he couldn’t remember anything because he had lost… | Après l'accident, il ne se souvenait de rien parce qu'il avait complètement perdu… | Después del accidente él no podía recordar nada porque había perdido… | memoire: 90%, connaissance: 10% | memoria: 95%, conocimiento: 5% | memoire: 90%, conscience: 5%, orientation: 5% | memoire: 100% |
| 167 | Esther’s/Mia’s father likes to stay informed, so every day he reads… | Le père de Mia aime se tenir au courant de l'actualité, c'est pourquoi chaque jour il lit… | Al padre de Esther le gusta estar al tanto de la información, por eso, cada día lee… | journal: 90%, info: 5%, nouvelle: 5% | periódico: 95%, diario: 5% | journal: 80%, nouvelle: 10%, courant: 5%, magasin: 5% | journal: 80%, NV: 15%, nouvelle: 5% |
| 168 | He loved classical music and had very long fingers, so he decided to learn to play… | Il aimait la musique classique et avait de très longs doigts, il a donc décidé d'apprendre… | Le encantaba la música clásica y tenía los dedos muy largos, así que decidió aprender a tocar… | piano: 90%, musique: 5%, violon: 5% | piano: 80%, violín: 10%, guitarra: 5%, música: 5% | piano: 85%, violon: 10%, violoncelle: 5% | piano: 90%, guitare: 5%, violon: 5% |
| 169 | In summer it is important to protect yourself, the sun can cause injuries to… | En été, il est important de se protéger, le soleil peut abîmer… | En verano es importante protegerse, el sol puede causar lesiones en… | peau: 90%, yeux: 10% | piel: 100% | peau: 85%, yeux: 10%, NV: 5% | peau: 95%, yeux: 5% |
| 170 | His brother fell while skiing and broke… | Son frère est tombé au ski et s'est cassé… | Su hermano se cayó esquiando y se rompió… | jambe: 55%, poignet: 15%, bras: 5%, other(s): 25% | pierna: 60%, tibia: 10%, tobillo: 10%, other(s): 20% | jambe: 70%, bras: 15%, genou: 5%, other(s): 10% | jambe: 80%, bras: 10%, automne: 5%, genou: 5% |
| 171 | In ancient Egypt, to honor each Pharaoh who died, they built… | Dans l'Egypte ancienne, pour honorer chaque pharaon qui mourait, on construisait… | En el antiguo Egipto, para honrar a cada Faraón que moría, construían… | pyramide: 70%, tombeau: 15%, nécropole: 5%, other(s): 10% | pirámide: 95%, mausoleo: 5% | pyramide: 85%, chat: 5%, sarcophage: 5%, tombe: 5% | pyramide: 90%, sarcophage: 5%, temple: 5% |
| 172 | In summer Carlos/Charles bathes at his neighbors’ house, who have… | En été, Charles se baigne chez ses voisins qui ont… | En verano Carlos se baña en casa de sus vecinos, que tienen… | piscine: 100% | piscina: 95%, jardín: 5% | piscine: 85%, NV: 5%, an: 5%, baignoire: 5% | piscine: 95%, pêcheur: 5% |
| 173 | The girl weighs nothing, she is light as… | La fille ne pèse rien, elle est légère comme… | La niña no pesa nada, es ligera como… | plume: 100% | pluma: 90%, aire: 5%, papel: 5% | plume: 75%, fleur: 5%, mère: 5%, other(s): 15% | plume: 80%, feuille: 10%, bâton: 5%, souris: 5% |
| 174 | On Thursday is his brother’s birthday, they have to buy him… | Jeudi, c'est l'anniversaire de son frère, il faut lui acheter… | El jueves es el cumpleaños de su hermano, tienen que comprarle… | cadeau: 95%, gâteau: 5% | regalo: 90%, juego: 5%, psp: 5% | cadeau: 90%, gâteau: 10% | cadeau: 95%, football: 5% |
| 175 | Before sending a letter from the post office, you have to put… | Avant d'envoyer une lettre par la poste, il faut mettre… | Antes de enviar una carta desde la oficina postal, hay que poner… | timbre: 100% | sello: 90%, dirección: 10% | timbre: 75%, NV: 5%, adresse: 5%, other(s): 15% | timbre: 70%, cachet: 10%, tampon: 10%, other(s): 10% |
| 176 | At that time there was no public transport, so he had to call… | A cette heure-ci il n'y avait plus de transports publics, elle a donc dû appeler… | A esa hora ya no había transporte urbano, así que tuvo que llamar a… | taxi: 95%, ami: 5% | taxi: 90%, servicio: 5%, uber: 5% | taxi: 95%, uber: 5% | taxi: 90%, frère: 5%, mère: 5% |
| 177 | At breakfast he served the toast on a plate and the coffee in… | Au petit-déjeuner, il a présenté les tartines dans une assiette et le café dans… | En el desayuno él presentó las tostadas en un plato y el café en… | tasse: 75%, bol: 10%, mug: 5%, other(s): 10% | taza: 100% | tasse: 65%, pot: 15%, NV: 10%, other(s): 10% | tasse: 80%, pot: 10%, bol: 5%, ville: 5% |
| 178 | After dinner, to watch the news, he turns on… | Après le dîner, pour regarder les informations, il allume… | Después de cenar, para ver las noticias, enciende… | télévision: 100% | televisión: 95%, televisor: 5% | télévision: 85%, lumière: 10%, téléviseur: 5% | télévision: 85%, cigarette: 5%, lumière: 5%, pièce: 5% |
| 179 | On the map the pirates had, the cross indicated where… | Sur la carte que possédaient les pirates, la croix indiquait où se trouvait… | En el mapa que tenían los piratas la cruz indicaba dónde estaba… | trésor: 100% | tesoro: 100% | trésor: 95%, île: 5% | trésor: 90%, secret: 5%, NV: 5% |
| 180 | The most famous iron monument in France is… | Le monument en fer le plus célèbre de France est… | El monumento de hierro más conocido de Francia es… | tour: 100% | torre: 100% | tour: 100% | tour: 90%, louvre: 5%, mardi: 5% |
| 181 | He was on the highway and to cross the mountain he had to go through… | Il était sur l'autoroute et pour traverser la montagne, il a dû passer par… | Él iba por la autopista y para cruzar la montaña tuvo que pasar por… | tunnel: 70%, chemin: 5%, col: 5%, other(s): 20% | túnel: 70%, carretera: 10%, control: 5%, other(s): 15% | tunnel: 50%, pont: 15%, rivière: 10%, other(s): 25% | tunnel: 45%, col: 10%, NV: 5%, other(s): 40% |
| 182 | Coming back from the gym, the boy had sweat so much he went to take… | En revenant du gymnase, le garçon avait tellement transpiré qu'il est allé prendre… | Al volver del gimnasio, el chico había sudado tanto que se fue a dar… | douche: 100% | ducha: 95%, baño: 5% | douche: 70%, eau: 10%, NV: 5%, other(s): 15% | douche: 70%, eau: 10%, bain: 5%, other(s): 15% |
| 183 | This winter there were many storms, the cabin’s roof was carried away by… | Cet hiver, il y a eu beaucoup de tempêtes, le toit de la cabane a été emporté par… | Este invierno ha habido muchas tormentas, el tejado de la cabaña se lo llevó… | vent: 85%, bourrasque: 5%, rafale: 5%, tornade: 5% | viento: 90%, vendaval: 10% | vent: 90%, pluie: 5%, rafale: 5% | vent: 100% |
| 184 | Óscar/Oscar is tired of having to remember where he left off in the book last time, he wants to buy… | Oscar en a assez de devoir se souvenir de l'endroit du livre oú il en était la dernière fois, il veut s'acheter… | Óscar está cansado de tener que recordar en qué parte del libro se quedó la última vez, quiere comprarse… | marque-page: 90%, étagère: 5%, pense-bête: 5% | marcapáginas: 65%, punto: 20%, marcador: 10%, separapáginas: 5% | NV: 25%, livre: 10%, souvenir: 10%, other(s): 55% | signet: 35%, marque-page: 30%, NV: 5%, other(s): 30% |
| 185 | When light shines directly into the eyes of people with light-coloured eyes, their … shrink | Chez les gens qui ont les yeux clairs, quand la lumière les frappe directement, on voit se rétrécir… | A las personas con ojos claros, cuando les da la luz directamente, se les encoge… | pupille: 75%, iris: 20%, yeux: 5% | pupila: 90%, iris: 10% | pupille: 45%, yeux: 30%, NV: 15%, other(s): 10% | pupille: 65%, NV: 10%, yeux: 10%, other(s): 15% |
| 186 | If he gets his shirt dirty he won’t be able to clean it, the pen he uses has… | S'il se tâche, il ne pourra pas laver son t-shirt, le stylo qu'il utilise a… | Si él se mancha no podrá limpiarse la camiseta, el bolígrafo que usa tiene… | encre: 70%, NV: 15%, fuite: 10%, trou: 5% | tinta: 100% | encre: 70%, NV: 20%, casse: 5%, erreur: 5% | encre: 55%, NV: 25%, bic: 5%, other(s): 15% |
| 187 | Since the man saw it was raining, he went back home to get… | Quand il a vu qu'il pleuvait, l'homme est rentré chez lui pour prendre… | Como el hombre vio que llovía, volvió a casa a coger… | parapluie: 90%, anorak: 5%, veste: 5% | paraguas: 95%, chubasquero: 5% | parapluie: 85%, course: 5%, parkas: 5%, refuge: 5% | parapluie: 85%, impermeable: 10%, main: 5% |
| 188 | He left the car lights on all night, so in the morning he had to recharge… | Il a laissé les phares de la voiture allumés toute la nuit, si bien qu'au matin il a dû recharger… | Él dejó las luces del coche encendidas toda la noche, así que por la mañana tuvo que recargar… | batterie: 95%, voiture: 5% | batería: 100% | batterie: 100% | batterie: 90%, NV: 5%, voiture: 5% |
| 189 | To fry potatoes, while peeling them, you can put … on the stove | Pour faire sauter les patates, pendant que quelqu'un les pèle, il peut mettre sur le feu… | Para freír patatas, mientras uno las pela, puede poner al fuego… | poêle: 75%, huîle: 10%, NV: 5%, other(s): 10% | aceite: 40%, sartén: 30%, NV: 25%, ajo: 5% | huîle: 25%, poêle: 25%, casserole: 20%, other(s): 30% | poêle: 25%, NV: 20%, casserole: 10%, other(s): 45% |
| 190 | The girl thought she had a fever, she kept checking her temperature with… | La fille pensait qu'elle avait de la fièvre, elle n'arrêtait pas de contrôler sa température avec… | La chica creía que tenía fiebre, no dejaba de controlarse la temperatura con… | thermomètre: 95%, main: 5% | termómetro: 95%, palma: 5% | thermomètre: 75%, médicament: 10%, NV: 5%, other(s): 10% | thermomètre: 95%, aspirine: 5% |
| 191 | He loves astronomy, so at night he always tries to see if there are stars in… | Il adore l'astronomie, donc la nuit il essaye toujours de voir s'il y a des étoiles dans… | Le encanta la astronomía, así que por la noche siempre intenta ver si hay estrellas en… | ciel: 100% | cielo: 85%, firmamento: 15% | ciel: 90%, NV: 5%, télescope: 5% | ciel: 95%, télescope: 5% |
| 192 | The sun came out but it had just been raining, she thinks that soon we will be able to see… | Le soleil est apparu mais il pleuvait il y a peu, elle pense que bientôt on pourra voir… | Ha salido el sol pero hace nada estaba lloviendo, ella cree que pronto se podrá ver… | arc-en-ciel: 90%, ciel: 5%, soleil: 5% | arcoiris: 90%, NV: 5%, valle: 5% | arc-en-ciel: 90%, ciel: 5%, pluie: 5% | arc-en-ciel: 70%, soleil: 15%, ciel: 5%, other(s): 10% |
| 193 | For their baby’s first birthday, they prepared a cake and made him blow… | Pour le premier anniversaire de leur bébé, ils ont préparé un gâteau et lui ont fait souffler… | Para el primer cumpleaños de su bebé, prepararon una tarta y le hicieron soplar… | bougie: 100% | vela: 100% | bougie: 75%, chandelle: 15%, ballon: 5%, voîle: 5% | bougie: 90%, NV: 10% |
| 194 | One of the basic ingredients of a brownie is… | L'un des ingrédients de base du brownie est… | Uno de los ingredientes básicos de un brownie es… | chocolat: 65%, farine: 20%, beurre: 15% | chocolate: 80%, harina: 20% | chocolat: 75%, farine: 15%, NV: 5%, beurre: 5% | chocolat: 55%, farine: 25%, sucre: 10%, other(s): 10% |
| 195 | Mercury, Venus, and Saturn are three planets that are found in… | Mercure, Vénus et Saturne sont trois planètes que l'on trouve dans… | Mercurio, Venus y Saturno son tres planetas que se encuentran en… | système: 90%, espace: 5%, univers: 5% | sistema: 95%, espacio: 5% | système: 55%, voie: 20%, NV: 5%, other(s): 20% | système: 55%, espace: 20%, univers: 10%, other(s): 15% |
| 196 | He has a migraine, he will take a pill to see if it calms… | Il a une migraine, il va prendre un cachet pour voir si cela atténu… | Tiene migraña, se tomará una pastilla para ver si le calma… | douleur: 90%, mal: 10% | dolor: 85%, NV: 5%, siesta: 5%, síntoma: 5% | douleur: 80%, NV: 10%, mal: 5%, souffrance: 5% | douleur: 80%, NV: 10%, symptôme: 10% |
| 197 | When driving and wanting to overtake a car, it is important to turn on… | Quand on conduit et que l'on veut doubler une voiture, il est important de mettre… | Al conducir y querer adelantar a un coche, es importante poner… | clignotant: 100% | intermitente: 90%, distancia: 5%, maniobra: 5% | clignotant: 50%, indicateur: 10%, NV: 10%, other(s): 30% | indicateur: 30%, clignotant: 20%, ceinture: 10%, other(s): 40% |
| 198 | After finishing the meal, he wanted something sweet and ordered… | Quand il a fini son repas, il voulait quelque chose de sucré, il a donc demandé… | Cuando terminó la comida, quiso algo dulce y pidió… | dessert: 35%, café: 10%, biscuit: 5%, other(s): 50% | postre: 50%, tarta: 25%, chocolate: 5%, other(s): 20% | dessert: 50%, gâteau: 15%, NV: 5%, other(s): 30% | dessert: 45%, glace: 15%, crêpe: 10%, other(s): 30% |
| 199 | There is a lot of traffic on the highway and ambulance sirens, probably there is… | Il y a beaucoup de bouchons sur l'autoroute, et des sirènes d'ambulance, il y a probablement… | Hay mucho atasco en la autopista y sirenas de ambulancia, probablemente hay… | accident: 100% | accidente: 90%, atasco: 5%, colisión: 5% | accident: 100% | accident: 90%, collision: 5%, retard: 5% |
| 200 | Hinduism, like Islam, is… | L'hindouisme, comme l'islam, est… | El hinduismo, como el Islam, es… | religion: 95%, NV: 5% | religión: 100% | religion: 100% | religion: 85%, NV: 10%, religieux: 5% |
| 201 | To put a nose on a snowman, children use… | Pour mettre un nez à un bonhomme de neige, les enfants utilisent… | Para ponerle nariz a un muñeco de nieve los niños usan… | carotte: 95%, balle: 5% | zanahoria: 100% | carotte: 100% | carotte: 100% |
| 202 | When the diver saw the tentacle, he quickly realized the animal under the rock was… | Lorsque le plongeur a vu le tentacule, il a rapidement compris que l'animal sous le rocher était… | Cuando el buceador vio el tentáculo, rápidamente se dio cuenta de que el animal debajo de la roca era… | pieuvre: 60%, poulpe: 35%, méduse: 5% | pulpo: 90%, calamar: 5%, medusa: 5% | pieuvre: 45%, poulpe: 20%, NV: 15%, other(s): 20% | pieuvre: 65%, poulpe: 20%, NV: 15% |
| 203 | Pedro/Pierre loves reading, and for his birthday his sister decided to give him… | Pierre adore lire et, pour son anniversaire, sa sœur a décidé de lui offrir… | A Pedro le encanta leer, y por su cumpleaños su hermana decidió regalarle… | livre: 70%, encyclopédie: 10%, chèque-cadeau: 5%, other(s): 15% | libro: 100% | livre: 90%, carte: 5%, liseuse: 5% | livre: 75%, achat: 5%, bibliotheque: 5%, other(s): 15% |
| 204 | When he filled out all the documents, Hugo had to give a contact name in case there was… | Lorsqu'il a rempli tous les documents, Hugo a du indiquer le nom d'une personne de contact au cas où il y aurait… | Cuando rellenó todos los documentos, Hugo tuvo que dar el nombre de una persona de contacto por si había… | problème: 40%, accident: 20%, urgence: 20%, other(s): 20% | problema: 40%, emergencia: 35%, imprevisto: 10%, other(s): 15% | accident: 30%, problème: 20%, NV: 10%, other(s): 40% | urgence: 45%, accident: 20%, émergence: 15%, other(s): 20% |
| 205 | No matter how well you drive, it is possible that one day you will have… | Peu importe que tu conduises bien, il est possible qu'un jour tu aies… | No importa cuánto bien conduzcas, es posible que algún día tengas… | accident: 90%, problème: 5%, souci: 5% | accidente: 90%, susto: 10% | accident: 90%, mal: 5%, milieu: 5% | accident: 80%, NV: 15%, retard: 5% |
| 206 | Julia/Julie saw smoke coming out of her neighbor’s window and immediately grabbed the phone to report that there was… | Julie a vu de la fumée sortir de la fenêtre de son voisin et a immédiatement décroché le téléphone pour signaler qu'il y avait… | Julia vio humo saliendo de la ventana de su vecino e inmediatamente cogió el teléfono para avisar de que había… | incendie: 55%, feu: 35%, début: 5%, problème: 5% | incendio: 70%, fuego: 30% | incendie: 55%, feu: 40%, maison: 5% | feu: 50%, incendie: 45%, loi: 5% |
| 207 | Eva had finally decided to buy a house after renting … for a year | Eva s'est enfin décidée à acheter une maison après avoir loué pendant un an… | Eva había decidido finalmente comprar una casa después de un año alquilando… | appartement: 80%, NV: 10%, garage: 5%, studio: 5% | piso: 70%, habitación: 15%, NV: 10%, apartamento: 5% | appartement: 65%, NV: 25%, locataire: 5%, studio: 5% | NV: 40%, appartement: 40%, ami: 5%, other(s): 15% |
| 208 | Instead of recording the album at home, the singer decided it would be better to record it in… | Au lieu d'enregistrer l'album chez lui, le chanteur a décidé qu'il serait mieux de l'enregistrer dans… | En lugar de grabar el álbum en su casa, el cantante decidió que sería mejor grabarlo desde… | studio: 100% | estudio: 90%, exterior: 5%, mirador: 5% | studio: 40%, étude: 25%, bureau: 10%, other(s): 25% | studio: 90%, NV: 5%, ville: 5% |
| 209 | Elena/Alice wanted to go to Enrique’s/Eric’s party, but she was still waiting to receive… | Alice voulait aller à la fête d'Eric, mais elle attendait toujours de recevoir… | Elena quería ir a la fiesta de Enrique, pero todavía estaba esperando recibir… | invitation: 65%, colis: 10%, carton: 5%, other(s): 20% | invitación: 70%, paquete: 10%, nota: 5%, other(s): 15% | invitation: 85%, ami: 5%, NV: 5%, paquet: 5% | invitation: 75%, cadeau: 10%, NV: 10%, bouquet: 5% |
| 210 | When he called Víctor/Victor, he wasn’t home, but his mother said he could leave… | Lorsqu'elle a appelé Victor, il n'était pas chez lui, mais sa mère lui a dit qu'elle pouvait laisser… | Cuando llamó a Víctor no estaba en casa, pero su madre dijo que podía dejar… | message: 90%, clé: 5%, mot: 5% | mensaje: 60%, recado: 35%, nota: 5% | message: 55%, NV: 5%, ami: 5%, other(s): 35% | message: 80%, cherche: 5%, chose: 5%, other(s): 10% |
| 211 | Because Juan/Rémi didn’t properly heal his wound, he got… | Comme Rémi n'a pas bien soigné sa blessure, il a eu… | Como Juan no curó su herida bien, tuvo… | infection: 70%, complication: 15%, déchirure: 5%, other(s): 10% | infección: 45%, médico: 25%, NV: 5%, other(s): 25% | infection: 60%, complication: 15%, NV: 5%, other(s): 20% | infection: 55%, cicatrice: 15%, NV: 10%, other(s): 20% |
| 212 | The motorcyclist rolled up the leather jacket sleeve to show… | Le motard a retroussé la manche de sa veste en cuir pour montrer… | El motorista se subió la manga de la chaqueta de cuero para enseñar… | tatouage: 80%, bras: 5%, écusson: 5%, other(s): 10% | tatuaje: 90%, bícep: 5%, reloj: 5% | tatouage: 55%, NV: 5%, bras: 5%, other(s): 35% | tatouage: 65%, NV: 15%, bécane: 5%, other(s): 15% |
| 213 | To swim in the public pool, children must enter accompanied by… | Pour se baigner dans une piscine publique, les enfants doivent entrer accompagnés par… | Para bañarse en la piscina pública, los niños deben entrar acompañados por… | adulte: 95%, parent: 5% | adulto: 90%, NV: 5%, padre: 5% | adulte: 55%, parent: 35%, NV: 5%, papas: 5% | adulte: 80%, parent: 20% |
| 214 | There are always ways to improve your technique, whether you’re an advanced tennis player or… | Il y a toujours des façons d'améliorer sa technique, que l'on soit un joueur de tennis confirmé ou… | Siempre hay formas de mejorar tu técnica, tanto si eres un jugador de tenis avanzado como… | débutant: 55%, NV: 15%, amateur: 15%, other(s): 15% | principiante: 55%, novato: 20%, NV: 15%, amateur: 10% | amateur: 30%, débutant: 30%, NV: 20%, other(s): 20% | amateur: 35%, débutant: 35%, novice: 10%, other(s): 20% |
| 215 | Lola wanted to try the sauce, so she opened the jar and dipped… | Lola voulait goûter la sauce, elle a donc ouvert le pot et a trempé… | Lola quería probar la salsa así que abrió el bote y mojó… | doigt: 60%, cuillère: 30%, bout: 5%, tartine: 5% | pan: 55%, dedo: 15%, patata: 10%, other(s): 20% | cuillère: 30%, pain: 25%, doigt: 20%, other(s): 25% | doigt: 45%, cuillère: 30%, pain: 5%, other(s): 20% |
| 216 | Carmen’s/Elise’s first client was a failure, but the second was… | Le premier client de Elise a été un échec, mais le second a été… | El primer cliente de Carmen fue un fracaso, pero el segundo fue… | succès: 70%, réussite: 20%, NV: 5%, plaisir: 5% | éxito: 70%, NV: 20%, maravilloso: 5%, triunfo: 5% | succès: 85%, NV: 10%, client: 5% | succès: 75%, NV: 10%, réussite: 10%, argent: 5% |
| 217 | The doctors didn’t let Pilar/Zoé walk after her surgery, so to move around she needed… | Les médecins ne voulaient pas laisser Zoé marcher après son opération, donc pour se déplacer il lui fallait… | Los doctores no dejaron que Pilar caminase después de su operación, así que para moverse necesitaba… | fauteuil: 60%, béquille: 20%, chaise: 15%, NV: 5% | silla: 55%, muleta: 35%, ayuda: 10% | chaise: 40%, fauteuil: 30%, autorisation: 10%, other(s): 20% | fauteuil: 70%, NV: 10%, chaise: 10%, other(s): 10% |
| 218 | If someone shouts surrounded by silent mountains, ... is produced | Si quelqu'un crie entouré de montagnes silencieuses, il se produit… | Si uno grita rodeado entre montañas silenciosas, se produce… | écho: 85%, avalanche: 10%, miraclé: 5% | eco: 95%, avalancha: 5% | écho: 90%, avalanche: 5%, bruit: 5% | écho: 80%, bruit: 10%, NV: 5%, bruyards: 5% |
| 219 | To be able to fish, the man put … at the end of the rod | Pour pêcher, l'homme a mis au bout de sa canne à pêche… | Para pescar, el hombre puso al final de la caña… | hameçon: 55%, appât: 20%, ver: 15%, other(s): 10% | cebo: 60%, anzuelo: 25%, gusano: 15% | ver: 30%, appât: 20%, poisson: 15%, other(s): 35% | appât: 40%, ver: 35%, eau: 5%, other(s): 20% |
| 220 | Walking down the street, they saw a finish line and balloons around it, so they thought there must have been… | En se promenant dans la rue, ils ont vu une ligne d'arrivée et des ballons autour, ils ont donc pensé qu'il devait y avoir… | Paseando por la calle, vieron una línea de meta y unos globos alrededor, así que pensaron que debía haber… | course: 55%, fête: 15%, marathon: 15%, other(s): 15% | carrera: 75%, maratón: 10%, campeonato: 5%, other(s): 10% | course: 20%, fête: 20%, marathon: 15%, other(s): 45% | course: 60%, NV: 10%, anniversaire: 5%, other(s): 25% |
| 221 | Coming back from the bar drunk and with a bleeding nose, his wife thought he had had… | Quand il est revenu du bar, ivre et avec le nez en sang, sa femme a supposé qu'il y avait eu… | Al volver del bar borracho y con la nariz sangrando, su mujer pensó que había tenido… | bagarre: 65%, altercation: 20%, accident: 5%, other(s): 10% | pelea: 95%, accidente: 5% | bagarre: 20%, lutte: 20%, NV: 15%, other(s): 45% | bagarre: 35%, combat: 35%, lutte: 15%, other(s): 15% |
| 222 | To walk calmly through the desert without getting tired, tourists can ride on… | Pour se promener tranquilement dans le désert sans se fatiguer, les touristes peuvent monter sur… | Para pasear tranquilamente por el desierto sin cansarse, los turistas se pueden subir a… | chameau: 75%, dromadaire: 15%, NV: 5%, rocher: 5% | camello: 80%, NV: 5%, coche: 5%, other(s): 10% | chameau: 80%, voiture: 10%, cheval: 5%, motocyclé: 5% | chameau: 75%, camel: 5%, dromadaire: 5%, other(s): 15% |
| 223 | All children know Little Red Riding Hood, who is… | Tous les enfants connaissent Le Petit Chaperon rouge, qui est… | Todos los niños conocen La Caperucita Roja, que es… | conte: 55%, NV: 15%, fable: 10%, other(s): 20% | cuento: 85%, fábula: 5%, niña: 5%, personaje: 5% | conte: 30%, histoire: 20%, fille: 15%, other(s): 35% | conte: 30%, histoire: 25%, fille: 15%, other(s): 30% |
| 224 | The work team tried to solve the problem for hours but couldn’t find… | L'équipe de travail a passé des heures à essayer de résoudre le problème, mais n'a pas réussi à trouver… | El equipo de trabajo estuvo intentando resolver el problema durante horas pero no consiguió dar con… | solution: 85%, problème: 10%, origine: 5% | solución: 75%, avería: 5%, clave: 5%, other(s): 15% | solution: 90%, NV: 5%, souci: 5% | solution: 90%, approche: 5%, problème: 5% |
| 225 | Having been born on the peninsula, Gloria/Adèle never got used to living in/on… | Etant née sur le continent, Adèle ne s'est jamais habituée à vivre sur… | Al haber nacido en la península, Gloria nunca se acostumbró a vivir en… | île: 70%, NV: 5%, bateau: 5%, other(s): 20% | isla: 75%, extranjero: 10%, canarias: 5%, other(s): 10% | île: 40%, ville: 15%, afrique: 5%, other(s): 40% | île: 30%, mer: 15%, bord: 10%, other(s): 45% |
| 226 | Lucía/Lucie decided she wanted to work on a cruise ship, even though she had never been on... | Lucie a décidé qu'elle voulait travailler sur un paquebot de croisière, même si elle n'avait jamais été sur… | Lucía decidió que quería trabajar en un crucero, y eso que nunca había estado en… | bateau: 85%, mer: 10%, eau: 5% | barco: 50%, mar: 25%, NV: 15%, other(s): 10% | bateau: 40%, mer: 35%, NV: 15%, other(s): 10% | bateau: 60%, NV: 15%, mer: 10%, other(s): 15% |
| 227 | Whenever Celia/Camille walked her dog, it would run after… | Chaque fois que Camille promenait son chien, il partait en courant après… | Siempre que Celia paseaba a su perro, éste salía corriendo detrás de… | chat: 40%, NV: 20%, chien: 10%, other(s): 30% | gato: 35%, perro: 20%, NV: 10%, other(s): 35% | chat: 30%, oiseaux: 20%, chien: 15%, other(s): 35% | chat: 25%, chien: 20%, NV: 15%, other(s): 40% |
| 228 | The first time Jesús/Adam saw the Pacific, he thought it was a lake, but his brother laughed and explained that it is… | La première fois que Adam a vu le Pacifique, il a cru que c´était un lac, mais son frère a rigolé et lui a expliqué que c'était… | La primera vez que Jesús vio el Pacífico pensó que era un lago, pero su hermano se rio y le explicó que es… | ocean: 90%, mer: 5%, réalité: 5% | océano: 85%, mar: 15% | ocean: 90%, mer: 10% | ocean: 55%, mer: 35%, NV: 5%, rivière: 5% |
| 229 | When the man was crossing the desert, he thought he saw an oasis but it was actually… | Alors que l'homme marchait dans le désert, il a cru voir une oasis, alors que c'était… | Cuando el hombre iba por el desierto, creyó ver un oasis pero se trataba de… | mirage: 85%, dune: 5%, flaque: 5%, sable: 5% | espejismo: 60%, ilusión: 15%, alucinación: 10%, other(s): 15% | illusion: 20%, mirage: 20%, sable: 15%, other(s): 45% | mirage: 55%, illusion: 10%, rêve: 10%, other(s): 25% |
| 230 | While Marta/Jade walked through the colorful gardens, she bent down to pick… | En se promenant dans les jardins si colorés, Jade s'est penchée pour cueillir… | Mientras Marta paseaba por los jardines tan coloridos, se agachó a coger… | fleur: 90%, fruit: 5%, marguerite: 5% | flor: 65%, hoja: 10%, rosa: 10%, other(s): 15% | fleur: 85%, fruit: 5%, rose: 5%, vue: 5% | fleur: 80%, NV: 5%, baie: 5%, other(s): 10% |
| 231 | Pablo/Mathias swears he comes from space but no one believes that he really is… | Mathias jure qu'il vient de l'espace, mais personne ne croit vraiment qu'il soit… | Pablo jura venir del espacio pero nadie se cree que él realmente sea… | extra-terrestre: 65%, alien: 25%, astronaute: 5%, NV: 5% | extraterrestre: 45%, alienígena: 35%, NV: 10%, astronauta: 10% | extra-terrestre: 35%, astronaute: 25%, NV: 15%, other(s): 25% | extra-terrestre: 60%, alien: 15%, NV: 10%, other(s): 15% |
| 232 | With his khaki pants, Hawaiian shirt, and camera, it was clear that Andrés/Jérome was… | Avec son pantalon kaki, sa chemise hawaïenne et son appareil photo, on voyait bien que Jérome était… | Con sus pantalones caqui, su camisa hawaiana y su cámara de fotos se podía ver muy bien que Andrés era… | touriste: 65%, vacancess: 25%, NV: 10% | turista: 65%, americano: 5%, fan: 5%, other(s): 25% | touriste: 65%, vacancess: 25%, NV: 10% | vacancess: 55%, touriste: 35%, NV: 10% |
| 233 | The little girl left Santa Claus some chocolate cookies and… | La petite fille a laissé au Père Noël des biscuits au chocolat et… | La pequeña le dejó a Papá Noel unas galletas con chocolate y… | verre: 40%, lait: 30%, bonbon: 10%, other(s): 20% | leche: 55%, vaso: 35%, avellana: 5%, copa: 5% | lait: 65%, verre: 15%, bonbon: 5%, other(s): 15% | lait: 45%, verre: 40%, carotte: 10%, NV: 5% |
| 234 | After cooking, since she doesn’t like washing dishes by hand, she loads… | Quand elle finit de cuisiner, comme elle n'aime pas laver les assiettes à la main, elle lance… | Al acabar de cocinar, como no le gusta lavar platos a mano, pone… | lave-vaisselle: 95%, machine: 5% | lavavajillas: 90%, lavadora: 5%, plato: 5% | lave-vaisselle: 70%, NV: 15%, assiette: 15% | lave-vaisselle: 35%, assiette: 20%, machine-a-laver: 20%, other(s): 25% |
| 235 | At dinner, he saw that his mother wasn’t eating the soup because she needed… | Au dîner, elle s'est aperçue que sa mère ne mangeait pas la soupe, c'est qu'il lui fallait… | En la cena veía que su madre no estaba comiendo la sopa, y es que necesitaba… | cuillère: 55%, paille: 10%, sel: 10%, other(s): 25% | cuchara: 70%, NV: 15%, sal: 10%, ayuda: 5% | cuillère: 45%, NV: 25%, sel: 10%, other(s): 20% | cuillère: 35%, NV: 30%, sel: 10%, other(s): 25% |
| 236 | On her trip to Africa, she wanted a photo of the king of the jungle; she was very happy when she finally found… | Lors de son voyage en Afrique, il voulait une photo du roi de la jungle, il a été très heureux de trouver enfin… | En su viaje a África quiso una foto del rey de la selva, se alegró mucho cuando por fin encontró… | lion: 70%, animal: 5%, babouin: 5%, other(s): 20% | león: 80%, asentamiento: 5%, manada: 5%, other(s): 10% | lion: 85%, NV: 10%, safari: 5% | lion: 95%, photo: 5% |
| 237 | Alba/Jeanne lined up the nail with the others and, once satisfied, asked someone to pass her… | Jeanne a aligné le clou avec les autres et, lorsqu'elle a été satisfaite, elle a demandé qu'on lui passe… | Alba alineó el clavo con los otros y cuando estaba satisfecha pidió que le pasaran… | marteau: 85%, cadre: 5%, tableau: 5%, téléphone: 5% | martillo: 90%, alicates: 5%, cuadro: 5% | marteau: 80%, NV: 10%, outil: 5%, prix: 5% | marteau: 55%, NV: 20%, ami: 5%, other(s): 20% |
| 238 | The snow had piled up and they couldn’t leave the house, so to clear the way, David/Mathis quickly went to get… | La neige s'était accumulée et ils ne pouvaient pas sortir de la maison, alors pour dégager le chemin, Mathis est rapidement allé chercher… | La nieve se había acumulado y no podían salir de casa, así que para abrir camino, David rápidamente fue a buscar… | pelle: 100% | pala: 100% | pelle: 100% | pelle: 60%, bêche: 10%, aide: 5%, other(s): 25% |
| 239 | The backpack got stuck every time he opened or closed it, it was time to change… | Ça coinçait chaque fois qu'elle ouvrait ou fermait le sac à dos, il était temps de changer… | Se atascaba cada vez que abría o cerraba la mochila, era momento de cambiar… | fermeture: 70%, sac: 25%, équipement: 5% | cremallera: 75%, mochila: 15%, NV: 10% | fermeture: 30%, sac: 30%, zip: 15%, other(s): 25% | fermeture: 35%, sac: 30%, zip: 15%, other(s): 20% |
| 240 | When he was almost finishing the puzzle after so many weeks, he realized he was missing… | Alors qu'il était sur le point de terminer le puzzle après des semaines, il s'est rendu compte qu'il lui manquait… | Cuando casi iba a acabar el puzzle después de tantas semanas, él se dio cuenta de que le faltaba… | pièce: 100% | pieza: 100% | pièce: 95%, partie: 5% | pièce: 75%, morceau: 20%, billet: 5% |
| 241 | After showing them the cellar, he offered them to take home… | Après leur avoir montré la cave, il leur a offert de rapporter chez eux… | Después de enseñarle la bodega, les ofreció llevarse a casa… | bouteille: 80%, vin: 20% | botella: 65%, vino: 25%, par: 10% | bouteille: 35%, vin: 20%, voiture: 20%, other(s): 25% | bouteille: 30%, NV: 20%, vin: 20%, other(s): 30% |
| 242 | Before going to sleep, he always brushes his teeth and puts on… | Avant d'aller se coucher, il se brosse toujours les dents et enfile… | Antes de irse a dormir, él siempre se lava los dientes y se pone… | pyjama: 95%, pantalon: 5% | pijama: 55%, mascarilla: 15%, antifaz: 10%, other(s): 20% | pyjama: 50%, crème: 10%, lit: 10%, other(s): 30% | pyjama: 80%, fil: 5%, ongle: 5%, other(s): 10% |
| 243 | His father remained standing, but he quickly went to get him… | Son père restait debout, mais il est rapidement allé lui chercher… | El padre de él se quedó de pie, pero rápidamente fue a buscarle… | chaise: 80%, tabouret: 10%, eau: 5%, verre: 5% | silla: 45%, NV: 35%, colegio: 10%, other(s): 10% | chaise: 40%, NV: 5%, chose: 5%, other(s): 50% | chaise: 55%, NV: 10%, aide: 5%, other(s): 30% |
| 244 | He had been hearing a buzzing flying around the house for a while, he thought it must be… | Ça faisait un moment qu'il entendait un bourdonnement dans la maison, il pensait que ce devait être… | Llevaba rato escuchando un zumbido volando por casa, pensó que debía ser… | abeille: 20%, bourdon: 15%, mouche: 15%, other(s): 50% | abeja: 30%, mosquito: 30%, mosca: 25%, other(s): 15% | abeille: 25%, mouche: 20%, voleur: 10%, other(s): 45% | abeille: 30%, mouche: 25%, bourdon: 10%, other(s): 35% |
| 245 | For Valentine’s Day, her boyfriend bought her a big box of chocolates and… | Pour la Saint-Valentin, son fiancé lui a acheté une grosse boîte de chocolats et… | Por San Valentín, su novio le compró una gran caja de bombones y… | bouquet: 35%, fleur: 35%, rose: 10%, other(s): 20% | flor: 30%, ramo: 30%, rosa: 30%, other(s): 10% | rose: 35%, fleur: 25%, bonbon: 10%, other(s): 30% | fleur: 55%, bouquet: 15%, rose: 15%, other(s): 15% |
| 246 | He didn’t have time to finish the task on Thursday, so he told his boss he would finish it… | Il n'a pas eu le temps de terminer le travail le jeudi, il a donc dit à son chef qu'il le terminerait… | No le dio tiempo a terminar la tarea el jueves, así que él le dijo a su jefe que lo terminaría… | vendredi: 55%, lendemain: 25%, demain: 10%, other(s): 10% | viernes: 55%, dia: 15%, lunes: 10%, other(s): 20% | vendredi: 65%, demain: 15%, NV: 5%, other(s): 15% | vendredi: 55%, demain: 25%, lendemain: 15%, contrat: 5% |
| 247 | To go camping they needed a place to sleep, so they bought… | Pour aller camper, ils avaient besoin d'un endroit pour dormir, ils ont donc acheté… | Para irse de acampada necesitaban un sitio donde dormir, así que compraron… | tente: 95%, hôtel: 5% | tienda: 95%, saco: 5% | tente: 70%, sac: 30% | tente: 90%, sac: 10% |
| 248 | In the coldest regions, like England, all houses have … on the floor | Dans les régions plus froides, comme l'Angleterre, toutes les maisons ont au sol… | En las regiones más frías, como Inglaterra, todas las casas tienen en el suelo… | chauffage: 20%, tapis: 20%, parquet: 15%, other(s): 45% | moqueta: 65%, aclimatado: 5%, alfombra: 5%, other(s): 25% | tapis: 40%, NV: 15%, moquette: 15%, other(s): 30% | tapis: 55%, isolation: 10%, moquette: 10%, other(s): 25% |
| 249 | They were tied, but right in the last minute the visiting team won thanks to… | Les deux équipes étaient à égalité, mais à la dernière minute, l'équipe visiteuse a gagné grâce à… | Iban empatando, pero justo en el último minuto el equipo visitante ganó gracias a… | but: 50%, penalty: 20%, action: 5%, other(s): 25% | gol: 55%, penalti: 20%, canasta: 5%, other(s): 20% | but: 60%, NV: 10%, joueur: 10%, other(s): 20% | but: 40%, objectif: 10%, penalty: 10%, other(s): 40% |
| 250 | Luís/Simon felt bad for what he said to her and knew he owed her… | Simon se sentait mal pour ce qu'il lui avait dit et savait qu'il lui devait … | Luís se sentía mal por lo que le dijo a ella y sabía que le debía… | excuse: 80%, NV: 10%, argent: 5%, câlin: 5% | disculpa: 60%, perdón: 20%, explicación: 10%, other(s): 10% | NV: 30%, excuse: 25%, pardon: 25%, other(s): 20% | excuse: 55%, NV: 30%, desolee: 5%, other(s): 10% |
| 251 | They created a football team in the neighborhood, but to learn the basics, they needed… | Ils ont créé une équipe de football dans le quartier, mais pour apprendre les bases, il leur fallait… | Crearon un equipo de fútbol en el barrio, pero para aprender la base, necesitaban… | entraîneur: 55%, coach: 20%, ballon: 5%, other(s): 20% | entrenador: 85%, balón: 5%, norma: 5%, regla: 5% | NV: 30%, coach: 25%, entraîneur: 25%, other(s): 20% | entraîneur: 50%, NV: 15%, coach: 15%, other(s): 20% |
| 252 | To pay for university tuition, their parents asked for… | Pour pouvoir payer les frais d'inscription à l'université, ses parents ont demandé… | Para poder pagar la matrícula de la universidad, sus padres pidieron… | prêt: 65%, aide: 15%, argent: 5%, other(s): 15% | préstamo: 85%, crédito: 15% | prêt: 40%, crédit: 35%, bourse: 10%, other(s): 15% | prêt: 35%, bourse: 10%, facture: 10%, other(s): 45% |
| 253 | The ocean liner began to sink after crashing into… | Le paquebot a commencé à couler après avoir heurté… | El transatlántico se empezó a hundir después de chocar con… | iceberg: 80%, récif: 10%, glacier: 5%, rocher: 5% | iceberg: 90%, barco: 5%, bloque: 5% | iceberg: 65%, rocher: 15%, pierre: 10%, other(s): 10% | iceberg: 60%, rocher: 10%, NV: 5%, other(s): 25% |
| 254 | To hang the painting, he only needed a hammer and… | Pour accrocher le tableau, il lui fallait uniquement un marteau et… | Para colgar el cuadro él solo necesitaba un martillo y… | clou: 90%, vis: 10% | clavo: 90%, alcayata: 5%, escalera: 5% | clou: 40%, ongle: 15%, NV: 10%, other(s): 35% | clou: 70%, vis: 10%, NV: 5%, other(s): 15% |
| 255 | After the argument, Ana went to her room and closed… | Après la dispute, Marion est allée dans sa chambre et a fermé… | Después de la discusión, Ana se fue a su habitación y cerró… | porte: 95%, clé: 5% | puerta: 85%, portazo: 10%, pestillo: 5% | porte: 100% | porte: 100% |
| 256 | Grandpa cut the cheese with… | Le grand-père a coupé le fromage avec… | El abuelo cortó el queso con… | couteau: 90%, fil: 5%, opinel: 5% | cuchillo: 80%, navaja: 15%, esmero: 5% | couteau: 100% | couteau: 100% |
| 257 | One year after the death, Rosa/Margot visited… | Un an après le décès, Margot est allée voir… | Un año después de la muerte, Rosa visitó… | tombe: 75%, psychologue: 10%, cimetière: 5%, other(s): 10% | tumba: 70%, cementerio: 25%, mausoleo: 5% | tombe: 35%, tombeau: 20%, mari: 10%, other(s): 35% | tombe: 40%, cimetière: 10%, médium: 10%, other(s): 40% |
| 258 | The mother asked him to turn of …, before going to bed | La mère lui a demandé que lorsqu'elle irait au lit, elle éteigne d'abord… | La madre le pidió que cuando se fuese a la cama apagase primero… | lumière: 75%, téléphone: 10%, télévision: 10%, lampe: 5% | luz: 60%, televisión: 20%, móvil: 5%, other(s): 15% | lumière: 85%, NV: 5%, dent: 5%, télévision: 5% | lumière: 80%, NV: 5%, lampe: 5%, other(s): 10% |
| 259 | Just when the whole movie theater was silent ... began to sound | Alors que toute la salle de cinéma était silencieuse, s'est mis à sonner… | Justo cuando toda la sala de cine estaba en silencio, comenzó a sonar… | téléphone: 75%, alarme: 10%, portable: 10%, glas: 5% | móvil: 35%, teléfono: 15%, banda: 10%, other(s): 40% | téléphone: 65%, alarme: 10%, bande: 5%, other(s): 20% | téléphone: 35%, NV: 15%, fort: 15%, other(s): 35% |
| 260 | After many days, the chicks were finally ready to leave… | Après de nombreux jours, les oisillons étaient enfin prêts à quitter… | Después de muchos días, los polluelos finalmente estaban preparados para abandonar… | nid: 100% | nido: 100% | nid: 70%, œuf: 10%, cocon: 5%, other(s): 15% | nid: 80%, pays: 10%, mère: 5%, œuf: 5% |
| 261 | Given such cold weather outside his house in the mountains, it was time to light... | Avec le temps si froid à l'extérieur de leur maison en montagne, il était temps d'allumer… | Con el tiempo tan frío fuera de su casa en la montaña, era hora de encender… | chauffage: 30%, cheminée: 30%, feu: 30%, poêle: 10% | chimenea: 60%, calefacción: 10%, hoguera: 10%, other(s): 20% | cheminée: 60%, chauffage: 25%, feu: 15% | feu: 50%, cheminée: 15%, bougie: 10%, other(s): 25% |
| 262 | Emma had to pull off to the side of the road because she had a flat… | Emma a dû se ranger sur le côté de la route car elle avait crevé… | Emma tuvo que apartarse a un lado de la carretera ya que se le había pinchado… | pneu: 90%, roue: 10% | rueda: 90%, llanta: 5%, neumático: 5% | roue: 30%, voiture: 25%, pneu: 15%, other(s): 30% | pneu: 50%, NV: 15%, voiture: 15%, other(s): 20% |
| 263 | To learn about their distant ancestors, they collected information and drew… | Pour en savoir plus sur leurs ancêtres les plus lointains, ils ont recueilli des informations et dessiné… | Para aprender sobre sus ancestros más lejanos, recopilaron información y dibujaron… | arbre: 65%, croquis: 10%, carte: 5%, other(s): 20% | árbol: 95%, pared: 5% | arbre: 75%, NV: 5%, carte: 5%, other(s): 15% | arbre: 65%, photo: 10%, diagramme: 5%, other(s): 20% |
| 264 | Sandra put the flowers she had bought in… | Sandra a mis les fleurs qu'elle avait achetées dans… | Sandra puso las flores que había comprado en… | vase: 100% | floristería: 35%, jarrón: 35%, florero: 20%, agua: 10% | vase: 35%, fleuriste: 20%, pot: 10%, other(s): 35% | vase: 75%, fleuriste: 10%, eau: 5%, other(s): 10% |
| 265 | Sara/Sarah saw animals from all over the world in… | Sarah a vu des animaux du monde entier dans… | Sara vio animales de todas las partes del mundo en… | zoologique: 55%, documentaire: 20%, livre: 15%, other(s): 10% | zoológico: 90%, viaje: 10% | zoologique: 60%, télévision: 25%, cirque: 5%, other(s): 10% | zoologique: 85%, afrique: 5%, internet: 5%, vie: 5% |
| 266 | The university student went to the library to read… | L'étudiante universitaire est allée à la bibliothèque pour lire… | La estudiante universitaria fue a la biblioteca para leer… | livre: 60%, encyclopédie: 10%, étude: 10%, other(s): 20% | libro: 55%, apuntes: 10%, NV: 5%, other(s): 30% | livre: 85%, bible: 5%, journal: 5%, note: 5% | livre: 90%, NV: 5%, manuel: 5% |
| 267 | For a plant to grow, first you have to plant… | Pour faire pousser une plante, il faut d'abord planter… | Para que crezca una planta primero hay que plantar… | graine: 90%, plante: 10% | semilla: 100% | graine: 60%, NV: 15%, fruit: 5%, other(s): 20% | graine: 80%, NV: 5%, plante: 5%, other(s): 10% |
| 268 | Surfers are usually afraid of being bitten by… | Les surfeurs ont souvent peur d'être mordus par… | Los surferos suelen tener miedo de ser mordidos por… | requin: 95%, méduse: 5% | tiburon: 100% | requin: 75%, NV: 5%, animal: 5%, other(s): 15% | requin: 65%, vague: 20%, bête: 5%, other(s): 10% |
| 269 | At dinner, she cut her food with… | Pendant le dîner, il a coupé sa nourriture avec… | En la cena, cortó su comida con… | couteau: 90%, eau: 5%, verre: 5% | cuchillo: 90%, cubierto: 5%, NV: 5% | couteau: 95%, viande: 5% | couteau: 95%, fourchette: 5% |
| 270 | To pay for the car, Clara simply wrote… | Pour payer la voiture, Clara a simplement signé… | Para pagar el coche, Clara simplemente escribió… | chèque: 50%, prêt: 25%, contrat: 10%, other(s): 15% | cheque: 55%, padre: 15%, número: 10%, other(s): 20% | chèque: 30%, contrat: 30%, crédit: 15%, other(s): 25% | contrat: 45%, chèque: 25%, accord: 15%, other(s): 15% |
| 271 | To prepare fried eggs, she first poured oil into… | Pour préparer des œufs frits, il a d'abord versé de l'huile dans… | Para preparar unos huevos fritos, primero echó aceite en… | poêle: 95%, casserole: 5% | sartén: 100% | poêle: 70%, casserole: 25%, panneau: 5% | poêle: 75%, casserole: 15%, NV: 10% |
| 272 | During his long sea voyage, the pirate grew… | Pendant son long voyage en bateau, le pirate s'est vu pousser… | Durante su largo viaje en barco, al pirata le creció… | barbe: 70%, aile: 15%, bout: 5%, other(s): 10% | barba: 95%, pelo: 5% | mer: 25%, barbe: 15%, NV: 10%, other(s): 50% | mer: 25%, NV: 15%, barbe: 15%, other(s): 45% |
| 273 | To keep the dogs away from the garden, they put up... | Pour empêcher les chiens de passer dans le jardin, ils ont construit… | Para evitar que los perros pasasen al jardín, pusieron… | clôture: 35%, barrière: 15%, barrage: 10%, other(s): 40% | valla: 65%, verja: 20%, cercado: 5%, other(s): 10% | clôture: 35%, mur: 30%, maison: 10%, other(s): 25% | clôture: 55%, mur: 10%, bac à litière: 5%, other(s): 30% |
| 274 | She wanted her wishes to come true, so she asked the witch to prepare ... in her pot | Elle voulait que ses souhaits se réalisent, alors elle a demandé à la sorcière de préparer dans son chaudron… | Quería que sus deseos se hiciesen realidad, así que ella pidió a la bruja que le preparase en su olla… | potion: 90%, breuvage: 5%, sort: 5% | poción: 55%, pócima: 25%, brebaje: 10%, other(s): 10% | potion: 60%, NV: 20%, chose: 5%, other(s): 15% | potion: 80%, NV: 10%, sort: 10% |
| 275 | After winter, the heat increases in spring and melts… | Après l'hiver, la chaleur augmente au printemps, et commence à fondre… | Después del invierno, aumenta el calor en primavera y se derrite… | neige: 90%, été: 5%, glace: 5% | nieve: 65%, hielo: 35% | neige: 80%, glace: 20% | neige: 60%, glace: 25%, arbre: 5%, other(s): 10% |
| 276 | He knew he needed a new sock when he saw it had… | Il savait qu'il avait besoin d'une nouvelle chaussette quand il a vu qu'elle avait… | Él sabía que necesitaba un calcetín nuevo cuando vio que tenía… | trou: 90%, NV: 5%, rien: 5% | agujero: 80%, tomate: 15%, NV: 5% | trou: 80%, chaussette: 10%, casse: 5%, defaut: 5% | trou: 80%, chaussette: 10%, NV: 5%, mache: 5% |
| 277 | The janitor accidentally spilled some water on… | Le concierge a malencontreusement renversé un peu d'eau sur… | El conserje derramó sin querer un poco de agua en… | sol: 15%, tapis: 15%, courrier: 10%, other(s): 60% | suelo: 55%, pasillo: 15%, entrada: 5%, other(s): 25% | sol: 50%, bureau: 10%, table: 10%, other(s): 30% | sol: 60%, tapis: 10%, NV: 5%, other(s): 25% |
| 278 | The house fire happened because someone forgot to turn off… | L'incendie de maison s'est produit pour avoir oublié d'éteindre… | El incendio en casa sucedió por haber olvidado apagar… | gaz: 30%, four: 15%, cheminée: 10%, other(s): 45% | estufa: 15%, horno: 15%, chimenea: 10%, other(s): 60% | feu: 25%, four: 25%, bougie: 20%, other(s): 30% | bougie: 35%, cuisiniere: 10%, feu: 10%, other(s): 45% |
| 279 | During the best part of his guitar performance, Marcos/Luc broke… | Au meilleur moment de sa performance à la guitare, Luc a cassé… | En la mejor parte de su actuación de guitarra, a Marcos se le rompió… | corde: 95%, vase: 5% | cuerda: 95%, púa: 5% | corde: 85%, guitare: 5%, main: 5%, pleurer: 5% | corde: 55%, guitare: 20%, chaine: 10%, other(s): 15% |
| 280 | The woman’s shirt was so old and worn that it had… | La chemise de la femme était si vieille et usée qu'elle avait… | La camisa de la mujer estaba tan vieja y usada que tenía… | trou: 95%, couleur: 5% | agujero: 55%, NV: 5%, bola: 5%, other(s): 35% | trou: 55%, NV: 10%, tache: 10%, other(s): 25% | trou: 45%, NV: 30%, chemise: 10%, other(s): 15% |
| 281 | The short driver had to adjust… | Le conducteur de petite taille devait ajuster… | El conductor bajito tenía que ajustar… | siège: 75%, hauteur: 15%, fauteuil: 5%, volant: 5% | asiento: 85%, altura: 5%, cinturón: 5%, espejo: 5% | siège: 45%, hauteur: 15%, ceinture: 10%, other(s): 30% | siège: 50%, NV: 15%, chaise: 5%, other(s): 30% |
| 282 | The rural real estate agency quickly sold… | L'agence immobilière rurale a rapidement vendu… | La agencia inmobiliaria rural rápidamente vendió… | maison: 60%, NV: 10%, appartement: 10%, other(s): 20% | casa: 55%, piso: 15%, cortijo: 5%, other(s): 25% | maison: 55%, ferme: 15%, appartement: 5%, other(s): 25% | maison: 75%, ferme: 10%, produit: 5%, other(s): 10% |
| 283 | There were no more free seats so he sat on… | Il n'y avait plus de sièges libres, alors il s'est assis sur… | No había más asientos libres así que él se sentó en… | sol: 30%, marché: 25%, strapontin: 10%, other(s): 35% | suelo: 55%, escalera: 25%, NV: 10%, other(s): 10% | sol: 55%, escalier: 15%, NV: 5%, other(s): 25% | sol: 70%, table: 10%, terre: 10%, other(s): 10% |
| 284 | To look for a job, Mario/Arthur knew he needed… | Pour chercher du travail, Arthur savait qu'il devait préparer… | Para buscar trabajo, Mario sabía que necesitaba… | curriculum vitae: 90%, candidature: 5%, entretien: 5% | curriculum vitae: 75%, NV: 5%, internet: 5%, other(s): 15% | curriculum vitae: 70%, cursus: 5%, entretien: 5%, other(s): 20% | curriculum vitae: 65%, NV: 10%, résumé: 10%, other(s): 15% |
| 285 | The woman bought lettuce and tomato to prepare… | La femme a acheté de la laitue et des tomates pour préparer… | La mujer compró lechuga y tomate para preparar… | salade: 95%, kebab: 5% | ensalada: 95%, hamburguesa: 5% | salade: 100% | salade: 80%, déjeuner: 5%, hamburger: 5%, other(s): 10% |
| 286 | To keep something from her trip to London, before returning, she went to a shop to buy… | Pour garder quelque chose de son voyage à Londres, avant de rentrer elle est allée dans un magasin acheter… | Para guardar algo de su viaje a Londres, antes de regresar fue a una tienda a comprar… | souvenir: 85%, cheddar: 5%, mug: 5%, ours: 5% | souvenir: 60%, recuerdo: 20%, bolsa: 5%, other(s): 15% | souvenir: 70%, valise: 15%, NV: 5%, other(s): 10% | souvenir: 80%, NV: 5%, chose: 5%, other(s): 10% |
| 287 | She has so much pain in her jaw that they will probably have to remove… | Elle a de telles douleurs dans la mâchoire, elle va sûrement devoir se faire enlever… | Ella tiene tanto dolor en la mandíbula que probablemente le tendrán que sacar… | dent: 95%, carie: 5% | muela: 90%, diente: 5%, pieza: 5% | dent: 40%, molaire: 20%, NV: 10%, other(s): 30% | dent: 70%, appareil: 5%, attelle: 5%, other(s): 20% |
| 288 | When biking, it’s safest to ride on… | À vélo, le plus sûr est de rouler sur… | Al ir en bici, lo más seguro es circular por… | piste: 45%, côté: 15%, trottoir: 10%, other(s): 30% | carril: 65%, derecha: 25%, carretera: 5%, centro: 5% | piste: 25%, trottoir: 20%, route: 15%, other(s): 40% | piste: 25%, chemin: 15%, route: 15%, other(s): 45% |
| 289 | He didn’t understand the math problem well, but doesn’t want to admit he made… | Il a mal compris le problème de maths, mais ne veut pas reconnaître qu'il a fait… | No entendió bien el problema de matemáticas, pero no quiere reconocer que cometió… | erreur: 95%, faute: 5% | error: 100% | erreur: 80%, NV: 5%, examen: 5%, other(s): 10% | erreur: 70%, NV: 15%, faute: 15% |
| 290 | When one spends a lot of time sitting, it’s important to maintain good posture to avoid pain in… | Si on passe beaucoup de temps assis, il est important de maintenir une bonne posture pour éviter les douleurs dans… | Cuando uno pasa mucho tiempo sentado, es importante mantener una buena postura para evitar los dolores en… | dos: 100% | espalda: 90%, cervical: 5%, lumbar: 5% | dos: 100% | dos: 85%, épaule: 10%, cou: 5% |
| 291 | This morning he went to the supermarket and, since he was in a hurry, the cashier helped him put the groceries in… | Ce matin, il est allé au supermarché et, comme il était pressé, la caissière l'a aidé à mettre les courses dans… | Esta mañana fue al supermercado y, como tenía prisa, la cajera le ayudó a meter la compra en… | sac: 55%, caddie: 20%, chariot: 10%, other(s): 15% | bolsa: 95%, carrito: 5% | sac: 70%, bourse: 10%, chariot: 5%, other(s): 15% | sac: 85%, NV: 10%, chariot: 5% |
| 292 | He likes to stay on the beach at the end of the day, looking west, to see … on the horizon | Il aime rester sur la plage en fin de journée, regardant à l'ouest, pour voir à l'horizon… | Le gusta quedarse en la playa al final del día, mirando al oeste, para ver en el horizonte… | coucher: 65%, soleil: 20%, NV: 10%, bateau: 5% | puesta: 50%, atardecer: 25%, NV: 5%, other(s): 20% | coucher: 55%, soleil: 20%, NV: 15%, other(s): 10% | coucher: 50%, soleil: 35%, bateau: 5%, other(s): 10% |
| 293 | He had a tennis match but couldn’t play because he had forgotten… | Il devait jouer au tennis mais n'a pas pu le faire car il avait oublié… | Él tenía un partido de tenis pero no pudo jugar porque se había dejado… | raquette: 100% | raqueta: 100% | raquette: 95%, équipement: 5% | raquette: 85%, balle: 5%, eau: 5%, vêtement: 5% |
| 294 | He approached the bins to throw away… | Il est allé à la benne pour y jeter… | Se acercó a los contenedores para tirar… | ordure: 40%, poubelle: 30%, déchet: 15%, other(s): 15% | basura: 100% | poubelle: 55%, déchet: 30%, chose: 5%, other(s): 10% | déchet: 45%, ordure: 15%, poubelle: 15%, other(s): 25% |
| 295 | He graduated as a teacher, so in September he will start working at… | Il vient d'être diplomé comme instituteur, donc en septembre il commencera à travailler dans… | Se ha diplomado como profesor, así que en septiembre empezará a trabajar en… | école: 80%, collège: 10%, enseignement: 5%, institut: 5% | colegio: 30%, escuela: 30%, instituto: 25%, other(s): 15% | école: 60%, collège: 15%, lycée: 10%, other(s): 15% | école: 65%, lycée: 10%, université: 10%, other(s): 15% |
| 296 | In most children’s stories, kids get lost in… | Dans la plupart des contes pour enfants, les enfants se perdent dans… | En la mayoría de cuentos infantiles, los niños se pierden en… | forêt: 75%, bois: 20%, imaginaire: 5% | bosque: 90%, detalle: 5%, final: 5% | forêt: 55%, bois: 10%, début: 10%, other(s): 25% | forêt: 65%, bois: 25%, imagination: 5%, rayon: 5% |
| 297 | For the awards ceremony at the theater, they put on the floor… | Pour la cérémonie de remise des prix au théâtre, ils ont installé sur le sol… | Para la entrega de premios en el teatro, pusieron en el suelo… | tapis: 70%, estrade: 10%, lumière: 5%, other(s): 15% | alfombra: 85%, galardón: 5%, marca: 5%, silla: 5% | tapis: 45%, NV: 5%, chaise: 5%, other(s): 45% | tapis: 50%, scène: 10%, NV: 5%, other(s): 35% |
| 298 | The girl has new colored pencils and has painted… | La petite fille a de nouveaux crayons de couleur et a fait… | La niña tiene lápices de colores nuevos y ha pintado… | dessin: 90%, coloriage: 5%, tri: 5% | dibujo: 40%, NV: 15%, arcoiris: 10%, other(s): 35% | dessin: 95%, peinture: 5% | dessin: 85%, peinture: 10%, image: 5% |
| 299 | The creatures from “Avatar” are easily distinguished from humans by… | Les créatures d'”Avatar” se différencient facilement des humains pour… | Las criaturas de "Avatar" se diferencian fácilmente de los humanos por… | NV: 25%, couleur: 25%, peau: 15%, other(s): 35% | color: 70%, piel: 20%, apariencia: 5%, tamaño: 5% | couleur: 70%, peau: 15%, NV: 5%, other(s): 10% | peau: 45%, extra-terrestre: 15%, couleur: 10%, other(s): 30% |
| 300 | Grandma doesn’t walk very well, to get around she needs… | La grand-mère ne marche plus très bien, pour se déplacer elle besoin… | La abuela no anda muy bien, para desplazarse necesita… | canne: 45%, déambulateur: 40%, NV: 5%, other(s): 10% | andador: 40%, bastón: 35%, tacataca: 10%, other(s): 15% | aide: 20%, canne: 20%, béquille: 15%, other(s): 45% | canne: 30%, bâton: 25%, fauteuil: 25%, other(s): 20% |
| 301 | When her husband used to stay late at the office, Joana started to suspect he was having… | Lorsque son mari a pris l'habitude de rester tard au bureau, Joana a commencé à soupçonner qu'il avait… | Cuando su marido se solía quedar hasta tarde en la oficina, Joana empezó a sospechar que estaba teniendo… | maîtresse: 40%, liaison: 35%, amant: 15%, other(s): 10% | aventura: 85%, affair: 5%, amorío: 5%, romance: 5% | affaire: 40%, amant: 35%, NV: 10%, other(s): 15% | liaison: 40%, affaire: 20%, femme: 15%, other(s): 25% |
| 302 | At the bar, whenever Diego/Didier drank too much, he became aggressive and tried to start… | Dans le bar, chaque fois que Didier buvait trop, il devenait agressif et essayait de créer… | En el bar, siempre que Diego bebía demasiado se volvía agresivo e intentaba empezar… | bagarre: 40%, dispute: 15%, embrouille: 10%, other(s): 35% | pelea: 60%, discusio2n: 20%, NV: 15%, jaleo: 5% | lutte: 30%, problème: 20%, combat: 10%, other(s): 40% | problème: 30%, bagarre: 25%, combat: 15%, other(s): 30% |
| 303 | He and she sat together in class without saying… | Lui et elle se sont assis ensemble en classe sans se dire… | Él y ella se sentaron juntos en clase sin decir ni… | mot: 60%, bonjour: 40% | palabra: 45%, NV: 30%, pío: 25% | rien: 60%, mot: 35%, NV: 5% | mot: 60%, NV: 15%, rien: 15%, other(s): 10% |
| 304 | The teacher wrote the sentence on… | Le professeur a écrit la phrase sur… | El profesor escribió la frase en… | tableau: 100% | pizarra: 85%, cuaderno: 5%, ipad: 5%, latín: 5% | tableau: 85%, ardoise: 15% | tableau: 90%, livre: 5%, papier: 5% |
| 305 | While knitting, the kittens didn’t stop playing with… | Pendant qu'elle tricotait, les chatons n'arrêtaient pas de jouer avec… | Mientras tejía, los gatitos no paraban de jugar con… | pelote: 55%, fil: 20%, laine: 15%, other(s): 10% | ovillo: 45%, lana: 40%, madeja: 10%, tela: 5% | pelote: 30%, laine: 20%, balle: 10%, other(s): 40% | laine: 35%, pelote: 30%, boule: 10%, other(s): 25% |
| 306 | It was windy enough to go to the park and fly… | Il y avait suffisamment de vent pour aller au parc et faire voler… | Hacía suficiente viento para ir al parque y hacer volar… | cerf-volant: 100% | cometa: 100% | cerf-volant: 45%, comete: 35%, NV: 5%, other(s): 15% | cerf-volant: 90%, air: 5%, frisbee: 5% |
| 307 | Olga’s/Cholé’s pants were too big, she had to buy… | Les pantalons de Chloé lui étaient trop grands, elle a dû acheter… | A Olga le iban grandes los pantalones, se tenía que comprar… | ceinture: 50%, pantalon: 20%, NV: 10%, other(s): 20% | cinturón: 40%, talla: 35%, NV: 25% | taille: 35%, NV: 30%, ceinture: 20%, other(s): 15% | ceinture: 35%, taille: 20%, pantalon: 15%, other(s): 30% |
| 308 | Raúl/Eric swept the dirt off the floor with… | Eric a balayé la poussière du sol avec… | Raúl barrió la suciedad del suelo con… | balai: 100% | escoba: 85%, cepillo: 10%, fregona: 5% | balai: 60%, serpillière: 10%, NV: 5%, other(s): 25% | balai: 70%, balayeur: 5%, bras: 5%, other(s): 20% |
| 309 | She worked many hours for little money, but her boss didn’t want to give her… | Elle travaillait de longues heures pour peu d'argent, mais son patron ne voulait pas lui accorder… | Ella trabajaba muchas horas por poco dinero, pero su jefe no quería darle… | augmentation: 70%, promotion: 10%, congés: 5%, other(s): 15% | aumento: 50%, ascenso: 15%, subida: 10%, other(s): 25% | augmentation: 60%, NV: 10%, hausse: 10%, other(s): 20% | augmentation: 50%, NV: 10%, argent: 10%, other(s): 30% |
| 310 | Raquel/Elsa woke up startled after having had… | Elsa s'est réveillée en sursaut après avoir fait… | Raquel se despertó sobresaltada después de haber tenido… | cauchemar: 80%, rêve: 20% | pesadilla: 100% | cauchemar: 30%, sieste: 15%, bruit: 10%, other(s): 45% | cauchemar: 65%, bruit: 5%, devoir: 5%, other(s): 25% |
| 311 | The old man goes for a walk every day with… | Le vieil homme se promène tous les jours avec… | El hombre mayor pasea todos los días con… | chien: 55%, canne: 15%, femme: 15%, other(s): 15% | perro: 45%, bastón: 15%, esposa: 10%, other(s): 30% | chien: 75%, canne: 15%, femme: 10% | chien: 55%, femme: 30%, bâton: 5%, other(s): 10% |
| 312 | In summer, the family cooked all kinds of meat on… | En été, la famille cuisinait toutes sortes de viandes sur… | En verano, la familia cocinaba todo tipo de carnes en… | barbecue: 65%, grill: 20%, NV: 5%, other(s): 10% | barbacoa: 60%, parrilla: 25%, asador: 5%, other(s): 10% | barbecue: 35%, grill: 30%, poêle: 10%, other(s): 25% | barbecue: 80%, grill: 15%, feu: 5% |
| 313 | Javier/Eliot put the basketball in… | Eliot a mis le ballon de basket dans… | Javier metió la pelota de baloncesto en… | panier: 90%, placard: 5%, sac: 5% | canasta: 70%, aro: 10%, mochila: 10%, other(s): 10% | panier: 50%, NV: 10%, centre: 5%, other(s): 35% | cerceau: 15%, fîlet: 15%, boîte: 10%, other(s): 60% |
| 314 | The address they gave him didn’t match any street in… | L'adresse qui lui avait été donnée ne correspondait à aucune rue sur… | La dirección que le habían dado no coincidía con ninguna calle en… | carte: 65%, plan: 25%, application: 5%, gps: 5% | ciudad: 45%, mapa: 20%, pueblo: 10%, other(s): 25% | ville: 35%, carte: 25%, plan: 10%, other(s): 30% | carte: 60%, quartier: 10%, google: 5%, other(s): 25% |
| 315 | To lose weight, Sofía/Sophie decided to commit to… | Pour perdre du poids, Sophie a décidé de se lancer dans… | Para perder peso, Sofía decidió comprometerse con… | régime: 35%, sport: 20%, course: 10%, other(s): 35% | dieta: 70%, ejercicio: 10%, gimnasio: 10%, other(s): 10% | régime: 50%, gymnase: 15%, diète: 10%, other(s): 25% | régime: 45%, abonnement: 5%, bain: 5%, other(s): 45% |
| 316 | The elderly woman bought potatoes and eggs because she wanted to prepare… | La femme âgée a acheté des pommes de terre et des œufs car elle voulait préparer… | La mujer mayor compró patatas y huevos porque quería preparar… | omelette: 40%, repas: 15%, gratin: 10%, other(s): 35% | tortilla: 90%, comida: 5%, ensalada: 5% | omelette: 70%, dîner: 5%, gâteau: 5%, other(s): 20% | omelette: 40%, repas: 15%, tortilla: 10%, other(s): 35% |
| 317 | She loved going to the beach and building castles with… | Il adorait aller à la plage et construire des châteaux dans… | Le encantaba ir a la playa y construir castillos con… | sable: 95%, dune: 5% | arena: 70%, cubo: 10%, amigo: 5%, other(s): 15% | sable: 90%, arène: 5%, rivage: 5% | sable: 100% |
| 318 | The woman wanted to hang her new coat in the closet but couldn’t find… | La femme voulait accrocher son nouveau manteau dans le placard mais ne trouvait pas… | La mujer quería colgar su abrigo nuevo en el armario pero no encontraba… | cintre: 85%, place: 10%, portemanteau: 5% | percha: 70%, sitio: 20%, hueco: 10% | cintre: 65%, bouton: 5%, clé: 5%, other(s): 25% | cintre: 35%, espace: 30%, place: 20%, other(s): 15% |
| 319 | The grass in the garden had grown a lot, his mother had asked him to run… | L'herbe dans le jardin avait beaucoup poussé, sa mère lui avait demandé de passer… | La hierba del jardín había crecido mucho, su madre le había pedido que pasara… | tondeuse: 90%, fil: 5%, NV: 5% | cortacésped: 90%, segador: 5%, segadora: 5% | tondeuse: 65%, NV: 5%, action: 5%, other(s): 25% | tondeuse: 35%, NV: 15%, pelouse: 10%, other(s): 40% |
| 320 | When putting on the suit, he gave the tie to his father so he could make him a… | En mettant son costume, il a donné la cravate à son père pour qu'il lui fasse… | Al ponerse el traje, le dio la corbata a su padre para que le hiciese… | noeud: 95%, cravate: 5% | nudo: 100% | noeud: 90%, attacher: 5%, nouer: 5% | noeud: 45%, cravate: 15%, NV: 10%, other(s): 30% |
| 321 | She was crying while chopping vegetables, but she wasn’t sad, she was just cutting… | Elle pleurait en préparant les légumes, mais elle n´était pas triste, elle était seulement en train de couper… | Estaba llorando mientras preparaba verduras, pero no estaba triste, simplemente estaba cortando… | oignon: 100% | cebolla: 100% | oignon: 90%, NV: 10% | oignon: 100% |
| 322 | She left crumbs all over the floor, so to pick them up she went to get… | Elle a mis des miettes partout sur le sol, donc pour les ramasser, elle est allée chercher… | Ella dejó migas por todo el suelo, así que para recogerlas ella fue a buscar… | balai: 75%, aspirateur: 20%, balayette: 5% | escoba: 55%, aspiradora: 15%, cepillo: 15%, recogedor: 15% | balai: 60%, cueilleur: 10%, aspirateur: 5%, other(s): 25% | balai: 55%, aspirateur: 15%, lunette: 10%, other(s): 20% |
| 323 | The woman had too much cash, so she decided to deposit it all in… | Comme elle avait trop d'argent liquide, la femme a décidé de tout déposer dans… | La mujer tenía demasiado dinero en efectivo así que decidió depositarlo todo en… | banque: 35%, compte: 30%, coffre: 20%, other(s): 15% | banco: 75%, cuenta: 20%, cajero: 5% | banque: 70%, compte: 20%, bouteille: 5%, tableau: 5% | banque: 60%, compte: 40% |
| 324 | When trying to pay with a credit card, he couldn’t remember… | En voulant payer avec sa carte de crédit, il ne savait soudain plus… | Al intentar pagar con tarjeta de crédito, no podía recordar… | code: 100% | pin: 50%, número: 25%, clave: 20%, contraseña: 5% | code: 30%, pin: 30%, NV: 15%, other(s): 25% | code: 55%, NV: 15%, argent: 15%, other(s): 15% |
| 325 | The businessman fired the employee when he ran out of… | L'employeur a renvoyé le travailleur lorsqu'il est arrivé à la fin de… | El empresario despidió al trabajador cuando se le terminó… | contrat: 55%, journée: 15%, période: 10%, other(s): 20% | contrato: 75%, paciencia: 10%, baja: 5%, other(s): 10% | journée: 30%, contrat: 20%, jour: 10%, other(s): 40% | journée: 30%, réunion: 15%, contrat: 5%, other(s): 50% |
| 326 | The hen sat on the stone, warming it as if it were… | La poule s'est posée sur la pierre, la réchauffant comme si c'était… | La gallina se colocó sobre la piedra, dándole calor como si fuese… | œuf: 75%, radiateur: 10%, soleil: 10%, brique: 5% | huevo: 75%, estufa: 15%, polluelo: 10% | œuf: 45%, nid: 20%, NV: 5%, other(s): 30% | œuf: 70%, NV: 10%, chien: 5%, other(s): 15% |
| 327 | Probably the most popular aspect of the Mona Lisa painting is… | L'aspect le plus populaire du tableau de Monna Lisa est probablement… | Probablemente, el aspecto más popular del cuadro de la Mona Lisa es… | sourire: 45%, regard: 30%, manque: 5%, other(s): 20% | sonrisa: 40%, NV: 15%, brazo: 5%, other(s): 40% | sourire: 65%, visage: 15%, louvre: 10%, other(s): 10% | sourire: 50%, visage: 15%, femme: 10%, other(s): 25% |
| 328 | In the Cinderella story, the princess has to run and that’s why she loses… | Dans l'histoire de Cendrillon, la princesse doit courir et elle perd… | En el cuento de la Cenicienta, la princesa tiene que correr y por eso pierde… | chaussure: 55%, soulier: 20%, pantoufle: 10%, other(s): 15% | zapato: 100% | chaussure: 85%, soulier: 10%, talon: 5% | chaussure: 65%, pantoufle: 25%, cheveu: 5%, talon: 5% |
| 329 | She's going on a trip with so many things that she can't fit everything in... | Elle part en voyage avec tellement de choses que ça ne tient pas tout dans… | Ella se va de viaje con tantas cosas que no le cabe todo en… | valise: 85%, sac: 10%, bagage: 5% | maleta: 95%, bolso: 5% | valise: 80%, coffre: 10%, voiture: 10% | valise: 75%, sac: 10%, NV: 5%, other(s): 10% |
| 330 | While he was sailing, he saw an animal jumping out of the water and thought it must be… | Alors qu'il naviguait, il a vu un animal sauter hors de l'eau et a pensé que cela devait être… | Mientras él navegaba vio un animal saltando fuera del agua y pensó que debía ser… | dauphin: 35%, poisson: 25%, baleine: 10%, other(s): 30% | delfín: 65%, pez: 15%, rana: 10%, other(s): 10% | dauphin: 35%, poisson: 35%, saumon: 10%, other(s): 20% | dauphin: 65%, poisson: 15%, NV: 5%, other(s): 15% |

*Note*. Ellipses indicate the gap left for participants to fill. ‘NV’ indicates non-valid answers.

**Appendix B**

**Results Using Cloze Probability as a Measure of Sentence Constraint**

**Figure B1**

*Cloze Probability Distributions for French- and Spanish-Dominant Speaker Groups*

**Figure B2**

*Cloze Probability Alignment Between French- and Spanish-Dominant Speaker Groups*

| **A**   | **B**   |
| --- | --- |

**Figure B3**

*Cloze Probability Distributions for French-Dominant, Spanish-French and English-French Bilingual Speaker Groups*

**Figure B4**

*Cloze Probability Alignment Between French-Dominant and Spanish-French Bilingual Speaker Groups*

| **A**   | **B**   |
| --- | --- |

**Figure B5**

*Cloze Probability Alignment Between French-Dominant and English-French Bilingual Speaker Groups*

| **A**   | **B**   |
| --- | --- |

**Figure B6**

*Cloze Probability Alignment Between Spanish-French and English-French Bilingual Speaker Groups*

| **A**   | **B**   |
| --- | --- |

**Appendix C**

**Relationship Between Response Entropy and Cloze Probability for Each Group**

**Figure C1**

*French-Dominant Group Completing Sentences in French*

**Figure C2**

*Spanish-Dominant Group Completing Sentences in Spanish*

**Figure C3**

*Spanish-French Bilingual Group Completing Sentences in French*

**Figure C4**

*English-French Bilingual Group Completing Sentences in French*
